# Intermittent ERK oscillations downstream of FGF in mouse embryonic stem cells

**DOI:** 10.1242/dev.199710

**Published:** 2022-02-17

**Authors:** Dhruv Raina, Fiorella Fabris, Luis G. Morelli, Christian Schröter

**Affiliations:** 1 Department of Systemic Cell Biology, Max Planck Institute of Molecular Physiology, Otto-Hahn-Str. 11, 44227 Dortmund, Germany; 2 Instituto de Investigación en Biomedicina de Buenos Aires (IBioBA)–CONICET–Partner Institute of the Max Planck Society, Polo Científico Tecnológico, Godoy Cruz 2390, C1425FQD Buenos Aires, Argentina; 3 Departamento de Física, FCEyN UBA, Ciudad Universitaria, 1428 Buenos Aires, Argentina

**Keywords:** FGF/ERK signaling, Cell cycle, Mouse embryonic stem cells, Oscillations, Signal transduction networks, Time series analysis

## Abstract

Signal transduction networks generate characteristic dynamic activities to process extracellular signals and guide cell fate decisions such as to divide or differentiate. The differentiation of pluripotent cells is controlled by FGF/ERK signaling. However, only a few studies have addressed the dynamic activity of the FGF/ERK signaling network in pluripotent cells at high time resolution. Here, we use live cell sensors in wild-type and *Fgf4-*mutant mouse embryonic stem cells to measure dynamic ERK activity in single cells, for defined ligand concentrations and differentiation states. These sensors reveal pulses of ERK activity. Pulsing patterns are heterogeneous between individual cells. Consecutive pulse sequences occur more frequently than expected from simple stochastic models. Sequences become more prevalent with higher ligand concentration, but are rarer in more differentiated cells. Our results suggest that FGF/ERK signaling operates in the vicinity of a transition point between oscillatory and non-oscillatory dynamics in embryonic stem cells. The resulting heterogeneous dynamic signaling activities add a new dimension to cellular heterogeneity that may be linked to divergent fate decisions in stem cell cultures.

## Abstract (precis)

**Summary:** High time-resolution measurements reveal previously unseen signaling dynamics in single pluripotent cells, which alternate between oscillatory and non-oscillatory ERK activity. ERK dynamics change with ligand concentration, cell cycle and differentiation state.

## INTRODUCTION

Cells rely on signal transduction networks to process signals from their environment, and to guide decisions such as to divide, differentiate or die (Koseska and Bastiaens, 2017). These networks can produce dynamic activation patterns even at constant stimuli (Antebi et al., 2017; Santos et al., 2007). Dynamic activity patterns are shaped by the cell-type specific architecture of the signal transduction system.

One of the most crucial signal transduction systems during early mammalian embryogenesis relays signals from extracellular fibroblast growth factor 4 (FGF4) through the RAS/RAF/MEK/ERK network (Brewer et al., 2016). The differentiation of extra-embryonic primitive endoderm cells in the mouse preimplantation embryo depends on FGF/ERK signaling in a dose-dependent manner (Kang et al., 2013; Krawchuk et al., 2013). Embryonic stem cells (ESCs), clonal cell populations that retain the differentiation potential of inner cell mass cells of the preimplantation embryo, are a tractable model system that recapitulates this dose-dependent function of FGF4 (Raina et al., 2021; Schröter et al., 2015). FGF/ERK signaling is also required for maturation of the epiblast lineage in the embryo (Kang et al., 2017; Ohnishi et al., 2014), and controls the corresponding process of transitioning from naïve to primed pluripotency and lineage commitment in ESCs (Kunath et al., 2007; Molotkov et al., 2017). Both in the embryo and ESCs, FGF/ERK signaling is mostly triggered by paracrine FGF4 ligands (Kang et al., 2013; Krawchuk et al., 2013; Kunath et al., 2007). Despite these well-known functions of FGF/ERK signaling during the differentiation of pluripotent cells, little is known about FGF/ERK signaling dynamics in this developmental context.

Revealing intracellular signal transduction dynamics requires live-cell approaches in single cells. Live-cell ERK activity can be monitored with substrate-based sensors that employ fluorescence resonance energy transfer (FRET) or subcellular localization as read-outs (Komatsu et al., 2011; Regot et al., 2014). Analysis of ERK activity in acutely stimulated ESCs expressing a FRET-based sensor revealed a transient peak of activation that decayed over long timescales (Deathridge et al., 2019). However, the short timescale ERK signaling dynamics in the continuous FGF stimulation regimes required to trigger differentiation of ESCs (Hamilton et al., 2019) remain largely unexplored.

In other cell types, short timescale ERK dynamics upon continuous stimulation of the epidermal growth factor (EGF) receptor show diverse behaviors. In many, but not all, cell types, ERK activity occurs in pulses (Aoki et al., 2013). In several cell types, the frequency of ERK activity pulses depends on EGF concentration or cell density (Albeck et al., 2013; Aoki et al., 2013). This has led to the suggestion of frequency-modulated encoding of information about extracellular signal levels by the RAS/RAF/MEK/ERK network downstream of the EGF receptor (Albeck et al., 2013). In mammary epithelial cells in contrast, pulses of ERK nuclear translocation have a constant frequency across a range of EGF stimulation levels (Shankaran et al., 2009).

Here, we use a translocation-based sensor (Regot et al., 2014) to measure short timescale ERK activity dynamics in single ESCs. We find that ERK activity is pulsatile in ESCs, and develop concepts and analysis methods to quantitatively characterize dynamic signatures of pulsing. ERK activity pulses in ESCs are faster than any previously reported ERK dynamics. In some cells, pulsing occurs in regular, consecutive sequences. Thus, we develop metrics to quantify pulsing regularity, and use these to contrast experimental observations with simple stochastic models. These stochastic models alone cannot explain experimental data. Together with other dynamic signatures, these observations suggest the presence of ERK oscillations. To determine how these oscillations change with environment and cell state, we measure ERK dynamics in response to defined concentrations of FGF4 ligands, along the cell cycle, and in different pluripotency states. We detect no pulsing in *Fgf4*-mutant cells, but pulsing is restored upon addition of recombinant FGF4, indicating that FGF4 triggers ERK pulsing. Controlling extracellular ligand levels in the mutant background, we show that individual ERK pulses have a duration that is independent of ligand levels. However, the extent of the oscillatory behavior increases with FGF4 dose. Using long-term recordings to follow cells from birth to division, we show that ERK pulsing is more prevalent in the early stages of the cell cycle. Finally, we compare ERK dynamics in embryonic and epiblast stem cells, and find that oscillations become less prevalent following differentiation. Taken together, our data suggest that the FGF/ERK signal transduction system in pluripotent cells transits between oscillatory and non-oscillatory behavior.

## RESULTS

### ERK activity is dynamic in ESCs

**Fig. 1.**
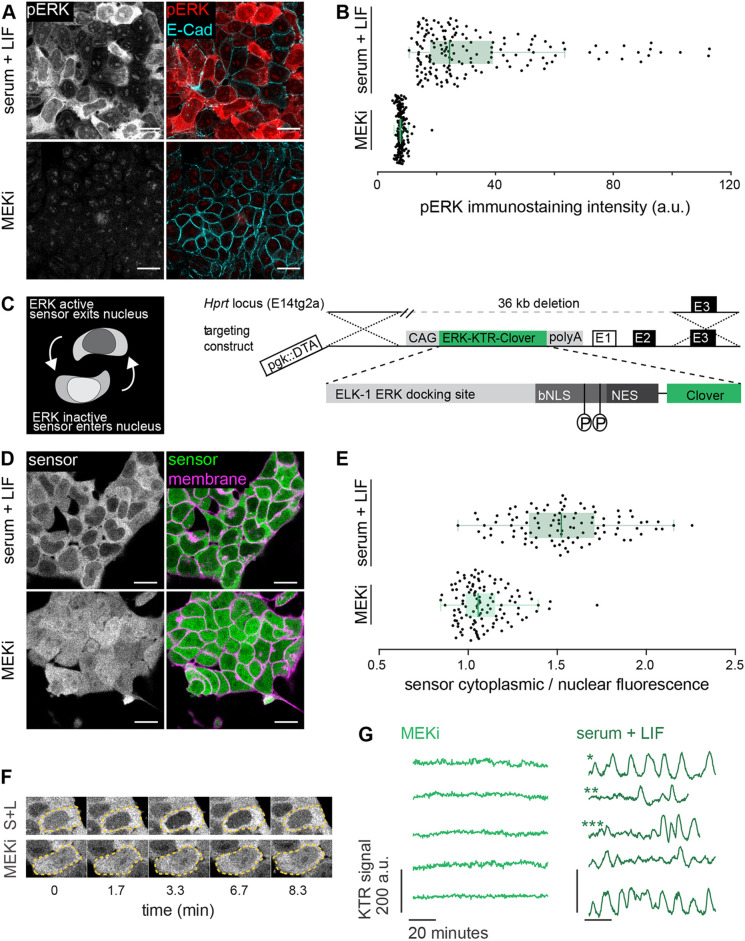
**A targeted translocation sensor reveals pulsatile ERK activity in ESCs.** (A) Immunostaining of mESCs growing in serum+LIF (S+L) medium without (top) or with (bottom) MEKi for pERK and E-cadherin to mark membranes. The punctate pERK staining within the nucleus is insensitive to MEK inhibition, suggesting it is non-specific. (B) Quantification of fluorescence staining intensities in single cells stained as in A. *n*≥100 per condition, green bars indicate medians (median_S+L_=24.39 a.u., median_MEKi_=7.75 a.u.; CV_S+L_=0.67, CV_MEKi_=0.17), box bounds are the 25 and 75 percentiles of the distributions, and whiskers are the 5 and 95 percentiles. (C) Schematic of the ERK-KTR sensor and targeting construct for integration into the *Hprt* locus. (D) Subcellular localization of ERK-KTR sensor in live cells in serum+LIF without (top) and with (bottom) MEKi. Membranes are stained with live-cell membrane dye CellMaskRed. (E) Quantification of cytoplasmic to nuclear ratio of sensor fluorescence in single cells imaged as in D. Green bars indicate medians (median_S+L_=1.52, median_MEKi_=1.05; CV_S+L_=0.18, CV_MEKi_=0.13), box bounds are the 25 and 75 percentiles of the distributions, and whiskers are the 5 and 95 percentiles. (F) Stills from a movie of ERK-KTR-expressing cells growing in serum+LIF without (top) and with (bottom) MEKi. Dashed line indicates cell outlines. (G) Representative traces of the KTR signal obtained as the mean inverted fluorescence intensity within a nuclear ROI in single cells growing in serum+LIF without (right) and with (left) MEKi. *, cell showing regular pulsing; ** cell showing isolated pulses; ***, cell showing transitions between non-pulsing and pulsing behavior. Scale bars: 20 μm.

We first explored ERK activation in single ESCs under constant culture conditions that maintain pluripotency. We stained for phosphorylated ERK (pERK) in cells growing in serum+LIF and quantified whole-cell pERK levels. We observed pERK staining in cells growing in serum+LIF, and noted it was absent in the presence of the MEK-inhibitor PD0325901 (MEKi) (Fig. 1A,B). pERK staining was more heterogeneous in serum+LIF than in the MEKi control. Almost all cells in serum+LIF had pERK staining values above the range covered by MEKi cells (Fig. 1B).

The heterogeneous pERK staining in serum+LIF could purely reflect long-term variability between cells as previously reported (Deathridge et al., 2019). In addition, short-term signaling fluctuations could contribute to this variability. To test the extent of short-term signaling fluctuations, we integrated a translocation-based sensor to measure ERK activity in live cells. We generated this cell line by single copy insertion of the ERK-KTR-mClover construct into the *Hprt* open locus (Fig. 1C) to ensure uniformity in expression. Transgenic cells continued to express pluripotency markers (Fig. S1) and transmitted to the germline of chimeric mice (Simon et al., 2020), indicating that reporter expression does not interfere with pluripotency and differentiation potential.

Phosphorylation of the ERK target site of the sensor leads to its export from the nucleus, thus reporting ERK activity as the cytoplasmic to nuclear (C/N) ratio of reporter localization (Regot et al., 2014) (Fig. 1C). Snapshots of cells growing in serum+LIF showed that the sensor preferentially localized to the cytoplasm, in contrast to the MEKi-treated control where it was uniformly distributed (Fig. 1D). Furthermore, the C/N ratio of sensor localization was more variable between cells growing in serum+LIF compared with the MEKi-treated control (Fig. 1E), in line with heterogeneous pERK staining. These qualitative similarities between pERK staining and reporter C/N ratios suggest that the reporter is suited to explore short-term ERK dynamics in ESCs.

We next recorded dynamic changes of reporter localization by imaging reporter cells at 20 s time intervals for up to 2 h. In these time-lapse movies we could observe repetitive translocation of the sensor back and forth from the nucleus of cells growing in serum+LIF, which were absent in MEKi (Fig. 1F; Movie 1). For quantification of dynamic activity in single cells, we measured KTR fluorescence intensity in regions of interest in the nucleus and cytoplasm of individual cells over time (Fig. S2; Materials and Methods). Drops in nuclear fluorescence were paralleled by surges in cytoplasmic fluorescence. Although the C/N ratio has been used as a read-out for ERK activity in other cell lines (Regot et al., 2014), the cytoplasm in ESCs is small and often difficult to identify unambiguously. We therefore compared the scaled C/N ratio from these measurements with the nuclear signal from the inverted image, and found that they were very similar (Fig. S2). Thus, for convenience, we focused on sensor fluorescence in the nucleus.

To validate that translocation of the sensor out of the nucleus reflected genuine ERK activity, we transfected two spectrally compatible orthogonal ERK activity sensors in the same cells. Both sensors showed similar and highly correlated dynamic behavior (Fig. S3; Materials and Methods). These sensors rely on different ERK substrate sequences, and deploy FRET (Komatsu et al., 2011) and translocation as two distinct read-outs. This indicates that pulsatile nuclear export of the KTR sensor reflects genuine ERK dynamics.

As active ERK resulted in sensor export out of the nucleus, we defined the nuclear intensity of the inverted image as the KTR signal, such that high values of the KTR signal reflect high ERK activity. This representation revealed a broad range of dynamic behaviors across the population (Fig. 1G; Fig. S4): some cells showed regular pulsing (*, Fig. 1G), and some showed isolated pulses (**, Fig. 1G). We also observed transitions between non-pulsing and pulsing behavior within the same cell (***, Fig. 1G). We conclude that pluripotent ESCs cultured in serum+LIF display a range of pulsatile ERK activity dynamics.

### Intermittent ERK oscillations in ESCs

**Fig. 2.**
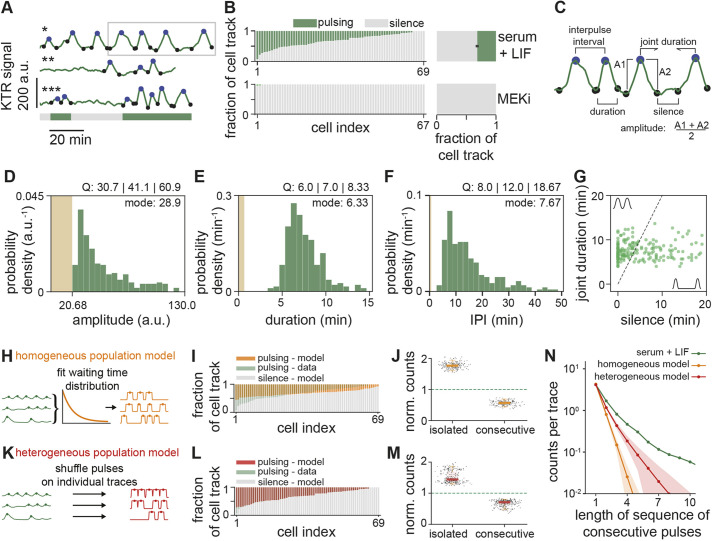
**Time series analysis reveals intermittent ERK oscillations in ESCs.** (A) Pulse recognition in representative time series of ERK dynamical activity. Shown are smoothened single cell traces of KTR signal in the serum+LIF condition. Pulses are indicated by the maxima (blue dots) and corresponding minima (black dots) that define them. Bar at the bottom indicates pulsing (green) or non-pulsing (gray) intervals in the lower trace. (B) Left: fraction of time that individual cells spent pulsing (green) or non-pulsing (gray) in serum+LIF alone (top) or upon addition of MEKi (bottom). Right: average time that cells were pulsing (green) or non-pulsing (gray) in the cell population. Error bar indicates s.e.m. (C) Dynamical features of the time series analyzed in D-G are indicated on a sample trace portion (gray rectangle in A). (D) Pulse amplitude distribution for the serum+LIF condition (*n*=289 pulses). (E) Pulse duration distribution for the serum+LIF condition (*n*=289 pulses). (F) Interpulse interval (IPI) distribution for the serum+LIF condition (*n*=225 pairs of pulses). Pulse recognition resolution limit (brown bar) and quartiles (Q) 25, 50 and 75 are indicated in D-F. (G) Joint pulse duration versus silence intervals for successive pairs of pulses in the serum+LIF condition (*n*=225 pairs of pulses). The slope 2 dashed line classifies pairs of pulses into consecutive (above) and non-consecutive (below). The axes range was adjusted to better resolve individual data points, leaving off the scale 27 out of 225 data points. Data in D-G from *N*=69 cells. (H-J) Homogeneous population model. (H) Schematic of homogeneous population model for stochastic pulsing. Experimental data (left, green) was used to fit an exponential waiting time distribution of the entire population (middle), from which simulated stochastic traces were obtained (right, orange). (I) Fraction of time that individual simulated traces spent pulsing (orange), compared with experimental data from B (green). (J) Counts of isolated and consecutive pulses from 200 realizations of the model divided by corresponding counts in experimental traces (black dots). A single realization is composed of *N*=69 traces. (K-M) Heterogeneous population model. (K) Schematic of heterogeneous population model for stochastic pulsing. Pulses in individual experimental traces (left, green) were randomly repositioned to generate shuffled traces (right, red). (L) Fraction of time that cells spent pulsing (red). (M) Normalized counts of isolated and consecutive pulses from 200 model realizations, each consisting of *N*=69 shuffled traces. (N) Number of pulse trains as a function of the number of consecutive pulses in the train for experimental data (green), homogeneous population model (orange) and heterogeneous population model (red). The count includes instances that occur within longer trains, and the first data point corresponds to the total number of individual pulses. Counts have been normalized by the number of traces. Shaded area is the s.d. from 200 independent realizations of the stochastic models. Color bars in J and M represent the median, box bounds are the 25 and 75 percentiles of the distributions, and whiskers are the 5 and 95 percentiles.

The broad range of dynamic behaviors that we observed qualitatively across the population prompted us to systematically investigate the dynamic signatures of ERK activity in ESCs. As ERK activity pulses were a prominent feature of the dynamics, we sought to identify single pulses in time series. We first annotated the time points of local maxima and minima, and then used time series of MEKi-treated cells to set a threshold for filtering ERK-dependent pulses from background fluctuations (Fig. 2A; Fig. S5; Table S1; Materials and Methods). Most cells (64/69, 93%) showed pulses in serum+LIF, whereas very few (2/67, 3%) showed any pulse in MEKi. The total fraction of time that single cells were pulsing was variable: some cells pulsed continuously, others showed a mixture of pulsing and non-pulsing behavior – termed silent – and yet others were non-pulsing throughout the experiment (Fig. 2B). On average, cells were pulsing 32±3% (mean±s.e.m.) of the time in serum+LIF alone, but only 0.13±0.09% of the time in the presence of MEKi (Fig. 2B).

To determine general characteristics of pulsing activity in the population, we introduced a set of quantitative measures: the amplitude and duration of single pulses, and the interpulse and silence intervals between successive pulses (Fig. 2C). The amplitude of a pulse was defined as the average difference between the peak value and the neighboring local minima (Fig. 2C). Our thresholding parameters only filter the tail of the amplitude distribution, containing low amplitude fluctuations that fall within the range of background levels determined from time series of MEKi-treated cells (brown area in Fig. 2D). Thus, even though the quantitative relationship between ERK activity and pulse amplitude is not known, we can still faithfully distinguish genuine ERK pulses from background fluctuations.

We defined the duration of a pulse as the time elapsed between the two local minima flanking the maximum of the pulse (Fig. 2C; Materials and Methods). The distribution of pulse durations has a well-defined mode at 6.33 min and is slightly asymmetric (Fig. 2E). We observed no pulses shorter than 3 min, a timescale much longer than the detection limit of 40 s given by our algorithm and our sampling frequency. The congruence of the KTR and FRET sensors suggest that the pulse durations that we can capture are not limited by the timescales of sensor transport (Fig. S3). Therefore, we conclude that ERK pulses have a minimum duration. Pulses with long durations tended to have large amplitudes, and those with short durations clustered at low amplitude values (Fig. S6).

To further characterize the type of ERK dynamics in single cells, we extended our focus to quantify how pulses are arranged in traces. We defined the interpulse interval (IPI) as the time between the maxima of two neighboring pulses (Fig. 2C). The mode of the IPI distribution was 7.67 min, similar to the mode of the pulse duration (Fig. 2F). For the IPI to have a similar value as the durations of the neighboring pulses, a pulse has to begin immediately after the previous one. Thus, this similarity of the modes suggests the presence of consecutive pulses, occurring immediately one after another. We next set out to determine how often pulses were consecutive. Consecutive pulses have either shared minima, or are separated by intervals of silence that are short relative to their pulse duration. As each IPI can be decomposed into a silence interval and a joint pulse duration (Fig. 2C; Materials and Methods), we used these quantities to define consecutiveness in a way that accounts for differences in pulse duration. In a plot of joint duration against silence interval duration, sparse events will lie in the lower right region, whereas consecutive pulses will populate the upper left. Here, we defined pairs of consecutive pulses as those with a silent interval of less than half the joint pulse duration (dashed line, Fig. 2G). With this definition, 52% of all pairs of pulses in cells growing in serum+LIF lay above the threshold and were classified as consecutive (Fig. 2G).

The prevalence of consecutive pulsing hinted at an oscillatory behavior, which was further supported by autocorrelation analysis of the traces. The normalized autocorrelation function of individual traces in the serum+LIF displayed signatures of oscillations, albeit very variable, in contrast to a smooth decay for the MEKi condition (Fig. S7; Materials and Methods). However, the asymmetric shape of the IPI distribution raised the question of whether pulsing could still be stochastic. We tested this possibility by contrasting the data with a stochastic pulsing hypothesis. We considered a stochastic pulsing scenario in which waiting times between pulses are exponentially distributed. We thus plotted the distribution of the times between pulses and fitted an exponential distribution to the data (Fig. S8). Then, we generated traces from this fitted waiting time distribution and the experimentally determined distribution of pulse durations (Fig. 2H; Fig. S8; Materials and Methods). From these simulated traces, we first obtained the fraction of each individual cell track spanned by pulsing. Stochastic pulsing simulations failed to recapitulate the presence of cells that pulse for a large fraction of time (left, Fig. 2I), as well as cells that pulse for a very small fraction of time that were observed in the experiment (right, Fig. 2I). Furthermore, isolated pulses in simulations occur more often than in experimental traces, whereas consecutive pulses occur less often (Fig. 2J). Thus, a stochastic pulsing model with a single exponential distribution of waiting times across the population cannot explain these key features of the data.

Next, we asked whether each individual cell pulsed stochastically with its own waiting time distribution. To simulate this scenario, we randomized the position of pulses in individual experimental traces (Fig. 2K). With this approach, simulations matched the total fraction of time that single cells were pulsing, including cells that pulse for large and small fractions of time (Fig. 2L). However, isolated pulses in simulations still occurred more often than in experimental traces and consecutive pulses occurred less often (Fig. 2M). Longer sequences of consecutive pulses were likewise far more prevalent in the experimental data compared with both stochastic models (Fig. 2N). Thus, these stochastic pulsing scenarios are hard to reconcile with the data.

In summary, our analysis reveals that ERK pulses in ESCs growing in serum+LIF have a characteristic duration and are often part of consecutive sequences which we could not capture with simple stochastic models. We interpret this behavior as intermittent oscillations, where silent periods alternate with isolated pulses and oscillations – here defined as consecutive pulses with a characteristic duration.

### ERK oscillations are triggered by FGF4

ERK activity is dynamic in many cell types (Albeck et al., 2013; Aoki et al., 2017; de la Cova et al., 2017; Goglia et al., 2020; Hiratsuka et al., 2015; Mayr et al., 2018; Pokrass et al., 2020; Shankaran et al., 2009; Simon et al., 2020). Extracellular signals can change the characteristics of these dynamics, such as pulse frequency (Albeck et al., 2013; Aoki et al., 2013). In ESCs, FGF4 is the main ligand that activates ERK (Kunath et al., 2007). We therefore asked how ERK dynamics depend on FGF4 concentration. To be able to control FGF4 concentration externally, we introduced an *Fgf4* loss-of-function mutation in the sensor line. These *Fgf4*-mutant cells were viable and continued to divide in chemically defined N2B27 medium that contains only minimal amounts of recombinant growth factors (Movie 2). This is consistent with previous reports (Kunath et al., 2007) and demonstrates that FGF4 signaling is dispensable for cell cycle progression in ESCs. Still, pERK levels were strongly reduced in this line (Fig. S9). Even though pERK levels in the *Fgf4* mutant were similar to levels in wild-type cells treated with MEKi, the KTR sensor located preferentially to the cytoplasm, in contrast to acute MEKi treatment where it was evenly distributed (compare Fig. 1D with Movie 2). Thus, chronic deprivation from FGF4 in the mutant line leads to a pERK-independent shift of the sensor C/N ratio.

**Fig. 3.**
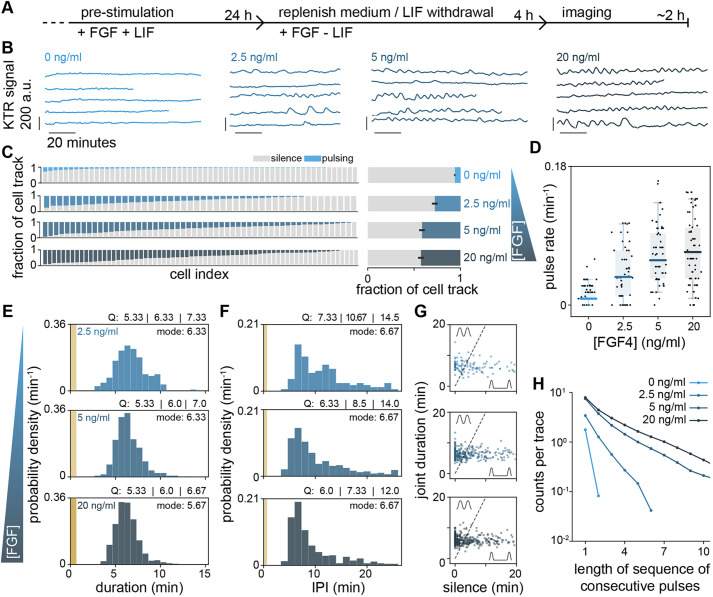
**Pulsing and regularity of ERK activity are controlled by FGF4 dose.** (A) Schematic of experimental protocol to measure the steady state signaling response to defined FGF4 ligand levels. (B) Representative smoothened single cell traces of KTR signal in single *Fgf4*-mutant cells stimulated with different FGF4 doses. (C) Left: fraction of time that individual cells stimulated with different concentrations of FGF4 spent pulsing (blue) or non-pulsing (gray). Right: average time that cells in the population were pulsing (blue) or non-pulsing (gray). Error bar indicates s.e.m. (D) Pulse rate boxplots at different concentrations of FGF4. Black dots represent individual cells, color bars are the median, box bounds are the 25 and 75 percentiles of the distributions, and whiskers are the 5 and 95 percentiles. (E) Pulse duration distributions. The number of pulses was *n*=164 (2.5 ng/ml), *n*=426 (5 ng/ml) and *n*=544 (20 ng/ml). (F) Distributions of interpulse intervals (IPI) between pairs of successive pulses. The number of successive pulses was *n*=124 (2.5 ng/ml), *n*=370 (5 ng/ml) and *n*=479 (20 ng/ml). Pulse recognition resolution limit (yellow bar) and quartiles (Q) 25, 50 and 75 are indicated in E and F. (G) Joint pulse duration versus silence intervals for successive pairs of pulses. The slope 2 dashed line classifies pairs of pulses into consecutive (above) and non-consecutive (below). The axes range was adjusted to better resolve individual data points, leaving off the scale six of 124 (2.5 ng/ml FGF4), 26 out of 370 (5 ng/ml FGF4) and 33 out of 479 (20 ng/ml FGF4) data points. (H) Number of pulse trains as a function of the number of consecutive pulses in the train for different doses of FGF4, determined as in Fig. 2N. Number of cells in C-H: *N*=61 (0 ng/ml FGF4), *N*=48 (2.5 ng/ml FGF4), *N*=57 (5 ng/ml FGF4) and *N*=69 (20 ng/ml FGF4).

For stimulating ERK activity, we chose FGF4 concentrations from 2.5 to 20 ng/ml. These concentrations cover the dynamic range of FGF4-response at the level of ERK phosphorylation and transcription of an FGF/ERK-dependent reporter gene (Fig. S9), as well as differentiation along the primitive endoderm lineage (Raina et al., 2021). To measure the steady-state signaling response to different ligand levels, we pre-treated cells with the respective FGF4 concentrations for 24 h in N2B27 supplemented with Chiron and LIF to maintain pluripotency. As LIF can activate ERK (Ohtsuka and Niwa, 2015), 4 h before starting the recording we replenished with N2B27 containing Chiron and FGF4, but lacking LIF (Fig. 3A; Materials and Methods). Under these conditions, FGF4 promotes differentiation towards embryonic lineages within 1-2 days, but cells are still pluripotent during the time of recording (Kalkan et al., 2017). In the absence of FGF4 stimulation, we observed almost no pulsing. Widespread pulsatile activity was observed at all FGF4 concentrations tested, indicating that FGF4 triggers ERK pulsing (Fig. 3B; Fig. S10; Movie 2). To identify pulses, we employed a similar strategy as above, setting a threshold based on the untreated condition and the highest FGF4 concentration (Fig. S11, Materials and Methods).

The distribution of sensor pulse amplitudes was not significantly different amongst the three concentrations (Fig. S12; Table S2; Materials and Methods). However, immunostaining and single cell analysis revealed that the median, the lower end, as well as the variance of the pERK distributions shifted to larger values with increasing FGF concentration (Fig. S12). Thus, it is possible that the amplitude of pERK pulses increases with FGF concentration, without translating into a measurable increase in sensor pulse amplitude.

The total fraction of time that single cells were pulsing increased with FGF4 concentration in the range from 0 to 5 ng/ml (Fig. 3C) to levels similar to those measured in wild-type cells in serum+LIF. We wondered how the number of pulses and their duration contributed to this increase in pulsing time. We defined a single cell pulse rate as the number of pulses divided by the duration of the trace, and found that it increases with FGF4 concentration in the same range (Fig. 3D). The distribution of pulse durations overlapped between the three FGF4 concentrations, and their modal values were conserved (Fig. 3E). We observed a subtle trend towards narrower distributions with higher FGF4 concentrations, yet these were significantly different only between the 2.5 ng/ml and 20 ng/ml conditions (Table S2; Materials and Methods). Thus, the increase in pulsing time is largely due to an increase in pulse rate rather than pulse duration. In line with stable pulse durations, the IPI distributions had a similar modal value of about 7 min in all conditions**.** However, IPIs became more narrowly distributed with increasing FGF4, with a clear difference between 2.5 and 20 ng/ml FGF4 (Fig. 3F; Table S2). Narrower IPI distributions at high FGF concentrations indicated more regular pulsing.

To determine whether the extent of consecutive pulsing was controlled by FGF4, we plotted joint pulse duration against silence interval duration (Fig. 3G). The fraction of pairs of consecutive pulses increased steadily across the entire FGF concentration range from 49.2% (2.5 ng/ml) to 57.6% (5 ng/ml) and 63.9% (20 ng/ml). Longer sequences of consecutive pulses were also more prevalent at higher FGF4 doses (Fig. 3H).

In summary, these results reveal that ERK pulses have a characteristic duration that is independent from FGF4 concentration. This characteristic duration becomes less variable with increasing FGF concentration. We interpret the increase in pulse rate, prevalence of consecutive pulsing and length of consecutive pulse sequences with FGF4 dose as an indication that FGF4 controls the extent of ERK oscillations in pluripotent ESCs.

### ERK pulses are more prevalent early in the cell cycle

**Fig. 4.**
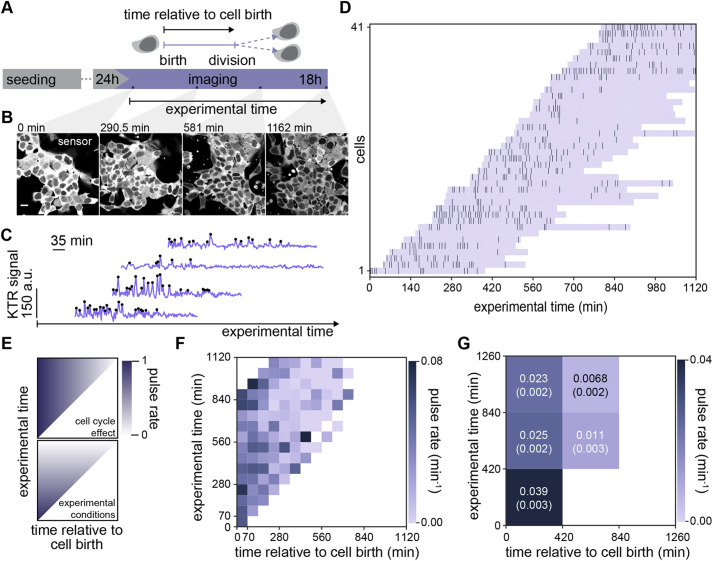
**ERK pulsing is more prevalent early in the cell cycle.** (A) Schematic of experimental protocol to record ERK peaks across complete cell cycles. (B) Montage of an ESC colony expressing the ERK-KTR sensor over the course of a long-term imaging experiment. (C) Representative filtered traces of ERK dynamical activity with identified peaks (black dots), in single wild-type cells growing in N2B27 medium. (D) Raster plot displaying the timing of ERK activity peaks across the cell cycle. Lavender horizontal bands extend from birth to division of single cells, dark vertical bars represent peaks. Single cell tracks begin immediately after a cell division event and are plotted relative to absolute experimental time. (E) Schematic representation of expectations for a reduction of pulsing activity due to cell cycle (top) and due to changing experimental conditions (bottom) in the 2-dimensional color-encoded pulse rate map. (F) Pulse rate map for the data shown in D. Time is discretized into 70 min bins. (G) Coarse grained pulse rate map showing average pulse rate and its estimated error with 420 min binning. Scale bar: 20 μm.

We noted that within the same experimental condition, there was significant cell-to-cell variability in pulsing activity (Figs 2B and 3C). This observation could result from stable differences in pulsing behavior between cells. Alternatively, single cells could transition back and forth between pulsing and non-pulsing states, which would show up as different behaviors when observation times are limited in comparison with the characteristic times of such transitions. To identify changes in pulsing behavior of single cells over longer timescales, we recorded movies for 18 h such that cells could be followed from birth to division (Fig. 4A; Fig. S13). Increasing the frame intervals to 105 s reduced overall light exposure, while still allowing to resolve pulses that are at least 5.25 min apart. We recorded pulsing in wild-type cells growing in N2B27 medium, thereby exclusively focusing on pulsing driven by paracrine FGF4 signaling, and avoiding possible ligand depletion that could occur with exogenous FGF4. The KTR signal increased over long timescales (Fig. S13), reflecting an overall decrease in KTR fluorescence levels in these long recordings (Fig. 4B). We therefore first performed baseline correction to filter out these effects, and then established an alternative peak-finding approach to quantify and annotate these low temporal resolution traces (Fig. 4C; Fig. S14; Materials and Methods). The IPI distribution in this dataset was consistent with those obtained with higher time resolution (Fig. S15, compare with Figs 2F and 3F).

We made raster plots showing occurrence of pulses in cells that we could follow from immediately after cell division (Fig. 4D). Visual inspection of these raster plots suggested that pulses were concentrated towards the beginning of the cell cycle. A change in pulsing activity over time could be a consequence of cell cycle effects on pulsing, or it could result from non-stationary experimental conditions.

To visualize the contributions from these two possible causes, we introduced a two-dimensional time map. The coordinates in this map are experimental time *T*_*e*_, which is time measured from the beginning of the time lapse movie, and *T*_*b*_, the time relative to individual cell birth. For each cell *i*, the trace begins at 
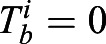
 and experimental time 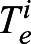 is the time when cell *i* was born measured from the beginning of the movie. From this point in the map, individual traces would fall along a diagonal line of unit slope. To reveal the population behavior and avoid superposition of individual traces in the map, we plotted pulse rate averaged in 70 min bins along both axes. In each bin, we counted the total number of pulses from all traces in that bin and divided by the total number of minutes of recording that contribute to that bin. On this pulse rate map, cell cycle effects would manifest as a rate change in the horizontal direction (Fig. 4E, upper panel), whereas non-stationary experimental conditions would manifest as a change in rate in the vertical direction (Fig. 4E, lower panel).

Inspection of the pulse rate time map revealed a higher pulse rate at the bottom left of the plot that decreased both towards the right and the top (Fig. 4F). This behavior indicates that pulse rate decays across the cell cycle, in addition to effects of non-stationary experimental conditions. To quantify this observation, we further binned pulse rate at larger timescales (Fig. 4G). In this coarse-grained map, pulse rate within the same experimental time window was consistently higher in cell populations which were earlier in their cell cycles. We obtained similar results when applying an alternative detrending strategy (Figs S14 and S16), as well as when analyzing cells growing in serum+LIF medium (Fig. S17). Taken together, these results confirm that cells are more prone to pulse earlier in their cell cycle.

### Intermittent ERK oscillations are less prevalent in more differentiated cells

**Fig. 5.**
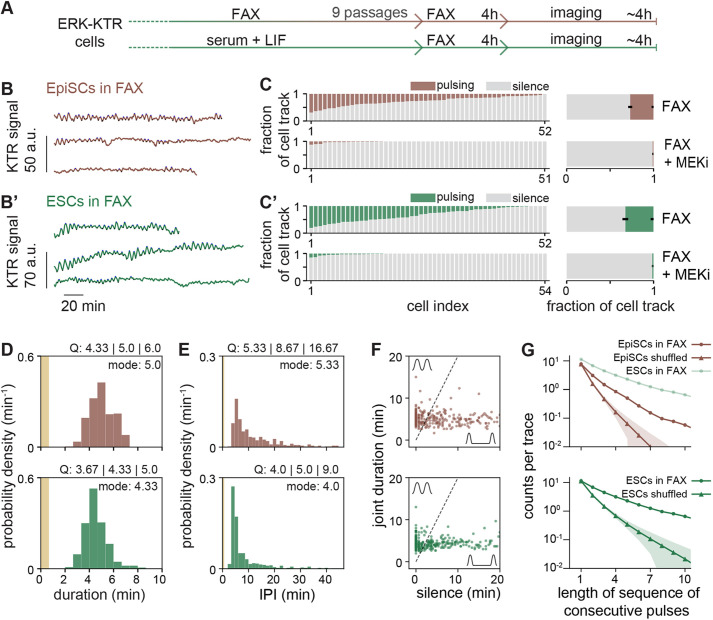
**Intermittent ERK oscillations are less prevalent in more differentiated cells.** (A) Schematic of experimental approach to compare dynamical signatures of ERK pulsing in ESC and EpiSCs growing in the same medium. (B,B′) Smoothened single cell traces of KTR signal in EpiSCs (brown, B) and ESCs (green, B′) growing in FAX medium. Pulses are indicated by the maxima (blue dots) and corresponding minima (black dots) that define them. (C,C′) Left: fraction of time that individual EpiSCs (C) or ESCs (C′) spent pulsing (brown/green) or non-pulsing (gray) in FAX alone (top) or upon addition of MEKi (bottom). Right: average time that cells were pulsing (brown/green) or non-pulsing (gray) in the cell population. Error bar indicates s.e.m. (D) Pulse duration distribution for EpiSCs (top, brown, *n*=402 pulses) and ESCs (bottom, green, *n*=588 pulses). (E) Interpulse interval (IPI) distribution for EpiSCs (top, brown, *n*=351 pairs of pulses) and ESCs (bottom, green, *n*=540 pairs of pulses). Pulse recognition resolution limit (yellow bar) and quartiles (Q) 25, 50 and 75 are indicated in D,E. (F) Joint pulse duration versus silence intervals for successive pairs of pulses in EpiSCs (top, brown) and ESCs (bottom, green). The slope 2 dashed line classifies pairs of pulses into consecutive (above) and non-consecutive (below). The axes range was adjusted to better resolve individual data points, leaving off the scale 45 out of 351 data points for EpiSCs, and 36 out of 540 data points for ESCs. (G) Number of pulse trains as a function of the number of consecutive pulses in the train. Counts have been normalized by the number of traces. Top: EpiSCs data (brown dots), ESCs data (green dots) and heterogeneous population model for EpiSCs data (brown triangles). Bottom: ESCs data (green dots, same as top) and heterogeneous population model for ESCs data (green triangles). Shaded area is the s.d. from 200 independent realizations of the stochastic models. Data in D-G from *n*=52 cells in FAX alone for each cell type.

Finally, we asked whether the dynamical signatures of ERK pulsing were preserved in more differentiated cells. We transitioned reporter cells from an ESC to an epiblast stem cell (EpiSC) state by culturing them for at least nine passages in N2B27 supplemented with FGF2 and activin (Guo et al., 2009). To obtain more homogeneous cultures, we added the Wnt inhibitor XAV939 (FAX medium; Sumi et al., 2013). We then recorded ERK dynamics in EpiSCs growing in FAX medium with or without MEKi. Any changes in the ERK dynamics under these conditions compared with ESCs growing in serum+LIF medium could be due to the different media, or be a consequence of differentiation status. To distinguish between these possibilities, we compared ERK dynamics in EpiSCs with those in ESCs transferred to FAX medium shortly before the beginning of the recording (Fig. 5A). In both conditions we could observe ERK pulsing, which was abrogated by addition of MEKi (Fig. 5B; Movie 3; Figs S18 and S19). Although ERK pulsing was similarly heterogeneous across the population in both conditions, the fraction of time that cells were pulsing was lower for EpiSCs than for ESCs (Fig. 5C). The duration of pulses was slightly longer in EpiSCs compared with ESCs (Fig. 5D). Together with the shorter fraction of time spent pulsing, this indicates that pulses are less frequent in EpiSCs compared with ESCs. Analysis of the IPI distributions revealed stronger differences between the two conditions. Although the mode of the IPI distribution in EpiSCs was 5.33 min, it was only 4 min in ESCs, indicating faster pulsing. IPIs were also more narrowly distributed in ESCs in FAX compared with EpiSCs (Fig. 5E), suggesting that pulsing was more regular in ESCs. This was confirmed by plots of joint pulse duration against silence interval duration (Fig. 5F), which indicated that 65.6% of all pairs of pulses were consecutive in ESCs, in contrast to only 45.9% in EpiSCs. Longer sequences of consecutive pulses were more prevalent in ESCs compared with EpiSCs (Fig. 5G, top). For both EpiSCs and ESCs, longer sequences of consecutive pulses were also more abundant in the data compared with a heterogeneous population model, in which pulses were randomly shuffled on individual traces (Fig. 5G).

In summary, more differentiated EpiSCs pulse for a shorter amount of time, with longer pulse durations compared with ESCs. Furthermore, consecutive pulses as well as longer pulse sequences are less prevalent than in ESCs. Thus, the dynamical signatures of ERK pulsing depend on differentiation state. Still, ERK dynamics in both EpiSCs and ESCs are inconsistent with simple stochastic pulsing scenarios. This suggests that ERK signaling in both cell types may operate in a similar dynamic regime.

## DISCUSSION

Here, we report fast pulses of ERK activity in mouse ESCs under a continuous stimulation regime. We detect long trains of consecutive pulses that are inconsistent with simple stochastic pulsing scenarios. Instead, we propose that these data can be interpreted as intermittent oscillations, with transitions between silent and oscillatory states in single cells. Oscillations are triggered by FGF4. Across a range of FGF4 ligand concentrations, we find oscillations with similar individual pulse durations. With increasing FGF4 concentrations, the distribution of IPIs becomes narrower and the extent of consecutive pulsing increases, suggesting more regular oscillations.

The detection of signal-dependent ERK activity dynamics on short time scales in ESCs was made possible by combining the KTR sensor with high time-resolution recordings. A previous study, which examined ERK dynamics upon acute stimulation, focused on long-term activity and did not resolve the short-timescale oscillations that we report here (Deathridge et al., 2019). These previously undetected dynamics have a modal IPI of ∼7 min (that is, about 8 pulses/h), and are thus much faster than in any other cell system described so far.

Both paracrine and recombinant FGF4 stimulation of ESCs trigger oscillatory ERK activity with similar timescales of pulse duration and IPI, indicating that oscillations emerge in the intracellular signal transduction network, similar to the situation in other cell lines (Sparta et al., 2015). The short frequencies of ERK oscillations in ESCs further support the notion that they are driven by short-timescale delayed feedbacks such as post-translational modifications at the receptor level (Sparta et al., 2015), or at various levels within the MAPK cascade (Lake et al., 2016; Lemmon et al., 2016). Notably, we found that ERK pulses were faster when ESCs were growing in FAX instead of in serum+LIF (compare Fig. 5 with Fig. 2). This suggests that media components can affect the dynamical signatures or ERK activity.

Pulsatile ERK activity in single cells upon continuous stimulation of receptor tyrosine kinases has been reported in many cell types (Albeck et al., 2013; Goglia et al., 2020; Shankaran et al., 2009), indicating that the tendency to generate time-varying ERK activity patterns is a general feature of receptor tyrosine kinase signal transduction. In addition to the timescales, the dynamic signatures of FGF-triggered ERK pulses in ESCs differ markedly from those observed in most other contexts.

ERK pulses in ESCs have well-defined durations and often occur in consecutive sequences, consistent with oscillations. This is in contrast to the more irregular stochastic pulsing reported in several immortalized cell lines and keratinocytes (Albeck et al., 2013; Aoki et al., 2013; Goglia et al., 2020). In cell systems that show stochastic ERK pulsing, increasing ligand levels leads to shorter IPIs and hence to an increase in pulse rate (Albeck et al., 2013). This has been interpreted as encoding of ligand concentration through frequency modulation (Li and Elowitz, 2019). In ESCs, simple stochastic pulsing models fail to capture the statistics of isolated and consecutive pulses observed in experiments. Thus, although pulse rate may convey information about ligand levels in differentiating ESCs, the frequency-modulated encoding model proposed for stochastic ERK pulsing is unlikely to apply.

The oscillatory ERK activity that we detect here indicates the presence of negative feedback mechanisms downstream of FGF signaling in ESCs (Novák and Tyson, 2008). Negative feedback in the RAF-MEK-ERK cascade sets a ligand dose response range, and linearizes signal transduction despite non-linear signal amplification (Sturm et al., 2010). In ESCs and cells of the early embryo, FGF4 concentration smoothly tunes the proportion of differentiated cell types (Krawchuk et al., 2013; Raina et al., 2021). Thus, ERK oscillations in ESCs might be the consequence of negative feedback mechanisms that have evolved to tune the response range of the signal transduction system to the physiologically relevant range of paracrine FGF4 concentration. Identifying the relevant feedback circuits may require combining temporal stimulation with perturbations of candidate mechanisms and single-cell dynamic read-outs (Blum et al., 2019).

Regular oscillations of ERK nuclear import and export have been reported upon EGF stimulation in mammary epithelial cells (Shankaran et al., 2009). In these cells, the frequency of ERK oscillations is insensitive to ligand levels over a wide range (Shankaran et al., 2009), similar to what we find upon titrating FGF4 in ESCs. However, ESC populations contain a mixture of oscillating and non-oscillating cells as well as cells that transition between these regimes across a wide range of ligand levels. We interpret this behavior to indicate that the FGF/ERK signal transduction system in ESCs is organized in the vicinity of a transition point between a non-oscillatory and oscillatory state. In this framework, increasing FGF4 levels would bring the system closer to this point. Similarly, the decay of ERK pulsing across the cell cycle can be interpreted as cells shifting away from the oscillatory to a non-oscillatory state, possibly through changes in the surface-to-volume ratio or cell cycle-dependent expression of components of the FGF/ERK signaling system. A positioning close to a transition between oscillatory and stationary behavior has been described in hair cells of the cochlea (Camalet et al., 2000; Eguíluz et al., 2000), the actin system of *Dictyostelium* (Westendorf et al., 2013), and isolated cells of the growing vertebrate body axis (Webb et al., 2016). In such a scenario, isolated pulses could result from short excursions from the stationary into the oscillatory regime. Alternatively, the non-oscillatory state could be a distinct dynamic regime producing isolated pulses (Martinez-Corral et al., 2018; Mönke et al., 2017). The nature of the non-oscillatory state is still unknown in ESCs, together with the type of transition.

The intermittent oscillations generated by the cell-type specific organization of the FGF/ERK signaling system introduce a source of cellular heterogeneity that may be relevant for cell fate decisions in differentiating pluripotent cells. In the embryo, FGF/ERK signaling regulates the differentiation of both primitive endoderm as well as epiblast cells. Different dynamic signaling activities may underlie the establishment of these two discrete lineages from a common precursor cell type in response to the same signal (Pokrass et al., 2020). In ESC cultures, the heterogeneous signaling dynamics that we describe add another dimension to the transcriptional heterogeneities that prefigure cell differentiation (Canham et al., 2010; Chambers et al., 2007; Hayashi et al., 2008; Singh et al., 2007; Toyooka et al., 2008). Although signaling is not necessarily off when ERK is not pulsing, intermittent ERK oscillations in ESCs are reminiscent of the transcriptional dynamics of many genes, for which expression bursts and silent periods alternate (Tunnacliffe and Chubb, 2020). Correlating signaling dynamics with developmental transcriptional programs will be required to discern how these two levels are interlinked, and how they relate to cell differentiation.

## MATERIALS AND METHODS

### Cell culture

mESCs were routinely cultured on 0.1% gelatin (Sigma-Aldrich)-coated tissue culture flasks in serum+LIF medium composed of GMEM (Thermo Fisher Scientific), 10% batch-tested fetal bovine serum (FBS) (Sigma-Aldrich), 1× GlutaMAX (Thermo Fisher Scientific), 1 mM sodium pyruvate (Thermo Fisher Scientific), 1× non-essential amino acids solution (Thermo Fisher Scientific), 100 µM 2-mercaptoethanol (Thermo Fisher Scientific) and 10 ng/ml LIF (Max Planck Institute protein expression facility). Cells were passaged every 2-3 days using 0.05% Trypsin (PAN Biotech). Basal medium for serum free culture was N2B27, prepared as a 1:1 mixture of DMEM/F12 (PAN Biotech) and Neuropan basal medium (PAN Biotech) with 0.5% bovine serum albumin (BSA), 1× N2 and 1× B27 supplements (Thermo Fisher Scientific) and 50 µM 2-mercaptoethanol. For FGF stimulation experiments, short-term serum-free culture was carried out in N2B27 supplemented with 3 µM CHIR99201 (Tocris), 1 µg/ml of Heparin (Sigma-Aldrich) and with or without 10 ng/ml LIF as indicated. Recombinant human FGF4 used was obtained from Peprotech. Transitioning of cells to an EpiSC state was achieved by culturing for at least nine passages in FAX medium, consisting of N2B27 basal medium supplemented with 12 ng/ml FGF2 (Cell Guidance Systems), 25 ng/ml ActivinA (Peprotech) and 20 µM XAV939 (Sigma-Aldrich). For live imaging and immunostaining studies, cells were seeded on polymer-bottomed ibidi µ-slides (ibidi) coated with 20 µg/ml fibronectin.

### Cell lines

All KTR-expressing cell lines used in this study were derived from E14tg2a (Hooper et al., 1987). Targeting of the ERK-KTR-Clover construct into the *Hprt* locus has been described elsewhere (Simon et al., 2020). Mutagenesis of the *Fgf4* gene was performed by co-transfection of a CRISPR-construct and a repair template introducing a nonsense and a frameshift mutation as previously described (Morgani et al., 2018). Clones with the desired mutation were identified by restriction digest and Sanger sequencing of a PCR fragment encompassing the *Fgf4* start codon. Clonal cell lines were tested for their chromosome count using standard procedures (Nagy et al., 2008) and only cell lines with a modal count of *n*=40 were used for analysis. *Fgf4^−/−^, Spry4^H2B-Venus/+^* cells used to evaluate transcriptional activation downstream of recombinant FGF4 have been previously described (Morgani et al., 2018). All cell lines were regularly tested for mycoplasma contamination.

### Dual reporter experiments

The ERK-KTR-mCherry construct for transient expression was prepared by first inserting the coding sequence for ERK-KTR (Regot et al., 2014) into a CMV-driven mCitrine C1 expression vector (TaKaRa), and then replacing the fluorophore for mCherry. The plasmid for transient expression of EKAREV-NLS has been previously described (Komatsu et al., 2011). The two plasmids were transiently co-transfected into E14tg2A mouse ESCs using Lipofectamine 2000 (Thermo Fisher Scientific) in suspension according to the manufacturer's instructions. Cells were plated on fibronectin-coated ibidi slides and imaged 24 h after transfection.

### Western blotting

Cells were grown to confluency on fibronectin-coated tissue culture dishes and exposed to indicated experimental conditions. Cells were briefly washed twice with ice-cold PBS supplemented with 1 mM activated sodium orthovanadate and then lysed using commercially available lysis buffer (Cell Signaling Technology) supplemented with benzonase (Thermo Fisher Scientific), phosphatase inhibitor cocktail 2 and 3 (Sigma-Aldrich), and cOmplete EDTA-free protease inhibitor cocktail (Roche). Lysates were snap-frozen in liquid nitrogen. Protein concentration was estimated using a micro-BCA assay (Thermo Fisher Scientific), and lysates were denatured by adding appropriate amounts of 5× Laemmli buffer and boiling for 5 min. Then 10 or 20 μg protein was loaded across all wells in any given gel. Bis-Tris SDS gels were run with 1× MOPS buffer (Thermo Fisher Scientific) with fresh sodium bisulfite, and subsequently transferred onto methanol-activated PVDF membranes (Millipore) at 40 V for 1.5 h with the NuPage transfer system (Thermo Fisher Scientific). Primary antibodies used were anti-Tubulin (1:5000, T6074, Sigma-Aldrich), anti-pERK1/2 (1:1000, 4370S, Cell Signaling Technology), and anti-total ERK1/2 (1:1000, ab36991, Abcam) along with appropriate IRDye-labeled secondary antibodies (LI-COR) at 1:10.000 dilution. Bands were detected using the Odyssey CLx imaging system (LI-COR). Bands were quantified using FIJI/ImageJ (Rueden et al., 2017). For quantification of pERK and total ERK, integrated intensity in both ERK1 and ERK2 bands was added.

### Immunostaining

For pERK immunostaining, cells were fixed for 15 min at 37°C by diluting fixative stocks directly into cell culture medium to a final concentration of 4% paraformaldehyde (PFA) and 0.01% glutaraldehyde (Sigma-Aldrich). After a brief wash with PBS, cells were permeabilized with 100% methanol at −20°C. For all other antibodies, fixation was performed with 4% PFA at room temperature for 20 min. Cells were washed with PBS and then simultaneously blocked and permeabilized with 5% normal goat serum (Thermo Fisher Scientific) in 0.5% Triton X-100 (Serva) in PBS for 60 min. Antibody staining was carried out overnight at 4°C in PBS+0.1% Triton X-100 and 1% BSA (Sigma-Aldrich). Primary antibodies used were anti-pERK1/2 (1:200, 4370S, Cell Signaling Technology), anti-E-Cad (1:200, M108, clone ECCD-2, TaKaRa), anti-Nanog (1:200, eBIO-MLC51, eBioscience), anti-POU5F1 (1:200, C-10, sc-5279, Santa Cruz Biotechnology), along with AlexaFluor-labeled secondary antibodies (Invitrogen) at 1:500 dilution. Hoechst 33342 was used at 1 µg/ml to counter-stain nuclei, and CellMaskRed (Thermo Fisher Scientific) was used to label membranes according to the manufacturer's instructions. After staining, samples were covered with 200 µl of antifade composed of 80% w/v glycerol with 4% w/v N-propyl gallate and stored at 4°C. Images were analyzed using custom scripts in MATLAB (The Mathworks) and Fiji/ImageJ for the detection of nuclei as well as an active-contours-based identification of membranes.

### Flow cytometry

Cells were grown on fibronectin-coated dishes in N2B27 supplemented with 3 µM CHIR99201, 1 µM PD0325901 and 10 ng/ml LIF (2i+LIF) for 3 days. For stimulation, cells were washed 2× with PBS, and FGF4 was added at indicated concentrations in serum-free N2B27 medium supplemented with 3 µM CHIR99201 and 1 µg/ml Heparin for 24 h. Cells were then trypsinized and fixation was performed in suspension with 4% PFA at room temperature for 15 min. After a brief wash in PBS, cells were resuspended in PBS+1% BSA and analyzed on a BD-LSR II (BD Biosciences) flow cytometer. Data was analyzed in FlowJo (BD Biosciences).

### Live cell imaging and cell tracking

ERK-KTR expressing cells were cultured on ibidi µ-slides, and imaged on a Leica SP8 confocal microscope equipped with an incubation chamber and CO_2_ supply to maintain temperature at 37°C, CO_2_ at 5%, and relative humidity at 80%. Live-cell nuclear dye SiR-Hoechst 652/674 (Spirochrome) was added 4 h before acquisition to facilitate tracking of cells. SiR-Hoechst was added at a final concentration of 500 nM for short-term time-lapse experiments, and 250 nM for long-term time-lapse experiments. Fluorophores were excited with a 504 nm line from a white-light laser (Leica), and images of the KTR-Clover and the nuclear marker were simultaneously captured through a 63×1.4 N.A. oil objective. For short-term (<4 h) imaging experiments, single frames were acquired once every 20 s, with an *xy* resolution of 0.251 µm, a pixel dwell time of 2.6 μs and a pinhole of 2.4 airy units. For long-term (∼19 h) imaging experiments, to minimize overall light exposure, single frames were acquired once every 105 s, with an *xy* resolution of 0.401 µm, a pixel dwell time of 3.1 μs and a pinhole of 2.6 airy units. Images were processed with custom MATLAB scripts to enhance contrast and highlight nuclei to facilitate automatic tracking.

To determine the nuclear and cytoplasmic fluorescence intensities shown in Fig. S2, we manually drew regions of interest (ROIs) in every fifth frame of the movies. For the nuclear ROI we aimed at capturing the entire nuclear area. For cytoplasmic ROIs we selected contiguous regions that could be unambiguously assigned to a specific cell. We then used the interpolation function of FIJI to take measurements in each frame. All other cell tracking was performed using the Trackmate plugin (Tinevez et al., 2017) for FIJI/ImageJ. Tracking was initially performed automatically for the entire colony, and tracks were subsequently manually curated frame-by-frame by removing any cells that did not display a typical ESC morphology with a small cytoplasm and round, well-defined nuclei. We also removed cells that left the field of view, and adjusted tracking in individual frames for incorrectly identified nuclei. We inverted fluorescence values to obtain the negative image, and then measured mean fluorescence intensities in an ROI of variable size within each tracked nucleus. In these KTR signal traces, low intensity values correspond to low ERK activity and high intensity values indicate high ERK activity. For the short-term imaging, tracks started at the beginning of the movie and extended until the end of the movie, or until cell division. As the long-term imaging experiments were designed to capture the entire cell cycle, tracks started in the first frame following cell division where a cell could be tracked, and ended at cell division. In these experiments, we kept tracks of cells that left the field of view, but only if they were observed for longer than 4.5 h.

### Time series preprocessing

We screened and corrected time series for tracking errors, such as ROIs placed partially outside the nucleus or overlapping with a nucleolus. Because these structures have fluorescence intensities that usually differ from that of the nucleoplasm, these tracking errors usually led to an increase in the variance of the pixel intensity across the ROI. We screened time series for high variance regions, checked the tracking for all instances where the variance crossed a manually set threshold value, and corrected the tracking if this was required.

Just before cell division, the sensor was excluded from the nucleus, resulting in a pulse of the KTR signal at the end of dividing cells tracks (for example cells 30, 31, 41 and 50 in the serum+LIF condition without MEKi, Fig. S4). As this pulse of reporter exclusion was insensitive to MEK inhibition, it is unlikely to be reporting ERK activity and we therefore decided to trim these events from all traces. Although most cells divided in the long-term measurements, only a few did it in short-term measurements. Correspondingly, in short-term measurements we deleted the last 20 frames (∼7 min) of the time series of dividing cells only. In the long-term measurements, during which most cells divided, we discarded the last 15 frames (26.25 min) of each time series.

### Analysis of ERK dynamics in short-term high resolution datasets

#### Pulse recognition

We defined a pulse as a local maximum between two local minima, imposing two conditions: (1) we required amplitude to be larger than a threshold amplitude *A*_*th*_; (2) slope to be larger than a threshold slope *v*_*th*_. The amplitude and slope thresholds are free parameters of the algorithm. These free parameters were set through a quantitative threshold analysis protocol described below and were specific for each dataset (Table S1; Figs S5, S11 and S19).

To remove high frequency noise that interfered with the performance of the pulse detection algorithm, we first smoothed the time series. We filtered the highest frequencies in the data using a moving average window of three frames of duration. That is, for each KTR signal value *x*_*i*_ of the time series, we computed the average value

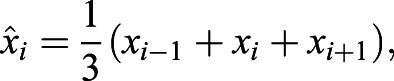
where *i* is the frame number. At the boundaries we considered average windows of two and one frames. Note that detrending was not required in the case of this data.

We first searched the time series for all the local maxima and minima. We compared each value 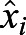 of the time series with its immediate neighbors 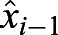 and 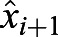. The initial value 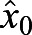 was compared only with the next value 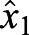, and the last value 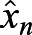 with the previous one 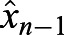. We discarded the first maximum if there was no minimum on its left, and the last maximum if there was no minimum on its right. In this way we defined a subset of data points consisting of the maxima 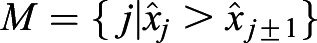, and the subset of minima 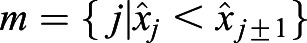. From the definition, it follows that the minimum distance |*i*−*j*| between two maxima 
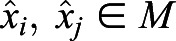
 is two frames, and the minimum distance |*k*−*l*| between two minima 
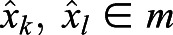
 is two frames.

To identify pulses from this set of maxima we applied two filters, one for pulse amplitude and another one for pulse slope. To implement the pulse amplitude and pulse slope filters, we considered each maximum of the time series, from left to right. For each maximum *j* ∈ *M* of 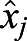, we searched for the first minimum to its left *k* ∈ *m* such that the resulting left amplitude 
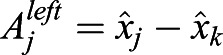
 was larger than the amplitude threshold 
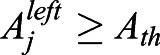
 and the left slope was larger than the slope threshold 
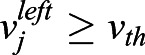
 (see threshold analysis protocol below). The left slope was defined as 
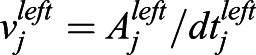
, where 
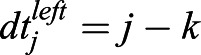
 is the left pulse duration. Similarly, we searched the first minimum to the right that verified 
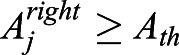
 and 
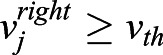
. We next removed overlapping pulse candidates: if the right minimum of the first pulse occurred later than this new left minimum of the second one, we discarded the pulse that had the smaller amplitude 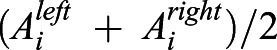.

#### Threshold analysis protocol

Pulse recognition depends on the free parameters for amplitude threshold *A*_*th*_ and slope threshold *v*_*th*_. To rationally set values for these two threshold parameters, we first focused on the negative control condition for each respective experiment, where ERK pulsing was minimal. We determined parameter combinations for which a fixed, low number of pulses was detected in the negative control, and then selected specific parameter values that maximized the number of pulses recognized in the experimental condition in which ERK was most active (Table S1; Figs S5, S11 and S19).

We started by establishing a two-dimensional exploratory parameter space for each dataset (Table S1). For each combination of parameters 
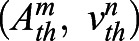
 on the exploratory parameter space, we ran the pulse detection algorithm described in the previous section for the negative control and computed the averaged pulse rate

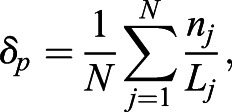
where *N* is the total number of cells in the negative control, *n*_*j*_ is the number of detected pulses for cell *j* and *L*_*j*_ is the length of the time series. We then introduced exploratory level curves across the parameter space by fixing average pulse rate values 
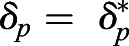
 in the negative control (Table S1). This restricted parameter combinations to curves in the exploratory parameter space. Next, for each 
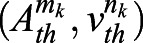
 combination on each exploratory level curve *k*, we applied the pulse recognition algorithm on the experimental condition where ERK was most active. The plot of pulse rate along this level curve showed a flat region of similarly high pulse detection. Within this region, we chose parameter pairs that filtered out spurious pulses that were flat and long from the negative control. This resulted in a pair of parameters (*A*_*th*_, *v*_*th*_) specific for each experiment (Table S1).

#### Quantitative pulse dynamics characterization

To characterize dynamical activity of the time series, we introduced a set of quantitative measures (Fig. 2D). For each pulse *P*_*i*_ in the set of pulses *P*={*P*_*j*_=(*j*, *k*_*j*_, *l*_*j*_) | *P*_*j*_ *is a pulse*} we defined the pulse amplitude *A*_*i*_ as the average of its right and left amplitudes

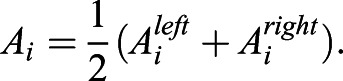
Pulse duration *dt*_*i*_ was defined as the distance between the two minima that define the pulse

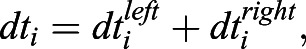
and the joint pulse duration *dt*_*i*,*j*_ between a pair of successive pulses *P*_*i*_, *P*_*j*_ with *j* > *i*, as the sum of the right pulse duration of the earlier pulse *i* and the left pulse duration of the later pulse *j*

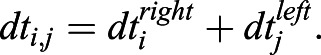
We computed the interpulse interval *IPI*_*i*,*j*_ between a pair of successive pulses *P*_*i*_, *P*_*j*_ with *j* > *i*, as the time interval between their maxima

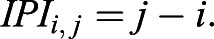
The silent interval *dm*_*i*,*j*_ between a pair of successive pulses *P*_*i*_, *P*_*j*_ was defined as the time elapsed between the right minimum of the earlier pulse *P*_*i*_ and the left minimum of the later pulse *P*_*j*_, that is

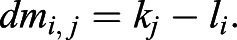
Note that calculating these last three quantities requires a trace with at least two pulses. These quantities satisfy the relationship

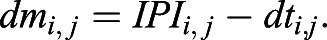
The values that these quantitative measures can take are constrained by the resolution imposed by pulse recognition. The minimum distance |*i*−*j*| between two maxima 
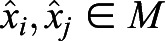
 was previously set to two frames. Thus, the distance between maxima of pulses *P*_*k*_, *P*_*l*_ ∈ *P* verifies |*k*−*l*| ≥ 2 frames, and in particular *IPI*_*k*,*l*_ ≥ 2 frames for any pair of consecutive pulses *P*_*k*_, *P*_*l*_ ∈ *P*. Similarly, the minimum distance |*i*−*j*| between two minima 
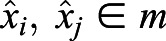
 is two frames. Consequently, given a pulse *P*_*j*_ = (*j*, *k*_*j*_, *l*_*j*_) ∈ *P*, the distance between the two minima that defines the pulse *dm*_*j*_ = *k*_*j*_−*l*_*j*_ satisfies *dm*_*j*_ ≥ 2 frames. Finally, from the previous section we have the constraints *A*_*i*_ > *A*_*th*_ and *v*_*i*_ > *v*_*th*_.

We classified pulses as consecutive or isolated. Inspection of the raw data indicated that pulse duration was more variable between cells in the same condition than within a cell. For this reason, we made the criterion for consecutiveness dependent on joint duration of the half-pulses that flank an intervening silent period. Specifically, we established that a pair of successive pulses *P*_*i*_, *P*_*j*_ are consecutive pulses if the silent interval between them *dm*_*i*,*j*_ is shorter than half of their joint duration *dt*_*i*,*j*_, that is *P*_*i*_, *P*_*j*_ are consecutive if *dm*_*i*,*j*_≤0.5 *dt*_*i*,*j*_. Pulses that do not belong to a consecutive pair are isolated pulses.

We also introduced a quantitative measure to characterize the dynamical activity on a population level. Given a single cell *c* associated to a time series of total length *T* and *n* pulses, the pulsing measure *A*_*c*_ is defined as the proportion of time that a single cell is pulsing

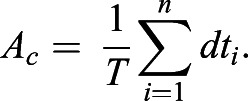


#### Kolmogorov–Smirnov test and notation

We implemented the Kolmogorov–Smirnov two-sample test (Frodesen et al., 1979) available on the ‘stats’ module of the SciPy package from Python (Virtanen et al., 2020). The aggregated data for all quantities considered is summarized in Table S2.

#### Time series autocorrelation

We first subtracted the mean value from smoothed traces *X*(*t*),

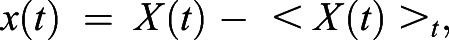
where < … >_*t*_ is the mean value taken over *t*. We then computed the autocorrelation function,

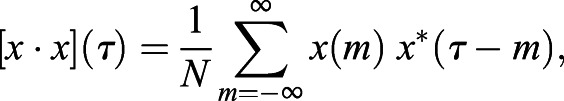
where x*(*t*) is the complex conjugate of *x*(*t*), *τ* is a time-lag, and *N* the normalization constant

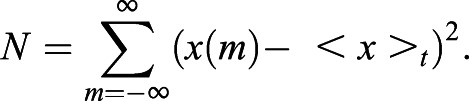
The time series *x*(*t*) was zero-padded when necessary. We computed this autocorrelation function using NumPy (Harris et al., 2020).

### Stochastic pulsing analysis

#### Homogeneous population model

We sought to compare the experimentally observed pulsing dynamics with simple stochastic models. We first considered a Poisson process, a kind of stochastic process which describes independent events that occur at a rate *λ*>0. In a Poisson process, waiting times *T* between events are distributed according to

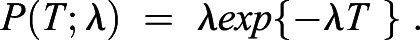
Note that this waiting times distribution is fully characterized by a single parameter *λ*. To obtain the value of *λ*, we first computed the distribution of times between pulses from experimental data (Fig. S8A). To obtain a more precise estimate of *λ*, we fitted the linear function

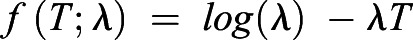
to the log of the experimental distribution using the least squares fit from NumPy.

We next generated traces from a Poissonian stochastic process with this estimated rate. We first obtained a discrete waiting times distribution sampling as many data points as there were pulses (Fig. S8B).

We then generated as many time series as we had cells in the experiment. The length of each generated trace corresponded to the length of an experimental trace. The waiting time to the beginning of the next pulse was sampled from the exponential distribution, and pulse duration was a random sample from the experimental pulse duration distribution. We represented pulses as squares and annotated their peaks in the middle (Fig. S8E).

We then analyzed these stochastic time series following the same analysis protocol that we used for the experimental data. Pulses that extended beyond the end of the trace were not included in the analysis.

#### Heterogeneous population model

We sought to generate stochastic pulsing traces such that each has the same total length and pulse rate as a corresponding experimental trace. Pulse duration was established as the mean duration of pulses in the experimental trace. We placed the first pulse in a random position of an empty trace. The time interval occupied by this pulse was then forbidden for the rest of the pulses we had to place. We repeated the procedure with the second pulse. If the pulse started or ended within the forbidden interval we considered this a failed iteration. Similarly, we excluded the iteration if the pulse ended after the time series. Upon a failed iteration, we tried again to place the pulse until success, stopping after 10^7^ unsuccessful iterations. We repeated this procedure with all the pulses on the time series. Note that, with this procedure, the number of pulses in the shuffled trace will always be bounded by the total number of pulses in the experimental trace. We represented pulses as squares and annotated their peaks in the middle (Fig. S8F). We then analyzed these stochastic time series following the same analysis protocol that we used for the experimental data.

#### Calculation of pulse trains

We wanted to count how often sequences with a given number of consecutive pulses occurred in the data. For each trace, we first listed the pairs of successive pulses and assigned them labels, with a value of 1 if a pair of pulses was classified as consecutive, and 0 otherwise. The number of trains with two consecutive pulses was obtained as the sum of all labels in this list. To compute the number of trains with three consecutive pulses, we first generated a new list defined as the product of adjacent labels. In this list, each entry represents groups of three successive pulses, and has a value of 1 if the first and the second pulses, and the second and the third were previously classified as consecutive, and 0 otherwise. The number of trains with three consecutive pulses was obtained as the sum of all labels in this new list. We iterated this procedure to determine the number of trains with 4, 5, 6, … consecutive pulses: first, we generated a list by multiplying adjacent labels from the previous list, and then we summed up the labels of the resulting list.

### Analysis of ERK dynamics in long-term datasets

Long-term recordings to map ERK dynamics across the cell cycle were about 12.5 times longer and had a sampling rate reduced to about 1/5 compared with the short-term recordings (Fig. S13). These qualitative differences of these data prompted for a different analysis strategy. Due to this limited time resolution, we decided to exclusively focus on the occurrence and timing of ERK pulses in the long-term datasets, and hence refer to these features as peaks.

#### Peak detection

The long-term recordings data featured both low and high frequency fluctuations. Low frequency noise created variable trends that impeded direct comparison between traces, whereas high frequency noise could hinder the identification of activity pulses. We used two different filtering strategies to remove fluctuations: a baseline filtering that removed only low frequencies and a band-pass filter that removed both low and high frequencies. Both methods produced similar statistics after peak detection.

In the first strategy we flattened the baseline of each trace by subtracting a low degree polynomial that follows its minima (Fig. S14). To obtain this polynomial, we first identified all the local minima on each time series. We compared each value *x*_*i*_ of the time series with its two neighbors to the left *x*_*i*−1_ and *x*_*i*−2_, and to the right *x*_*i*+1_ and *x*_*i*+2_. The value *x*_1_ was compared with its two right neighbors and *x*_0_ to the left, and *x*_*n*−1_ with its two left neighbors and *x*_*n*_ to the right. We used the least squares method to fit a polynomial to the minima together with the endpoints of the trace (NumPy; Harris et al., 2020). Due to the variability in trace duration and baseline, we set a trace-specific polynomial degree, *deg*, to allow an accurate fit of the baseline while avoiding overfitting, with *deg*_*j*_ = (2+*m*_*j*_)/3, where *m*_*j*_ is the number of minima in trace *j*.

In the second strategy, we filtered the signal by removing unwanted high and low frequencies with a band-pass filter (Fig. S14). We applied a Butterworth filter with zero time and linear phase, by implementing the band-pass filter on a moving window both forward and backward in time (SciPy ‘signal’ submodule; Virtanen et al., 2020). We used an odd extension for the padded signal and a pad length of 15 frames, that is three times the number of coefficients of the Butterworth polynomials. The Butterworth filter is a band-pass square filter: it has a flat frequency response in the passband region, and rolls off towards zero in the stopband region. The order of the filter regulates the sharpness of the cutoff and we set it to four. We chose the cutoff frequencies *f*_*low*_ and *f*_*high*_ in terms of the maximum frequency we can resolve with the given sampling rate. We chose low and high stopband frequencies in terms of the Nyquist frequency, *f*_*low*_ = 0.025 *f*_*nyq*_ and *f*_*high*_ = 0.6 *f*_*nyq*_, with *f*_*nyq*_ = 0.5 *f*_*s*_ = (1/210) Hz for a sampling frequency *f*_*s*_ = (1/105) Hz.

We determined the local maxima by comparison of neighboring values. We compared each value 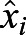 of the time series with its neighbors 
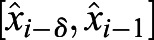
 and 
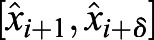
, where *δ* is a free parameter of the method that determines the minimum time interval between peaks that we could resolve. We reduced the range of comparison until reaching 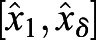 for the initial value 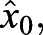 and 
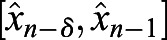
 for the final value 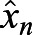. We set *δ* = 2 frames, which allowed us to resolve ERK-dependent peaks that are at least 5.25 min apart.

#### Threshold analysis protocol

To remove spurious low amplitude peaks, we filtered peaks with a KTR signal threshold value *I*_*th*_. We explored how the number of peaks changed in *Fgf4-*mutant cells in N2B27 (negative control) and wild-type cells growing in serum+LIF as we changed this threshold (Fig. S14). We detected peaks in the two conditions for different 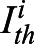 threshold values evenly spaced in the arbitrary unit (a.u.) range [0, 30]. For each 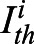, we computed the total pulse rate

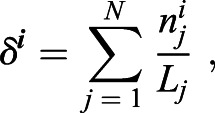
where *N* is the total number of cells of each condition, 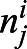 is the number of detected pulses for this threshold value 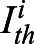 and *L*_*j*_ is the total length of the time series of cell *j*. We normalized pulse rate to the total pulse rate *δ*^0^ at 
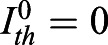
, 
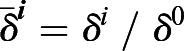
 (Fig. S14). This normalized pulse rate decreased with increasing the threshold values both in the negative control and the wild type. The negative control pulse rate decays much faster, reaching 0.5, whereas wild-type values are still around 0.9. Thus, wild-type genuine peaks can be distinguished from the background fluctuations in the control. We set a threshold value *I*_*th*_ for which 1% of all the local maxima were classified as peaks in the negative control, that is 
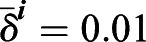
. This condition results in threshold values *I*_*th*_ = 24 for the frequency filtering strategy and *I*_*th*_ = 25 for baseline filtering strategy (Fig. S14).

#### Error estimation in pulse rate maps

Being 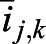 the contribution of vector 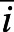 to (*T*_*b*,*j*_, *T*_*e*,*k*_), we interpreted each element of every 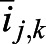 as an individual experiment with two possible outcomes: 1 (success) and 0 (failure). This *T* independent experiments in (*T*_*b*,*j*_, *T*_*e*,*k*_) had a characteristic probability of success *p* ∈ [0,1]. Then, the probability of obtaining 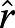 numbers of success in the *T* independent experiments in (*T*_*b*,*j*_, *T*_*e*,*k*_) is determined by the binomial distribution 
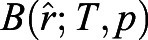
.

We were interested in estimating the relative number of successes in *T* trials 
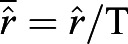
. Then, the maximum likelihood estimator for 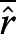 is given by 
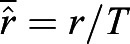
 and its variance 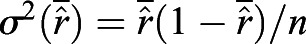 (Frodesen et al., 1979).

Understanding the *T* number of trials as a time interval, the maximum likelihood estimator of the relative number of successes is the previously defined pulse rate, i.e. number of peaks per unit time. Then, we estimated the pulse rate in each subspace (*T*_*b*,*j*_, *T*_*e*,*k*_) (Fig. 4G; Figs S16 and S17). The corresponding error was computed as the standard deviation. On this approach we assumed stationarity conditions for each subspace (*T*_*b*,*j*_, *T*_*e*,*k*_) by assuming a constant *p* in each case. We neglected small variations in *p* because we wanted to study the behavior of the previously characterized short-term dynamical activity (∼7 min) in long-term cell cycle time scales (∼13 h).

## Supplementary Material

Supplementary information

Reviewer comments
