# Supplementary material for: Intermittent ERK oscillations downstream of FGF in mouse embryonic stem cells
Source: Development. 2022 Feb 17;149(4):dev199710. doi: 10.1242/dev.199710 (PMC8918804; doi:10.1242/dev.199710)
Supplement: Supplementary information [file develop-149-199710-s1.pdf]

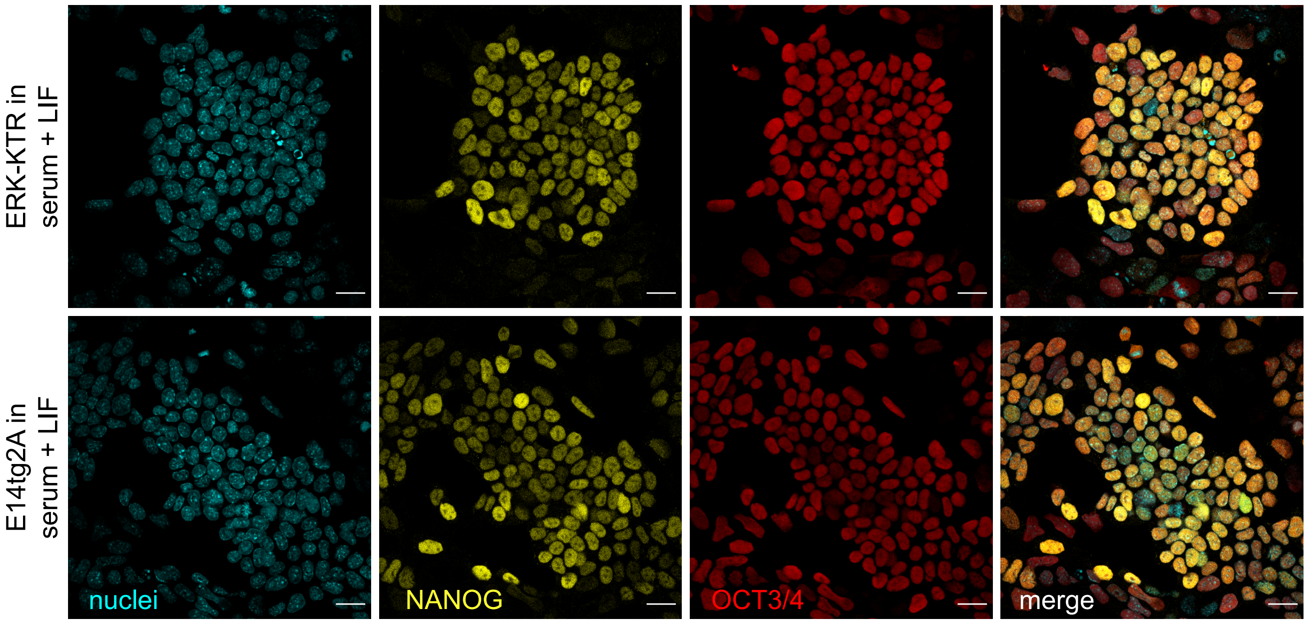

Fig. S1. Reporter cells and parental cells express similar levels of pluripotency markers. Immunostaining of ERK-KTR mESCs (top row) and the parental E14tg2a line (bottom row) for expression of pluripotency markers NANOG (yellow) and OCT3/4 (red). Nuclei in cyan. Scale bar: 20 µm.

Development • Supplementary information

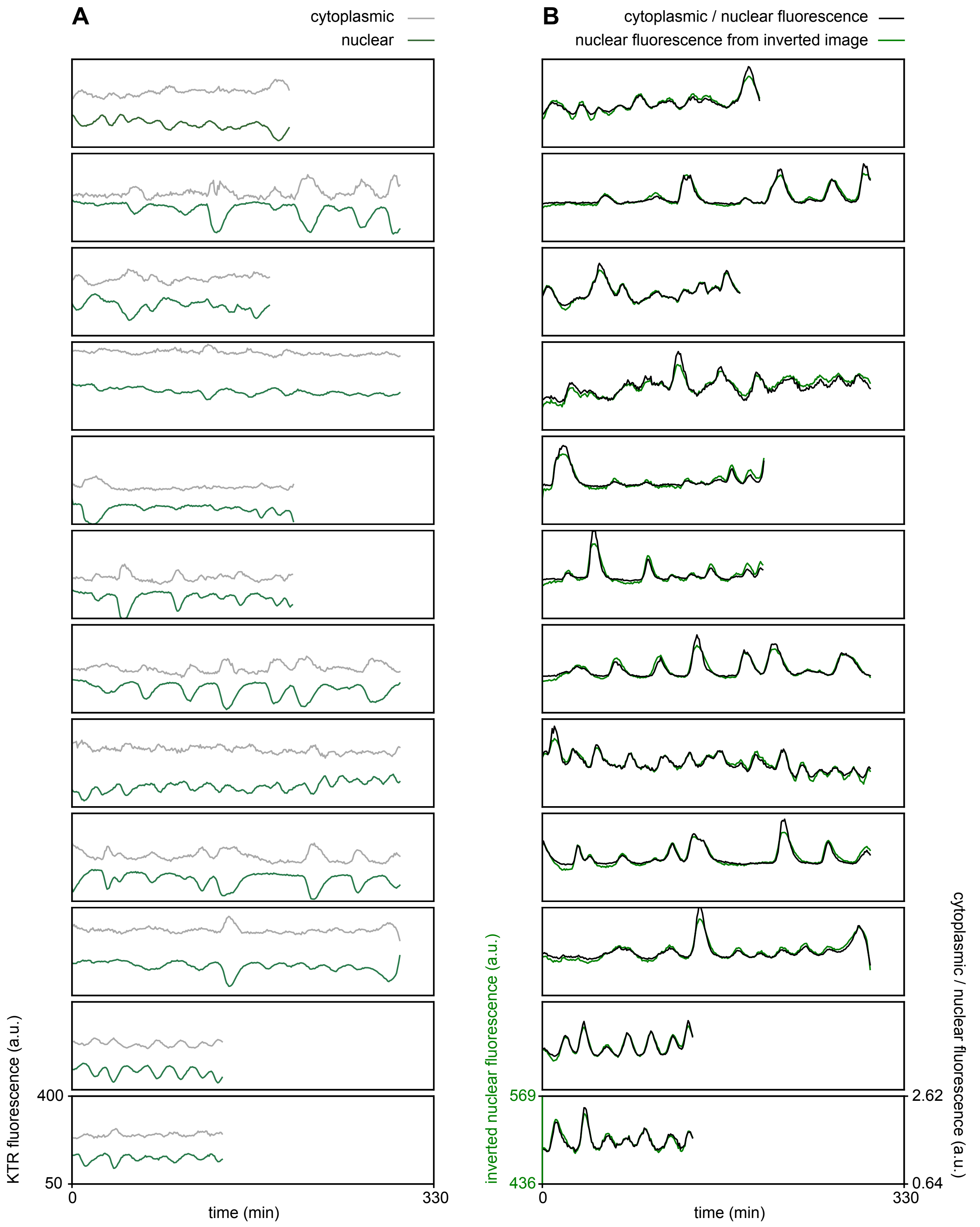

Fig. S2. KTR fluorescence from a nuclear region of interest captures the same dynamics as the cytosol to nuclear ratio. (A) Mean KTR fluorescence in cytoplasmic (gray) and nuclear (green) regions of interest (ROIs). (B) Ratio of cytoplasmic and nuclear fluorescence from A (black), and mean intensity of the nuclear ROI measured on the inverted image (green). In all cases, y axes were centered at the mean and scaled to span 10 standard deviations.

Development • Supplementary information

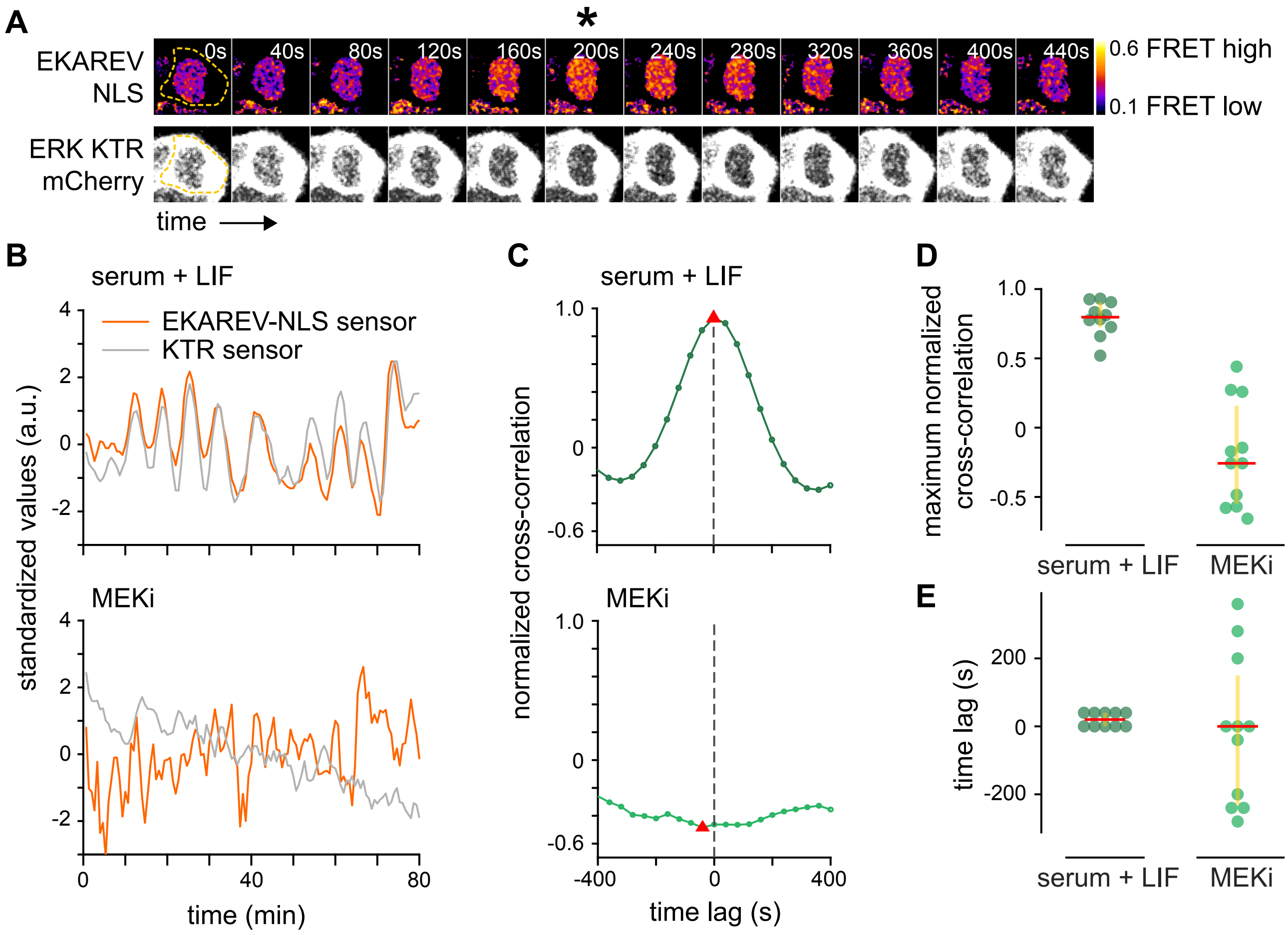

Fig. S3. Orthogonal ERK activity sensors report similar dynamics. (A) Stills from a movie of mESCs growing in serum + LIF medium co-transfected with both an ERK-KTR-mCherry and an EKAREV-CFP-YFP FRET reporter. Upper row shows ratiometric images of a single cell expressing the EKAREV sensor, bottom row shows images of the same cell expressing the KTR-mCherry sensor. High ERK activity detected by the FRET reporter coincides with strong nuclear exclusion of the KTR reporter (asterisk). Gamma values for the KTR montage have been adjusted to 0.86, and the image has been smoothened for the purpose of visualization only. The acquisition rate was 40 s/frame. (B) Single cell trace of mean nuclear intensity of the inverted image (KTR sensor, gray) and mean FRET ratio (EKAREV sensor, orange) in the same nuclear ROI over time in the absence (top) and the presence of MEKi (bottom). FRET ratio was calculated as the ratio between donor emission and acceptor emission upon donor excitation. Traces are standardized by subtracting the mean and then dividing by the standard deviation of every individual trace. (C) Normalized cross correlation for data shown in B between traces of the different sensors as a function of time lag, and its maximum absolute value (red triangle). (D) Summary statistics of maximum absolute values of normalized cross correlations between both reporters over a lag of ± 400 s. (E) Summary statistics of time lag at maximum normalized cross correlation. Red bar in D, E indicates median, yellow vertical bar is the interquartile range. Number of cells: n = 10 (serum + LIF), and n = 11(MEKi).

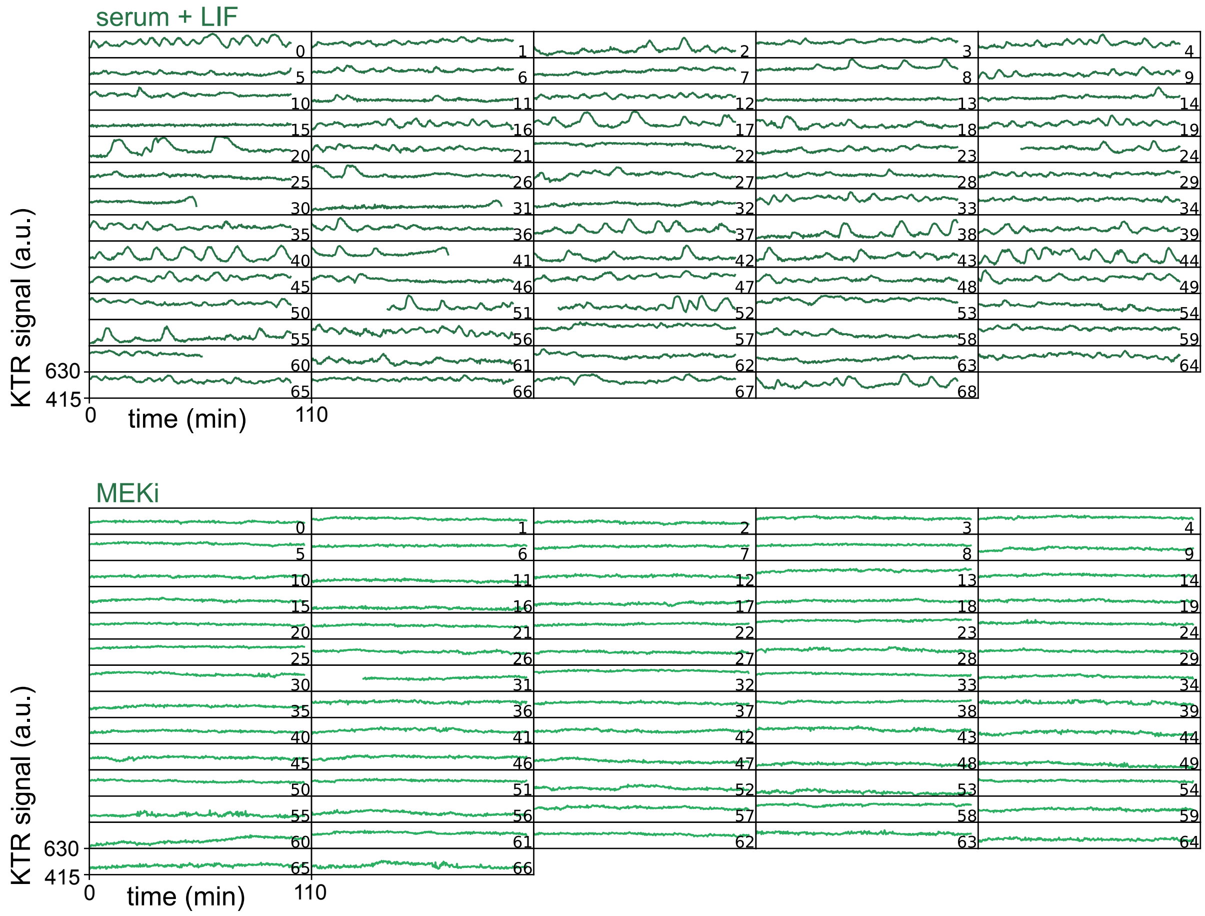

Fig. S4. Dynamics of KTR signal reveals ERK pulsing in serum + LIF conditions. Single cell traces of the KTR signal, obtained as the mean intensity of a nuclear ROI measured on the inverted image, for cells growing in serum + LIF without (top) and with MEKi (bottom). The decrease in KTR signal at the end of the trace in cells 30, 31, 41 and 50 in the condition without MEKi is due to nuclear envelope breakdown as cells enter mitosis. This part of the trace, together with the immediately preceding peak, was trimmed for the downstream analysis. The acquisition rate was 20 s/frame.

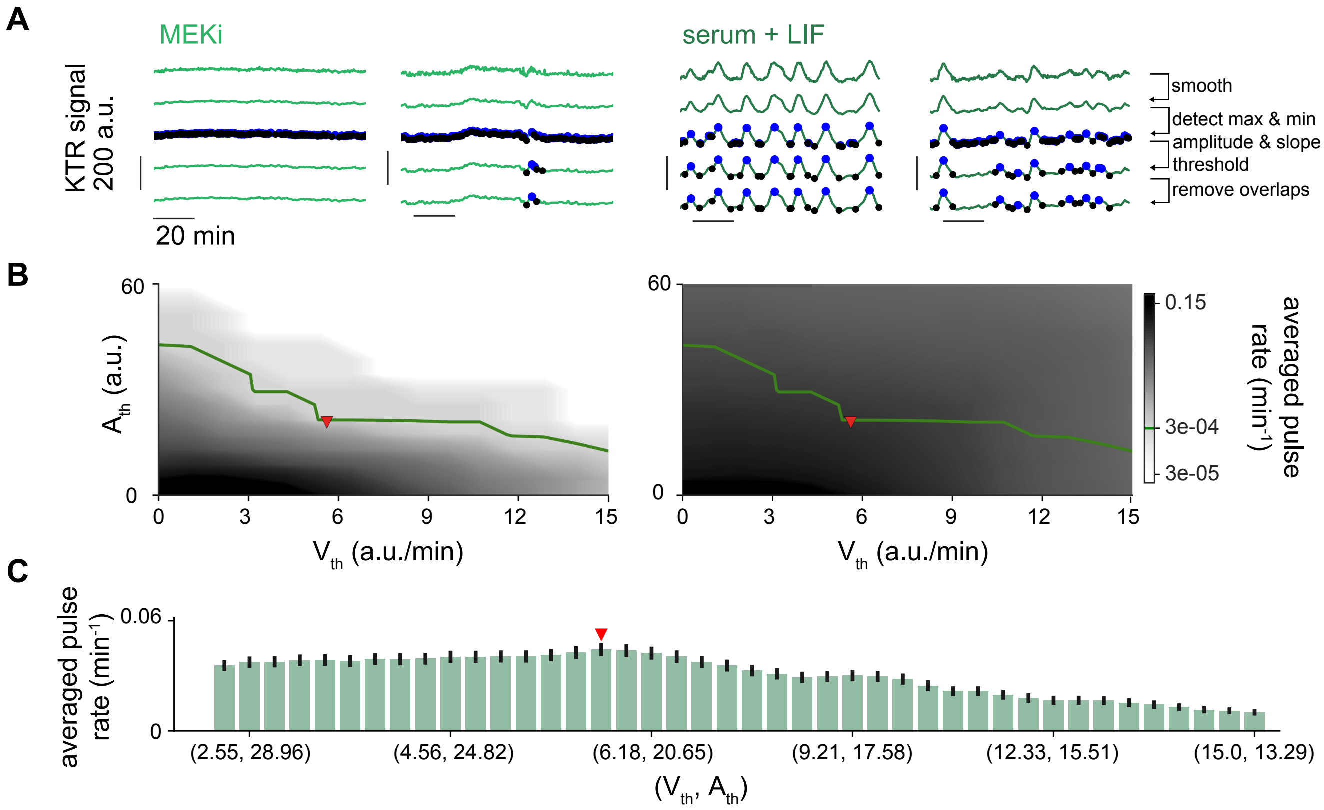

Fig. S5. Pulse recognition and threshold analysis in high resolution time-series. (A) Representative traces of ERK dynamical activity in single ESCs growing in serum + LIF conditions in the presence (two columns on the left) or absence (two columns on the right) of MEKi. Rows illustrate steps in the pulse recognition algorithm: First row shows raw data, second row shows smoothened traces. Blue and black dots in the third row are local maxima and minima. Fourth row shows local maxima and minima that pass the amplitude and slope thresholds. Fifth row shows identified pulses after removing overlaps. Pulses are defined by maxima and their adjacent minima. (B) Average pulse rate as a function of amplitude and slope thresholds for cells growing in serum + LIF with (left) or without (right) MEKi. The level curve where the average pulse rate in MEKi-treated cells is 3×10^{-4}min^{-1} (green line) was used to explore combinations of amplitude and slope threshold values in the condition without inhibitor. (C) Average pulse rate for combinations of amplitude and slope thresholds along the red curve in cells growing in serum + LIF only. Error bar indicates SEM**.** Red triangle in B, C indicates parameter values used for subsequent analysis (Methods, Table S1).

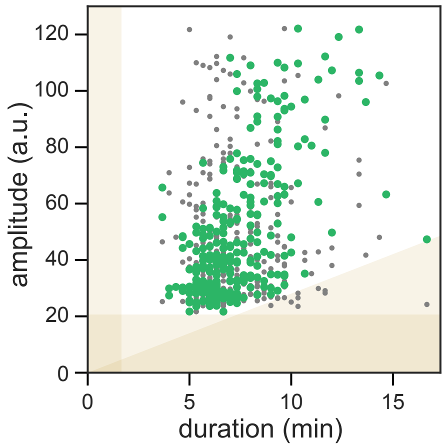

Fig. S6. Correlation of pulse amplitude and duration in cells growing in serum + LIF. Amplitude versus pulse duration for individual pulses (green dots). Gray dots show randomly shuffled values for comparison. Shaded yellow regions indicate the pulse recognition limits determined by the slope (yellow triangle) and amplitude (horizontal bar) thresholds in the pulse recognition algorithm, as well as the sampling resolution (vertical bar).

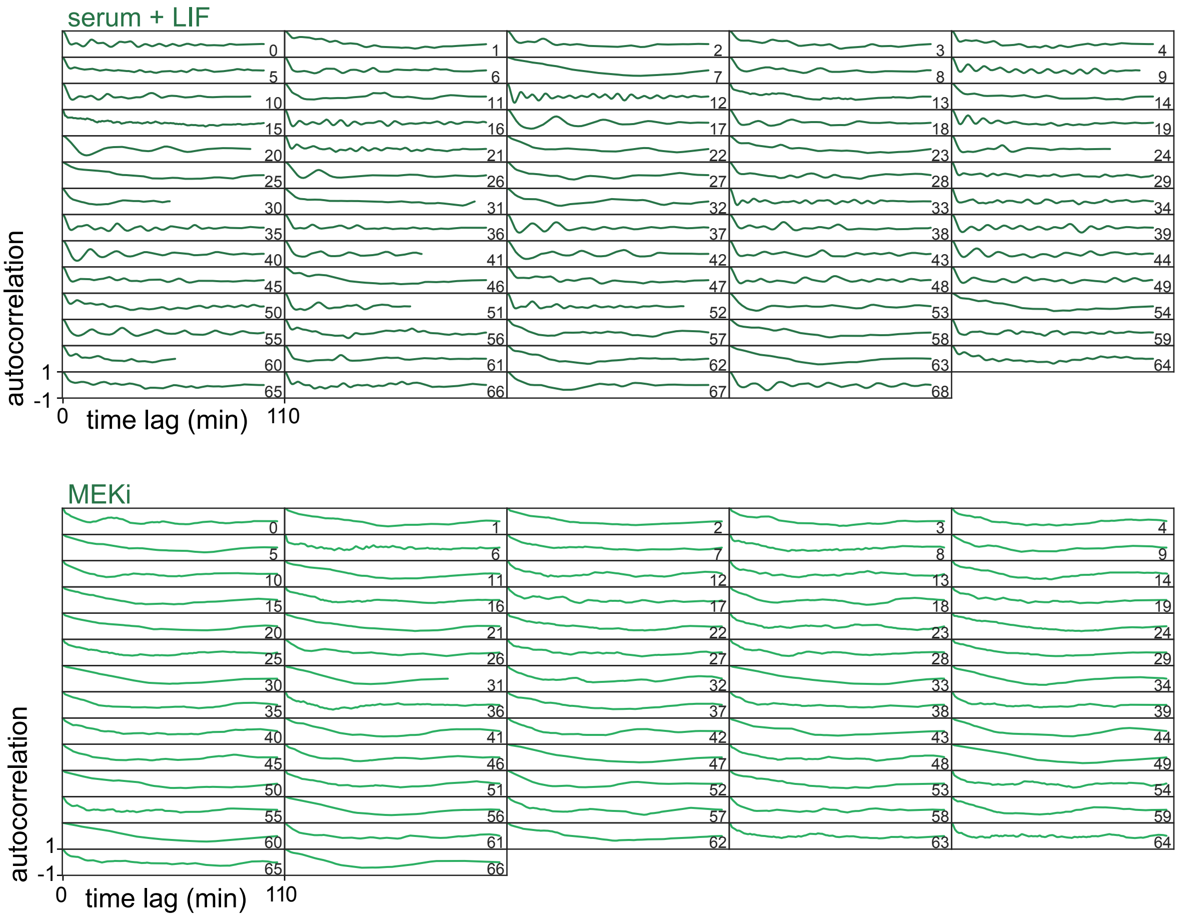

Fig. S7. Signatures of oscillations in the autocorrelation function. Single cell autocorrelation functions from data in Fig. S4, for cells growing in serum + LIF without (top) and with MEKi (bottom). See methods for details of the calculation.

Development • Supplementary information

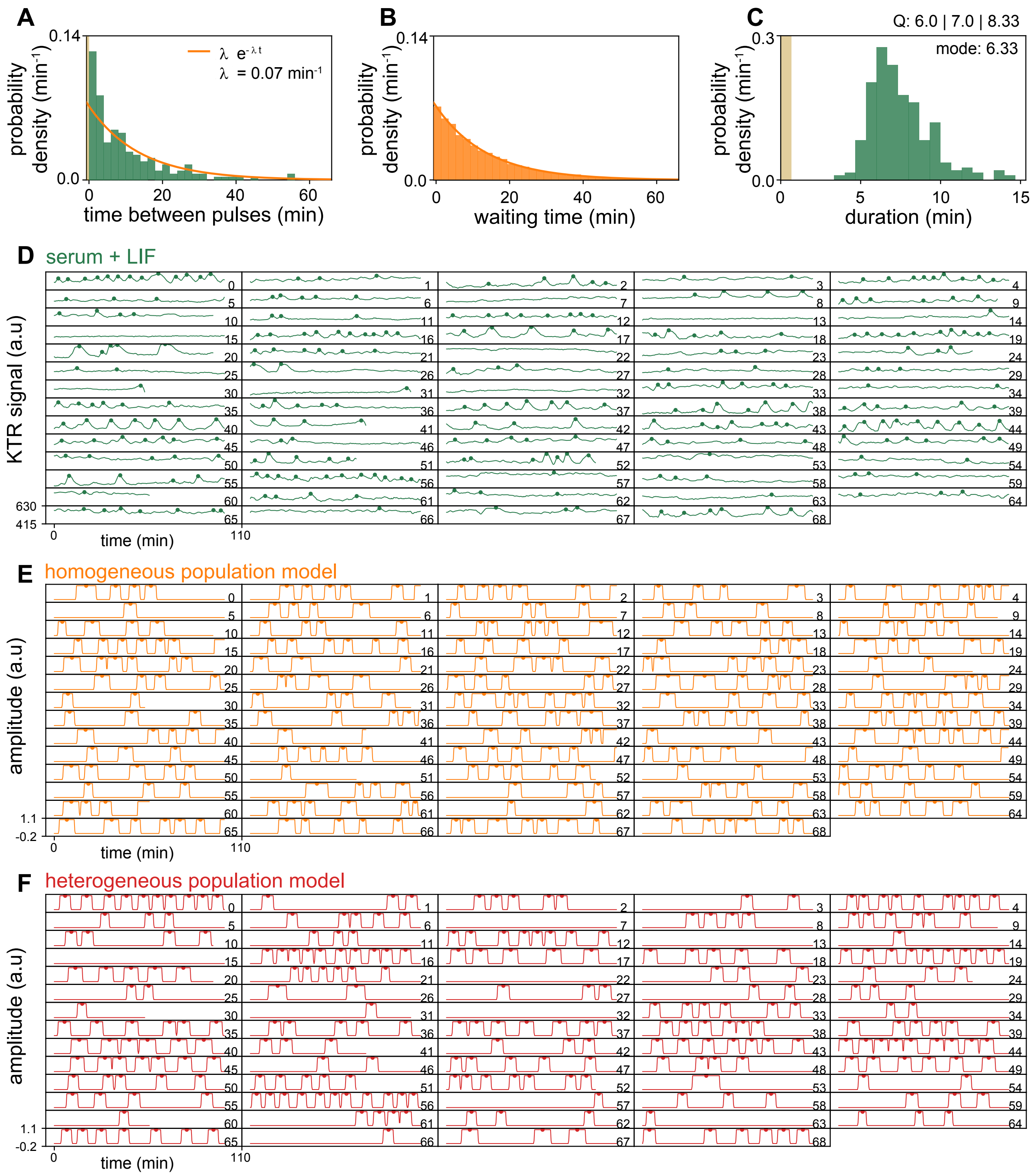

Fig. S8. Stochastic pulsing models. (A) Distribution of silence intervals (green bars) defined as the time between pulses, for the serum + LIF condition (n = 225 pairs of pulses). Exponential fit used in the homogeneous population model (orange line). (B) Waiting time distribution (orange bars) sampled from the exponential fit in A (orange line). (C) Pulse duration distribution for the serum + LIF condition, reproduced from Fig. 2E (n = 289 pulses). A., C. Pulse recognition resolution limit (yellow bar), data from N = 69 cells. A.-C. quartiles (Q) 25, 50 and 75 are indicated (D) Single cell traces of the KTR signal for cells growing in serum + LIF, processed from Fig. S4 and shown here to facilitate comparison with models (green line). Green dots indicate the maxima of the detected pulses. (E) Traces generated from the homogeneous population model (orange line), with pulse maxima (dots). Pulse durations were randomly picked from the experimental distribution in C. (F) Traces generated from the heterogeneous population model (red line), with pulse maxima (dots). All pulse durations in a trace were set as the mean pulse duration in the corresponding experimental trace. Trace lengths in E, F correspond to the length of experimental traces shown in D.

Development • Supplementary information

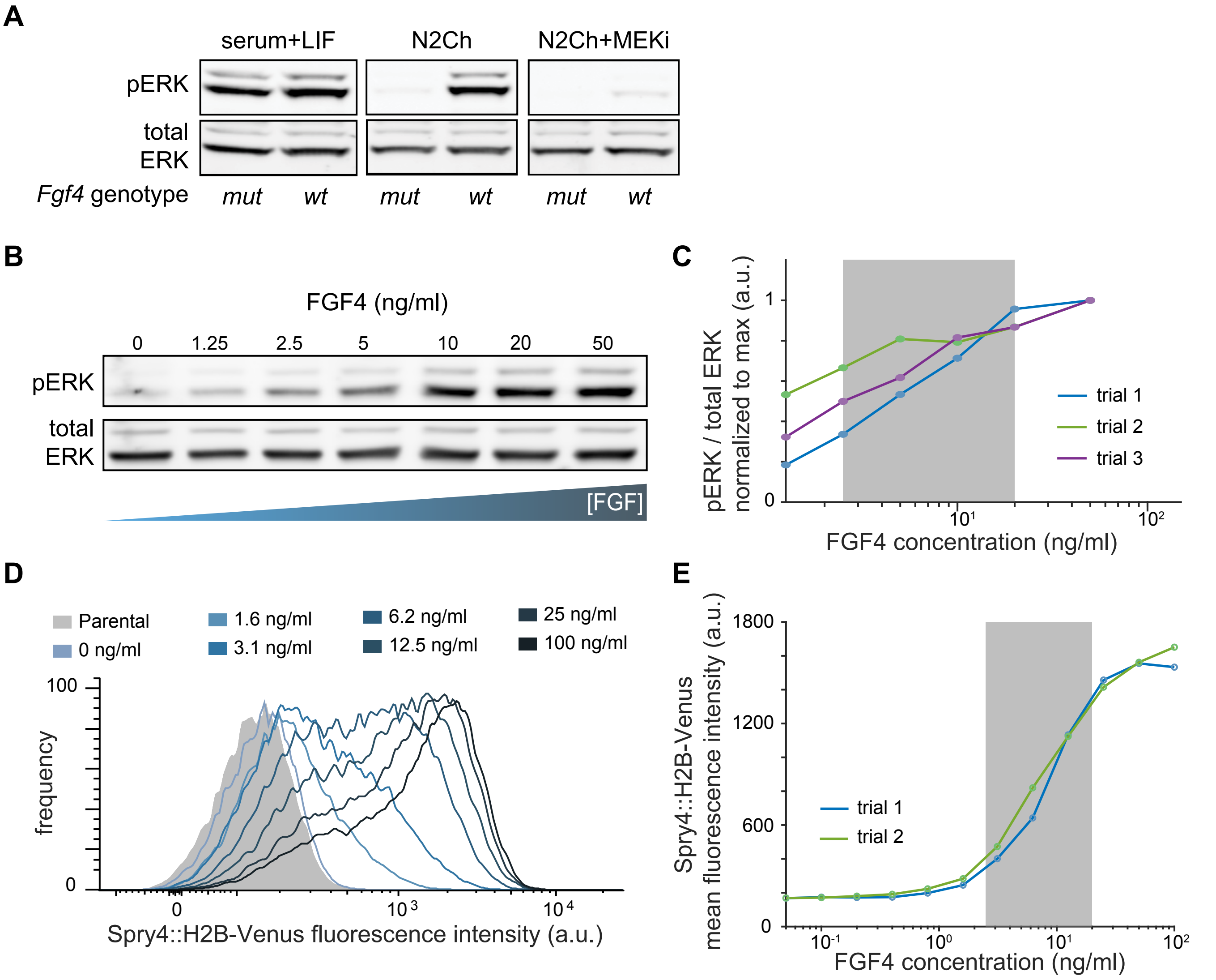

Fig. S9. Dynamic range of signaling and transcriptional response to FGF4 dose in ESCs. (A) Western blot for pERK and total ERK in wild type and Fgf4 mutant cells growing in the indicated media conditions. (B) Representative western blot for pERK and total ERK in Fgf4 mutant cells treated with a range of FGF4 concentrations, with the same experimental protocol as in Fig. 3A. (C) Quantification of western blot data from N = 3 independent experiments. (D) Flow cytometry of Fgf4^{mutant}, Spry4$^{H2B\text{-}Venus/+}$ cells stimulated with a range of FGF4 concentrations. A non-reporter line was used as the negative control (shaded in gray). Frequencies have been normalized to the mode. (E) Quantification of the mean H2B-Venus fluorescence intensity from D. Gray box in C and E indicates the concentration range used in this study from 2.5 to 20 ng/ml.

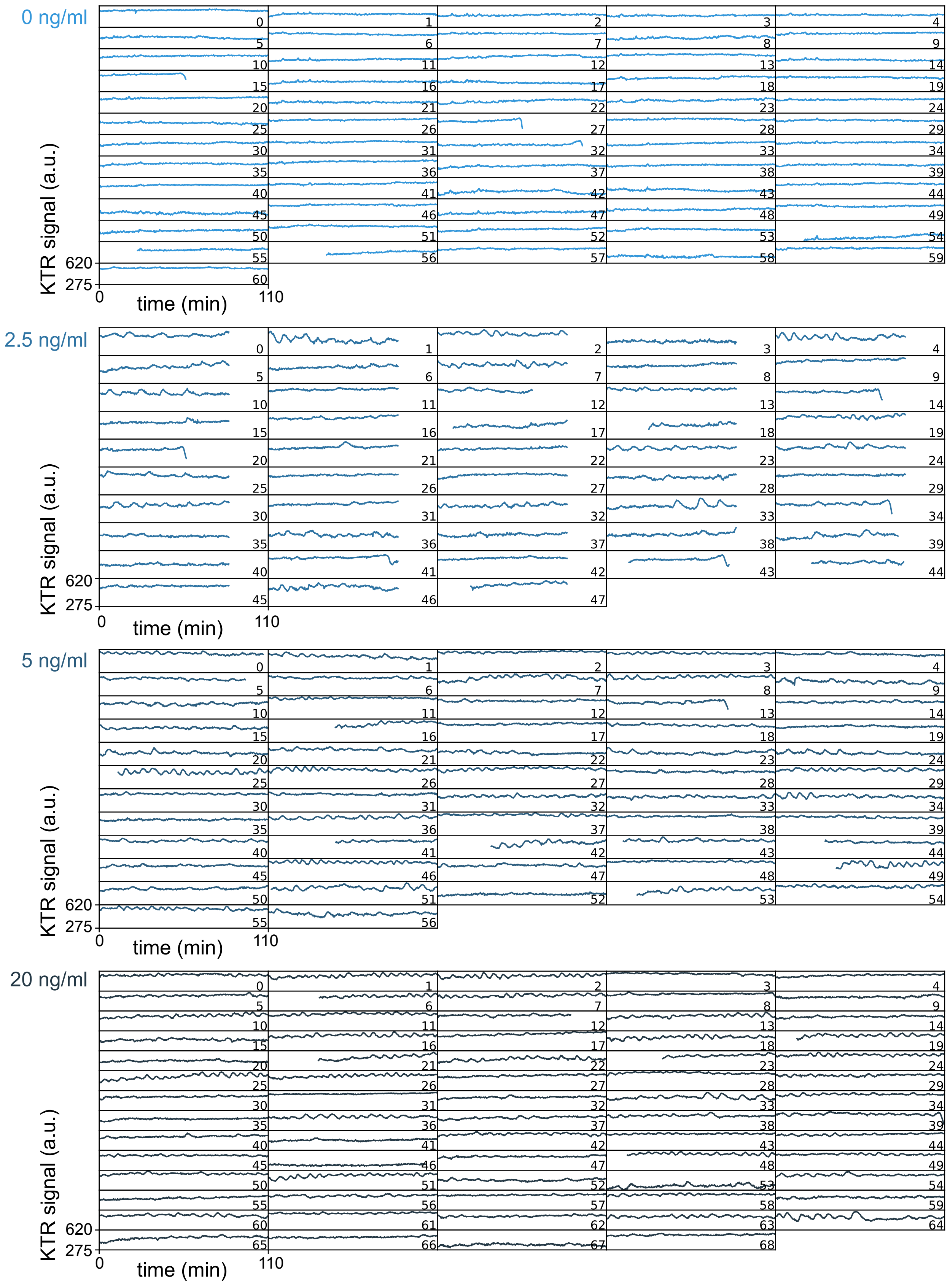

Fig. S10. Dynamics of KTR signal at different FGF4 doses. Traces of the KTR signal obtained as the mean intensity of a nuclear ROI measured on the inverted image in single Fgf4 mutant cells stimulated with indicated doses of FGF4. The decrease in KTR signal at the end of the trace in cells 15, 27 and 32 (0 ng/ml), cells 14, 20, 34, 38, 41, 43, and 44 (2.5 ng/ml), cell 13 (5 ng/ml) and cells 8 and 39 (20 ng/ml) is due to nuclear envelope breakdown as cells enter mitosis. This part of the trace, together with the immediately preceding peak, was trimmed for the downstream analysis. The acquisition rate was 20 s/frame.

Development • Supplementary information

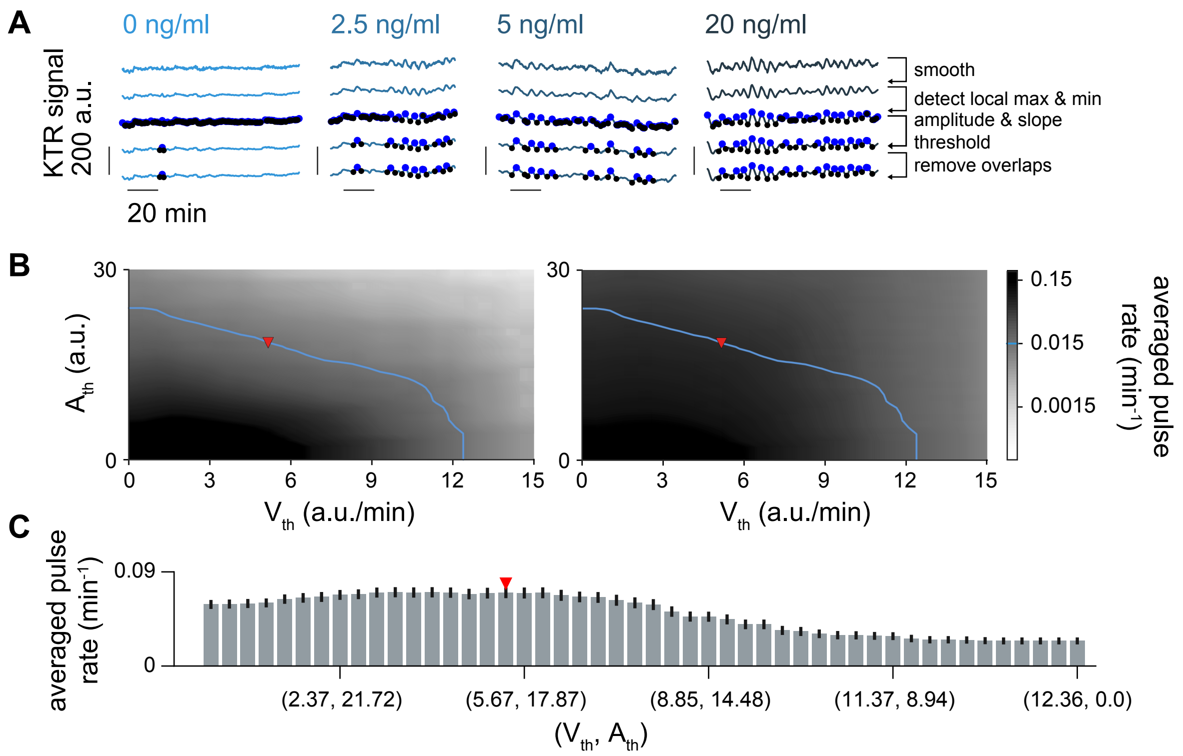

Fig. S11. Pulse recognition and threshold analysis in FGF4 stimulation experiment. (A) Representative traces of ERK dynamical activity in single Fgf4 mutant cells stimulated with different doses of FGF4 (columns), with colors as in Fig. 3. Rows illustrate steps in the pulse recognition algorithm: First row shows raw data, second row shows smoothened traces. Blue and black dots in the third row are local maxima and minima. Fourth row shows local maxima and minima that pass the amplitude and slope thresholds. Fifth row shows identified pulses after removing overlaps. Pulses are defined by maxima and their adjacent minima. (B) Average pulse rate as a function of amplitude and slope thresholds for Fgf4 mutant without stimulation (left) and stimulated with 20 ng/ml FGF4 (right). The level curve where the average pulse rate in unstimulated cells is 0.015 min^{-1} (blue line) was used to explore combinations of amplitude and slope threshold values in the stimulated conditions. (C) Average pulse rate for combinations of amplitude and slope thresholds along the blue curve in Fgf4 mutant cells stimulated with 20 ng/ml of FGF4. Error bar indicates s.e.m.. Red triangle in B, C indicates parameter values used for subsequent analysis (Materials and Methods, Table S1).

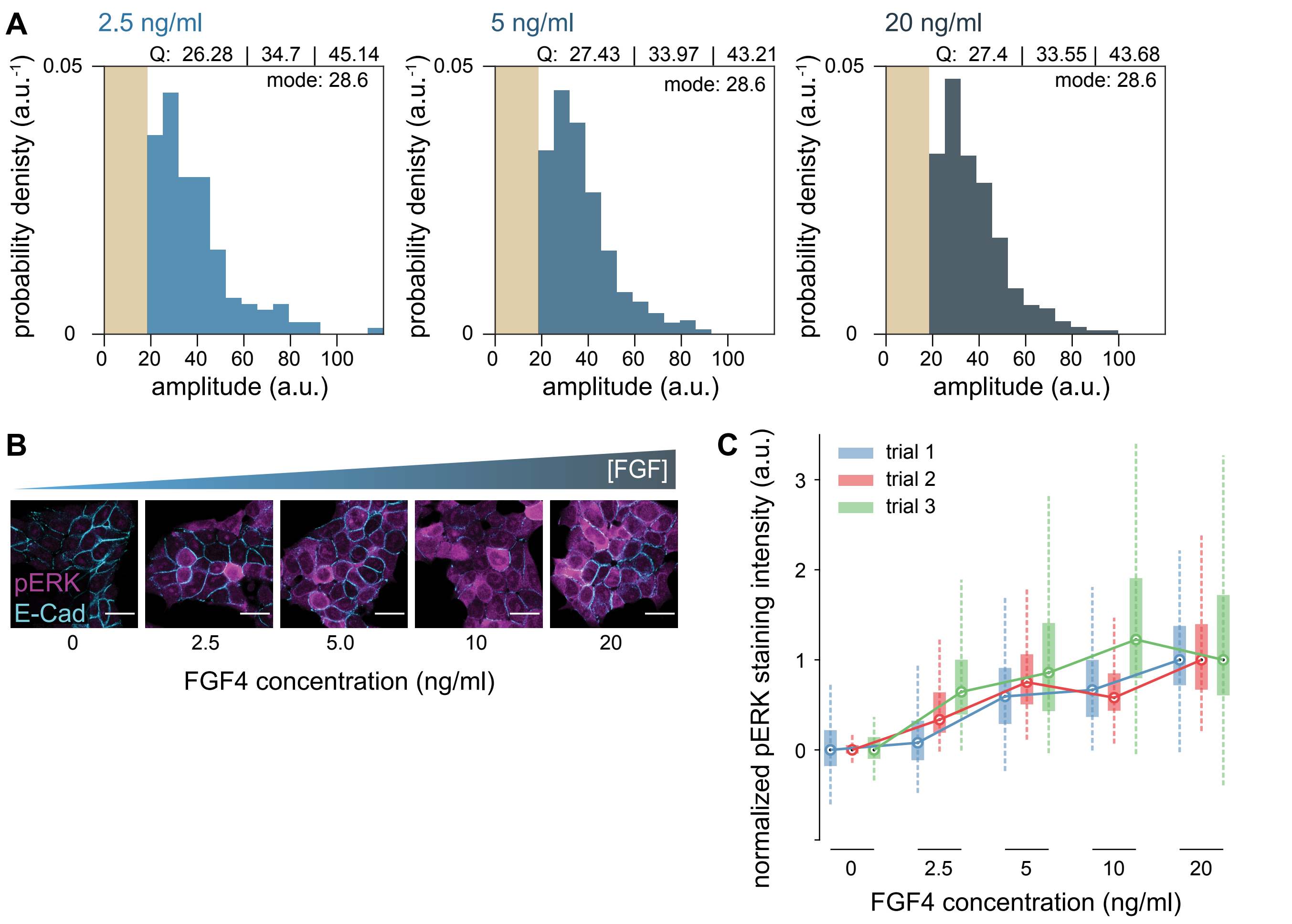

Fig. S12. Distribution of pulse amplitudes and single cell pERK levels at different FGF4 doses. (A) Distribution of sensor pulse amplitudes in Fgf4 mutant cells stimulated with different doses of FGF4. The number of pulses was n = 164 (2.5 ng/ml), n = 426 (5 ng/ml) and n = 544 (20 ng/ml). Pulse recognition resolution limit (yellow bar) and quartiles (Q) 25, 50 and 75 are indicated. (B) Immunostaining of Fgf4 mutant cells for pERK (magenta) and E-Cadherin (cyan) to outline cell boundaries. Cells were treated with indicated concentrations of FGF4, with the experimental protocol depicted in Fig. 3A. Scale bar = 20 µm. (C) Boxplots of pERK intensity in single cells from three different trials, stained as in B. Circles indicate median, solid boxes interquartile range, whiskers are 1.5 times the interquartile range. In each trial, we first subtracted the median of the 0 ng/ml condition from all single cell measurements, and then normalized to the median of the 20 ng/ml condition.

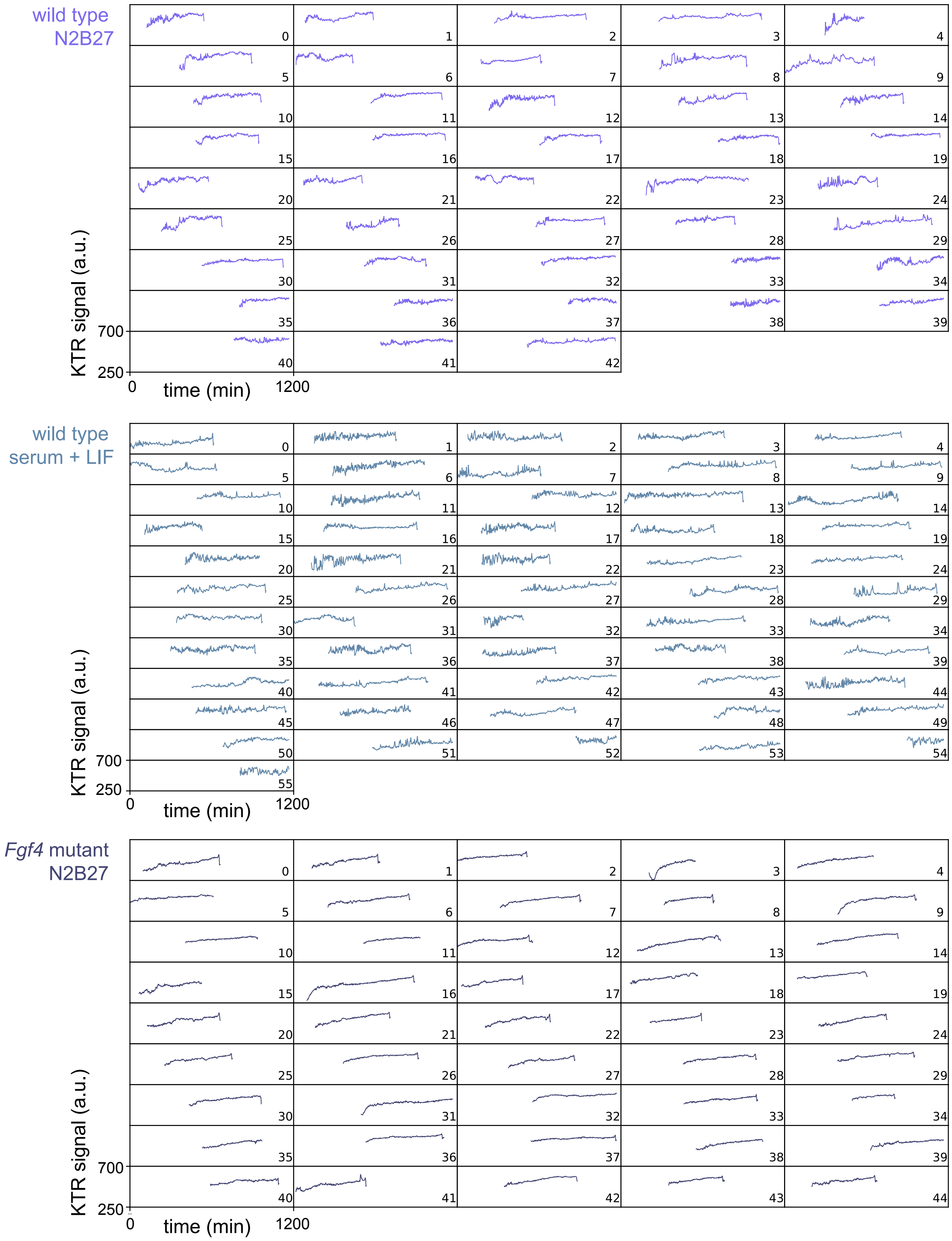

Fig. S13. Dynamics of KTR signal in long term recordings. Traces of the KTR signal obtained as the mean intensity of a nuclear ROI measured on the inverted image in wild type cells growing in N2B27 (top), serum + LIF (middle), and in Fgf4 mutant cells growing in N2B27 (bottom). The acquisition rate was 105 s/frame. The scale of the horizontal axis represents absolute experimental time. Single cell tracks begin immediately after a cell division event and are plotted relative to absolute experimental time. Most traces end with exclusion of the sensor from the nucleus before cell division. This part of the traces, together with the immediately preceding peak, was trimmed for the downstream analysis.

Development • Supplementary information

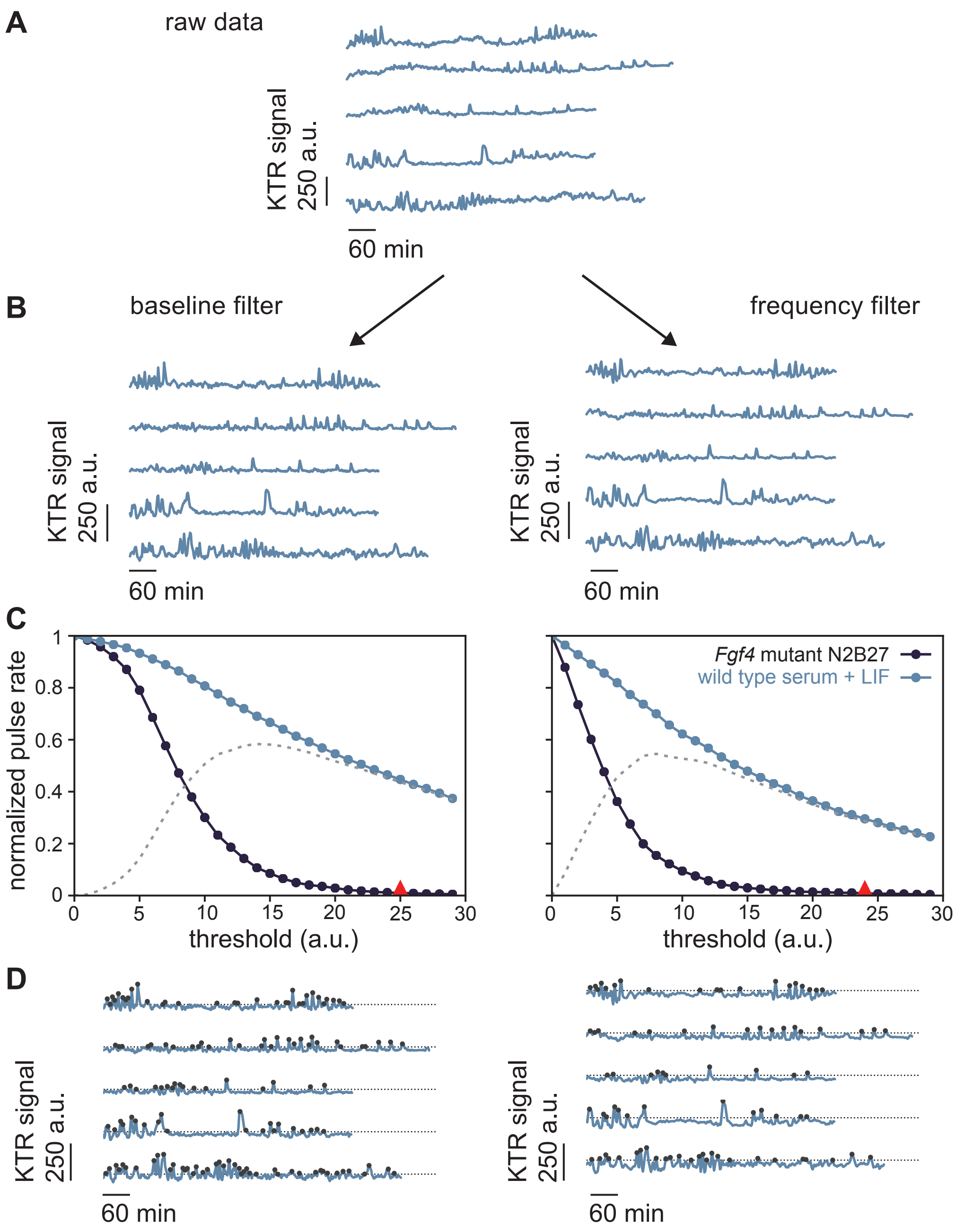

Fig. S14. Peak detection and threshold analysis in long term time series. (A) Representative traces of KTR signal from long term recordings in single wild type cells growing in serum + LIF. Traces have been aligned relative to the time of cell birth for this illustration. B - D illustrate the two filtering strategies, left column corresponds to baseline filtering and right column to band-pass filtering (Methods). (B) Same traces as in A following filtering. (C) Plot of normalized pulse rate vs. filtered KTR signal threshold to explore how the number of detected pulses depends on threshold value. Fgf4 mutant cells growing in N2B27 in dark blue, wild type cells growing in serum + LIF in light blue. The gray dotted line represents the difference of the normalized pulse rates between the experimental conditions considered. The position of the selected intensity threshold value I_{th} is marked with a red triangle. (D) Same traces as in B with identified peaks (black dots). The dotted gray line in indicates the selected threshold parameter I_{th}.

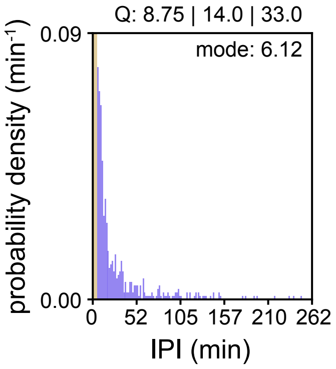

Fig. S15. Distribution of interpulse intervals in long-term data. IPI probability density distribution for baseline filtered long-term data. Pulse recognition resolution limit (yellow bar) and quartiles (Q) 25, 50 and 75 are indicated.

Development • Supplementary information

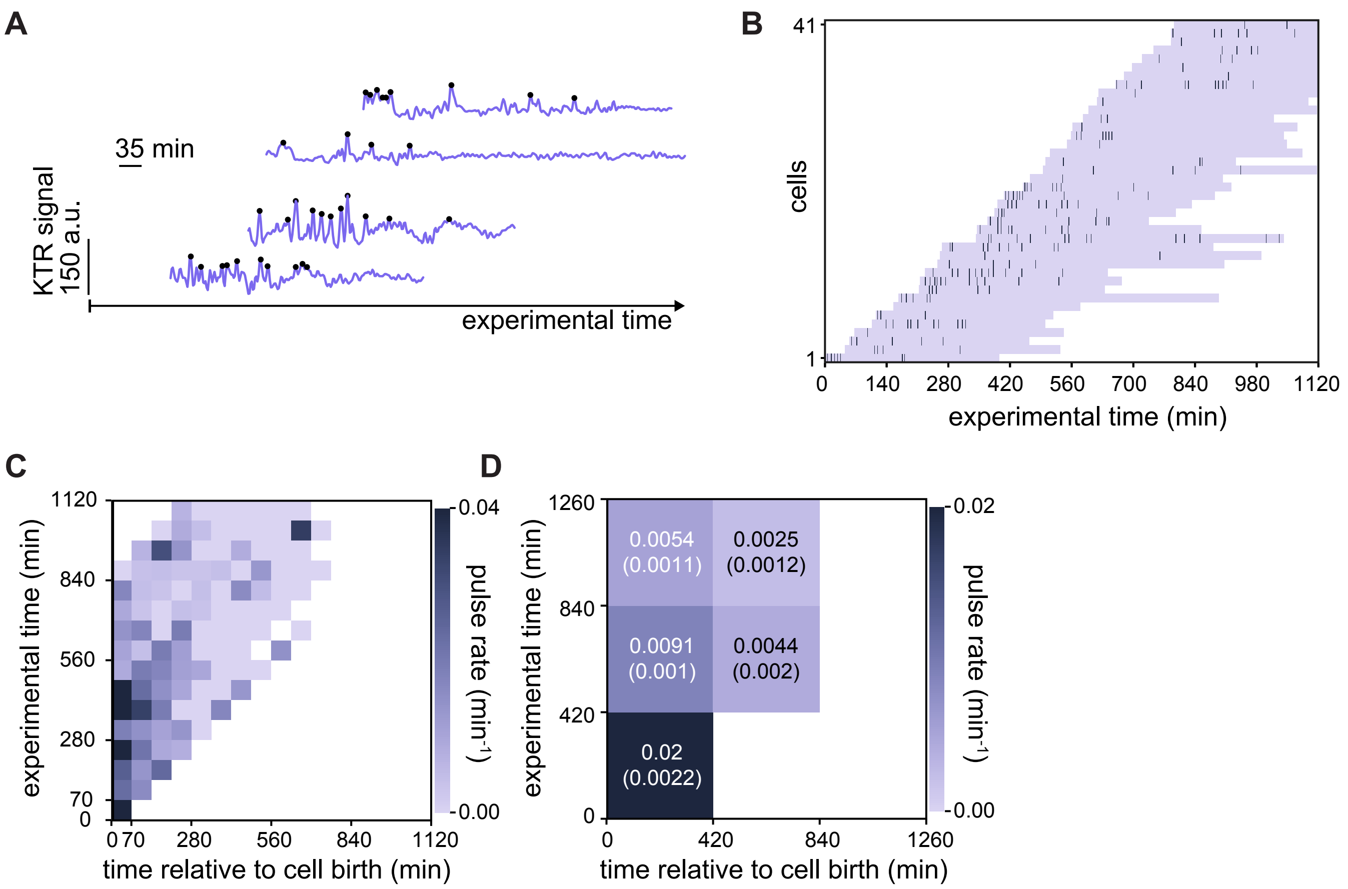

Fig. S16. The alternative frequency filtering strategy confirms prevalent ERK pulsing early in the cell cycle. (A) Representative traces of ERK dynamical activity from the same experiment reported in Fig. 4, now following the alternative frequency filtering strategy. Identified peaks indicated as black dots. (B) Raster plot displaying the timing of ERK activity peaks across the cell cycle in frequency-filtered data. Lavender horizontal bands extend from birth to division of single cells, dark vertical bars represent peaks. Single cell tracks begin immediately after a cell division event and are plotted relative to absolute experimental time. (C) Pulse rate map for the data shown in B. Time is discretized into 70 min bins. (D) Coarse grained pulse rate map showing average pulse rate and its estimated error with 420 min binning.

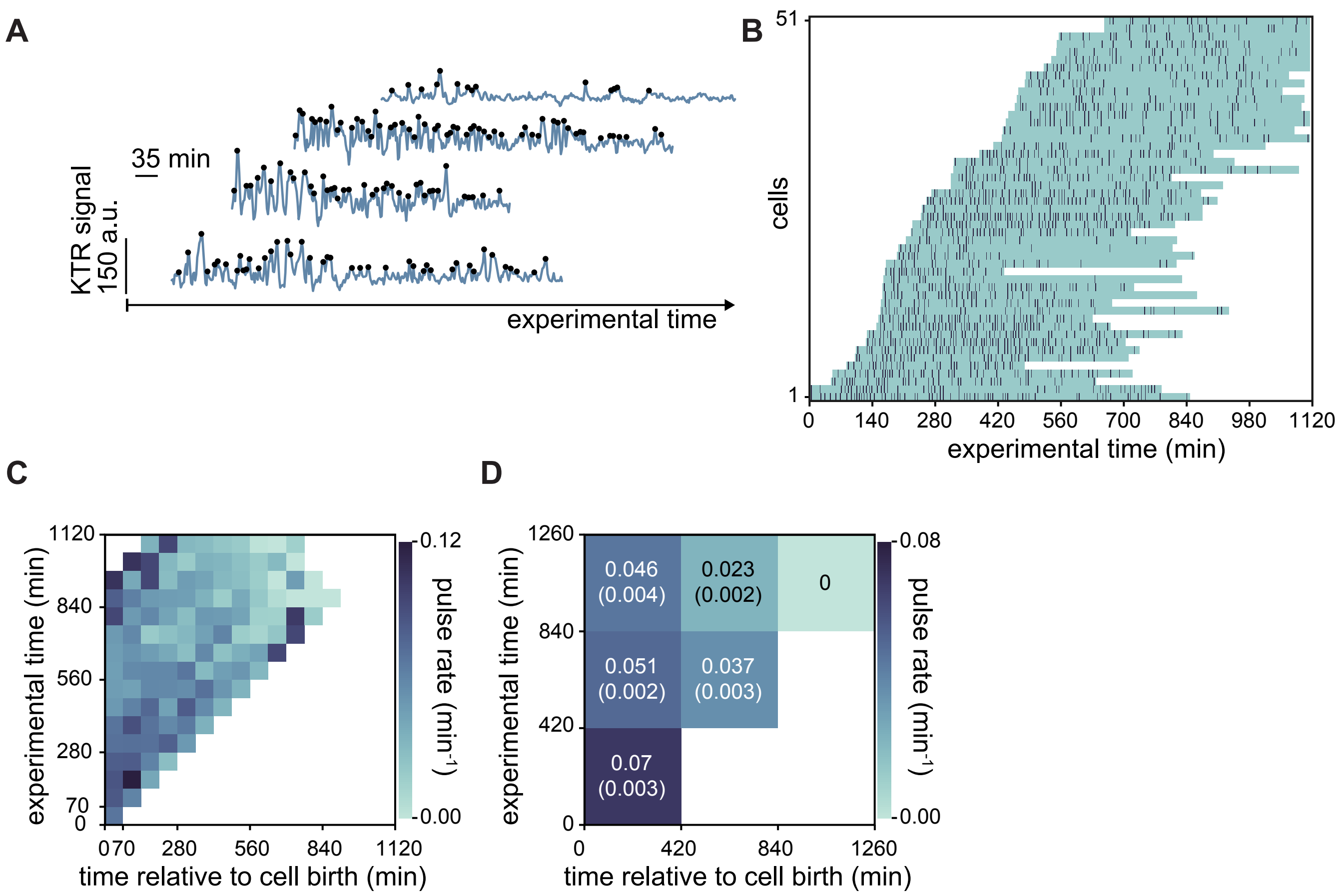

Fig. S17. Prevalent ERK pulsing early in the cell cycle in cells growing in serum + LIF. (A) Representative traces of ERK dynamical activity with identified peaks (black dots) in wild type cells growing in serum + LIF. Experimental protocol and baseline filtering strategy are the same as in Fig. 4. (B) Raster plot displaying the timing of ERK activity peaks across the cell cycle in cells growing in serum + LIF. Teal horizontal bands extend from birth to division of single cells, dark vertical bars represent peaks. Single cell tracks begin immediately after a cell division event and are plotted relative to absolute experimental time. (C) Pulse rate map for the data shown in B. Time is discretized into 70 min bins. (D) Coarse grained pulse rate map showing average pulse rate and its estimated error with 420 min binning.

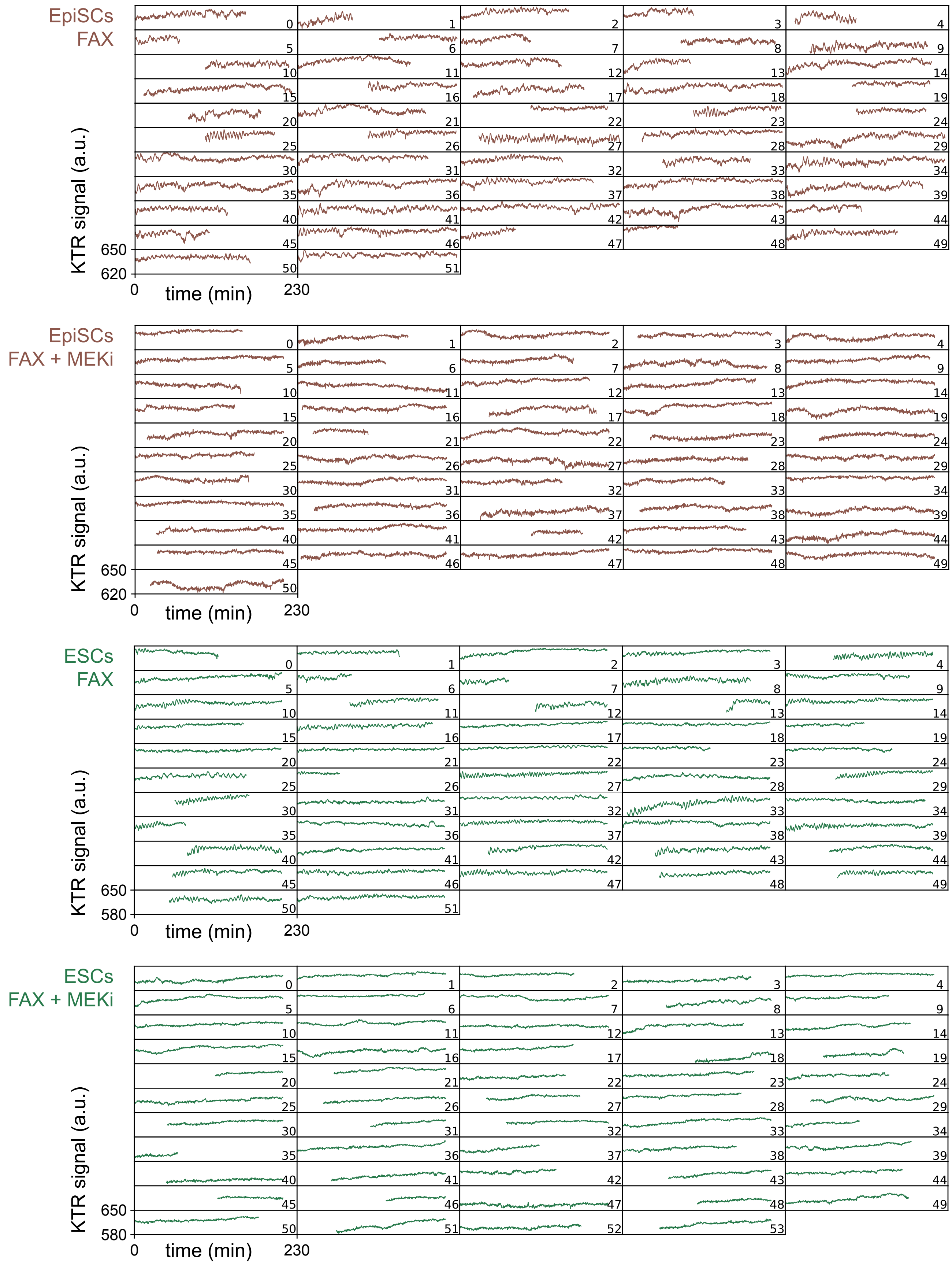

Fig. S18. Dynamics of KTR signal in EpiSCs and ESCs growing in FAX medium. Traces of the KTR signal obtained as the mean intensity of a nuclear ROI measured on the inverted image in single EpiSCs or ESCs growing in FAX medium with or without MEKi. The decrease in KTR signal at the end of the trace in EpiSC cell 27 and ESC cell 1 growing in FAX without MEKi is due to nuclear envelope breakdown as cells enter mitosis. This part of the trace, together with the immediately preceding peak, was trimmed for the downstream analysis. The acquisition rate was 20 s/frame.

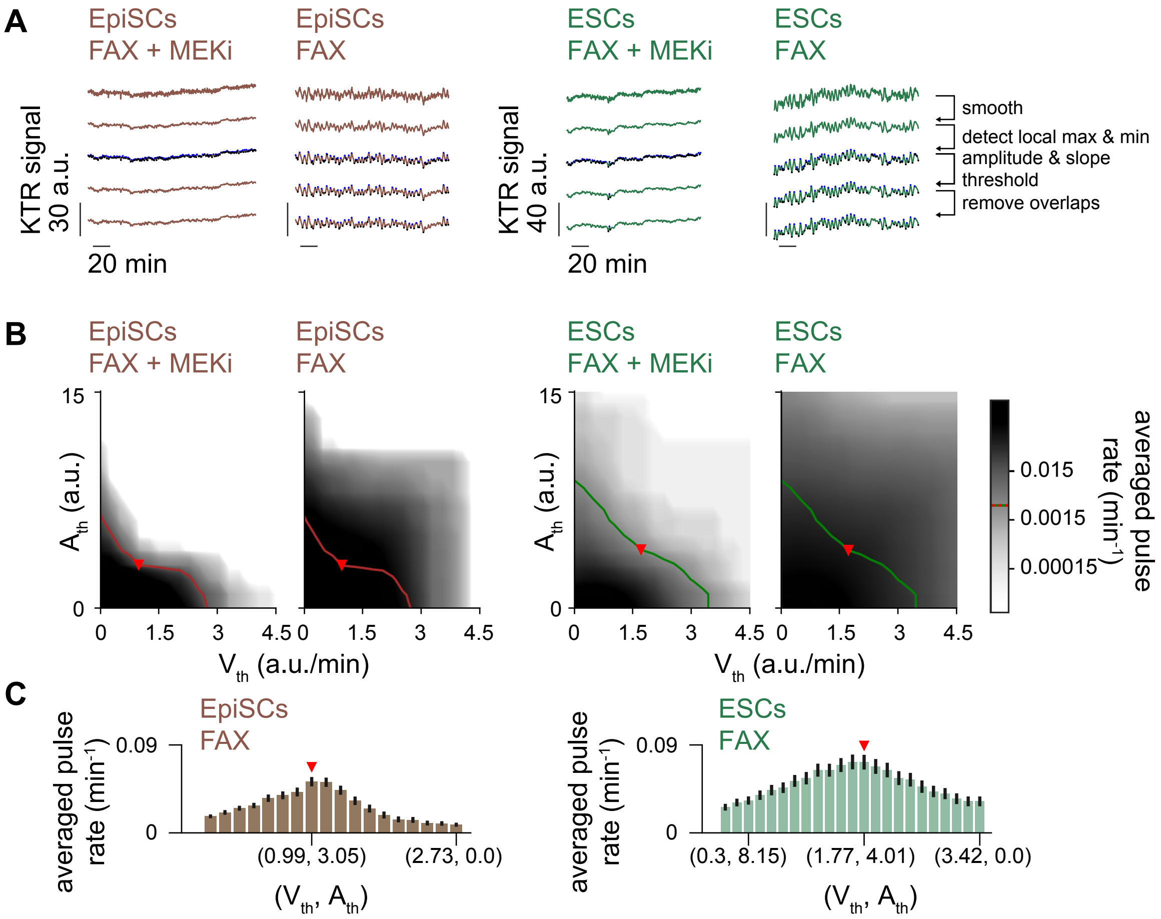

Fig. S19. Pulse recognition and threshold analysis for EpiSCs. (A) Representative traces of ERK dynamical activity in single EpiSCs and ESCs cultured in FAX with or without MEKi (columns), with colors as in Fig. 5. Rows illustrate steps in the pulse recognition algorithm: first row shows raw data, second row shows smoothened traces. Blue and black dots in the third row are local maxima and minima, respectively. Fourth row shows local maxima and minima that pass the amplitude and slope thresholds. Fifth row shows identified pulses after removing overlaps. Pulses are defined by maxima and their adjacent minima. (B) Average pulse rate as a function of amplitude and slope thresholds for different experimental conditions as indicated. The level curves for ESCs and EpiSCs where the average pulse rate in FAX + MEKi is 0.003 min^{-1} (red and green lines, respectively) were used to explore combinations of amplitude and slope threshold values in the corresponding cell types cultured in FAX alone. (C) Average pulse rate for combinations of amplitude and slope thresholds along the level curves shown in B for EpiSCs (left) and ESCs (right) cultured in FAX alone. Error bar indicates s.e.m.. Red triangles in B, C indicate parameter values used for subsequent analysis (Materials and Methods, Table S1).

Table S1. Pulse detection parameters from the threshold analysis protocol for short-term high resolution datasets.

Experiment	Negative control condition	Positive control condition	Exploratory parameter space	Level curve (δ_p^*)	Selected amplitude threshold	Selected slope threshold
Wild type constant stimulation	MEKi	serum + LIF	v_{th} : (0-15) $\frac{\text{a.u}}{\text{min}}$ with 0.25 $\frac{\text{a.u}}{\text{min}}$ resolution. A_{th} : (0-60) a.u. with 1 a.u. resolution.	$3 \times 10^{-4}\ \text{min}^{-1}$	20.68 a.u.	5.61 $\frac{\text{a.u}}{\text{min}}$
Fgf4 mutant different FGF4 stimulation	*Fgf4* mutant 0 ng/ml FGF4	*Fgf4* mutant 20 ng/ml FGF4	v_{th} : (0-15) $\frac{\text{a.u}}{\text{min}}$ with 0.5 $\frac{\text{a.u}}{\text{min}}$ resolution. A_{th} : (0-40) a.u. with 1 a.u. resolution.	$0.015\ \text{min}^{-1}$	18.47 a.u.	5.16 $\frac{\text{a.u}}{\text{min}}$
EpiSCs in FAX	EpiSCs in FAX + MEKi	EpiSCs in FAX	v_{th} : (0-15) $\frac{\text{a.u}}{\text{min}}$ with 1.8 $\frac{\text{a.u}}{\text{min}}$ resolution. A_{th} : (0-60) a.u. with 0.6 a.u. resolution.	$3 \times 10^{-3}\ \text{min}^{-1}$	3.05 a.u.	0.99 $\frac{\text{a.u}}{\text{min}}$
ESCs in FAX	ESCs in FAX + MEKi	ESCs in FAX	v_{th} : (0-15) $\frac{\text{a.u}}{\text{min}}$ with 1.8 $\frac{\text{a.u}}{\text{min}}$ resolution. A_{th} : (0-60) a.u. with 0.6 a.u. resolution.	$3 \times 10^{-3}\ \text{min}^{-1}$	4.06 a.u.	1.71 $\frac{\text{a.u}}{\text{min}}$

Development • Supplementary information

Table S2. Kolmogorov-Smirnov two sample test p-value, K[x, y]. Table cells are color coded according to different p-value thresholds, with the color code is given at the table bottom. The total number of data points for each condition is indicated on the rightmost column of the table. The low number of pulses at 0 ng/ml precluded statistical analysis of this condition.

**Pulse rate**				
**X / Y**	**2.5 ng**	**5 ng**	**20 ng**	**Total data**
**2.5 ng**	1.000	0.009	0.005	48
**5 ng**	0.009	1.000	0.727	57
**20 ng**	0.005	0.727	1.000	69
**Pulse duration**				
**X / Y**	**2.5 ng**	**5 ng**	**20 ng**	**Total data**
**2.5 ng**	1.000	0.059	0.002	164
**5 ng**	0.059	1.000	0.340	426
**20 ng**	0.002	0.340	1.000	544
**Amplitude**				
**X / Y**	**2.5 ng**	**5 ng**	**20 ng**	**Total data**
**2.5 ng**	1.000	0.432	0.586	164
**5 ng**	0.432	1.000	0.835	426
**20 ng**	0.586	0.835	1.000	544
**Interpulse interval**				
**X / Y**	**2.5 ng**	**5 ng**	**20 ng**	**Total data**
**2.5 ng**	1.000	0.044	< 0.001	124
**5 ng**	0.044	1.000	0.014	370
**20 ng**	< 0.001	0.014	1.000	479
**Consecutive pulses**				
**X / Y**	**2.5 ng**	**5 ng**	**20 ng**	**Total data**
**2.5 ng**	1.000	0.099	0.082	48
**5 ng**	0.099	1.000	0.837	57
**20 ng**	0.082	0.837	1.000	69
**References**				
> 0.05	< 0.05	< 0.01	< 0.005	< 0.001

Development • Supplementary information

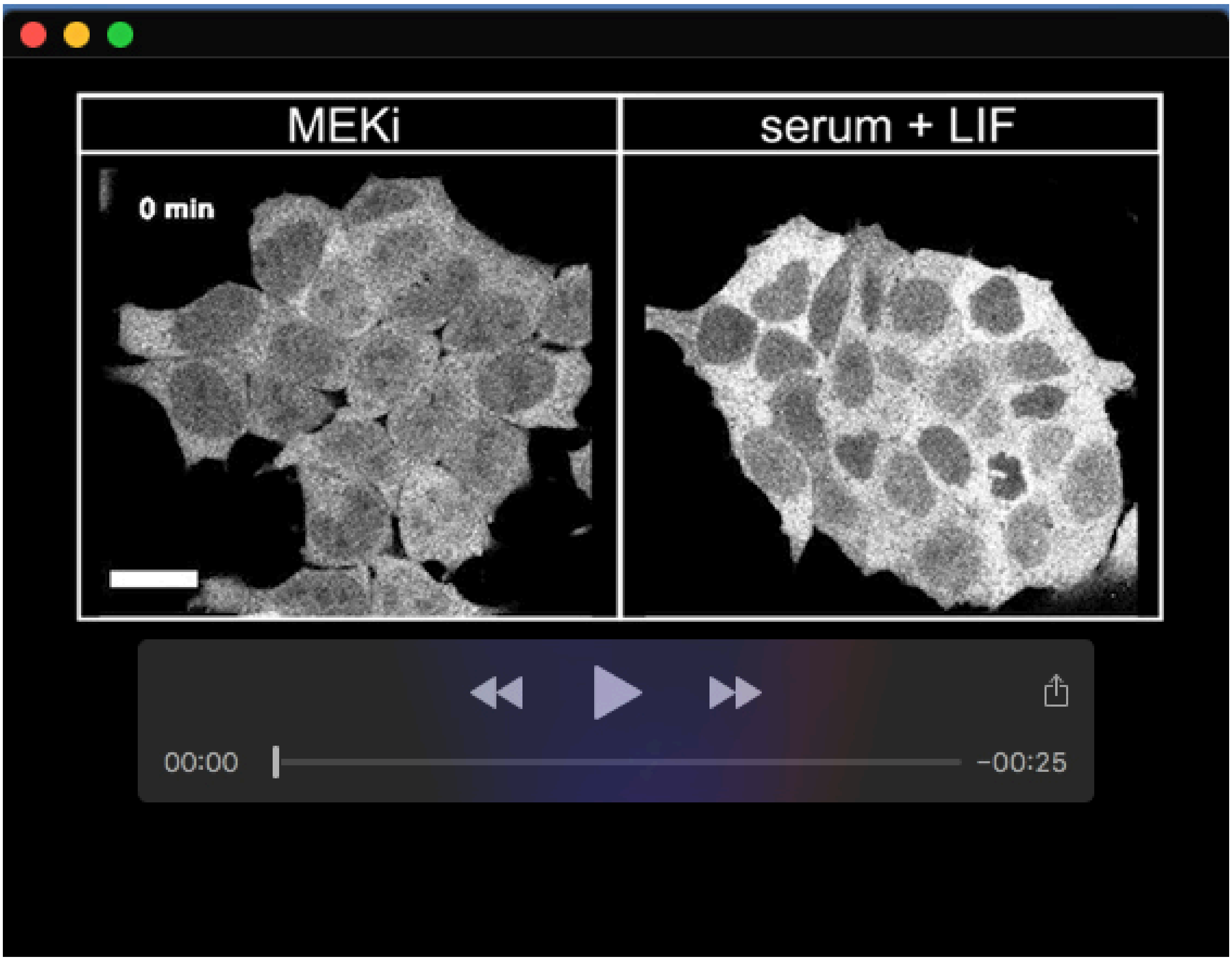

Movie 1. KTR sensor reveals dynamic ERK activity in ESCs growing in serum + LIF. Time-lapse imaging of ERK-KTR expressing cells growing in serum + LIF with (left) and without MEKi (right). Frame rate is 20 s, total duration is 101 min. Scale bar: 20 μm.

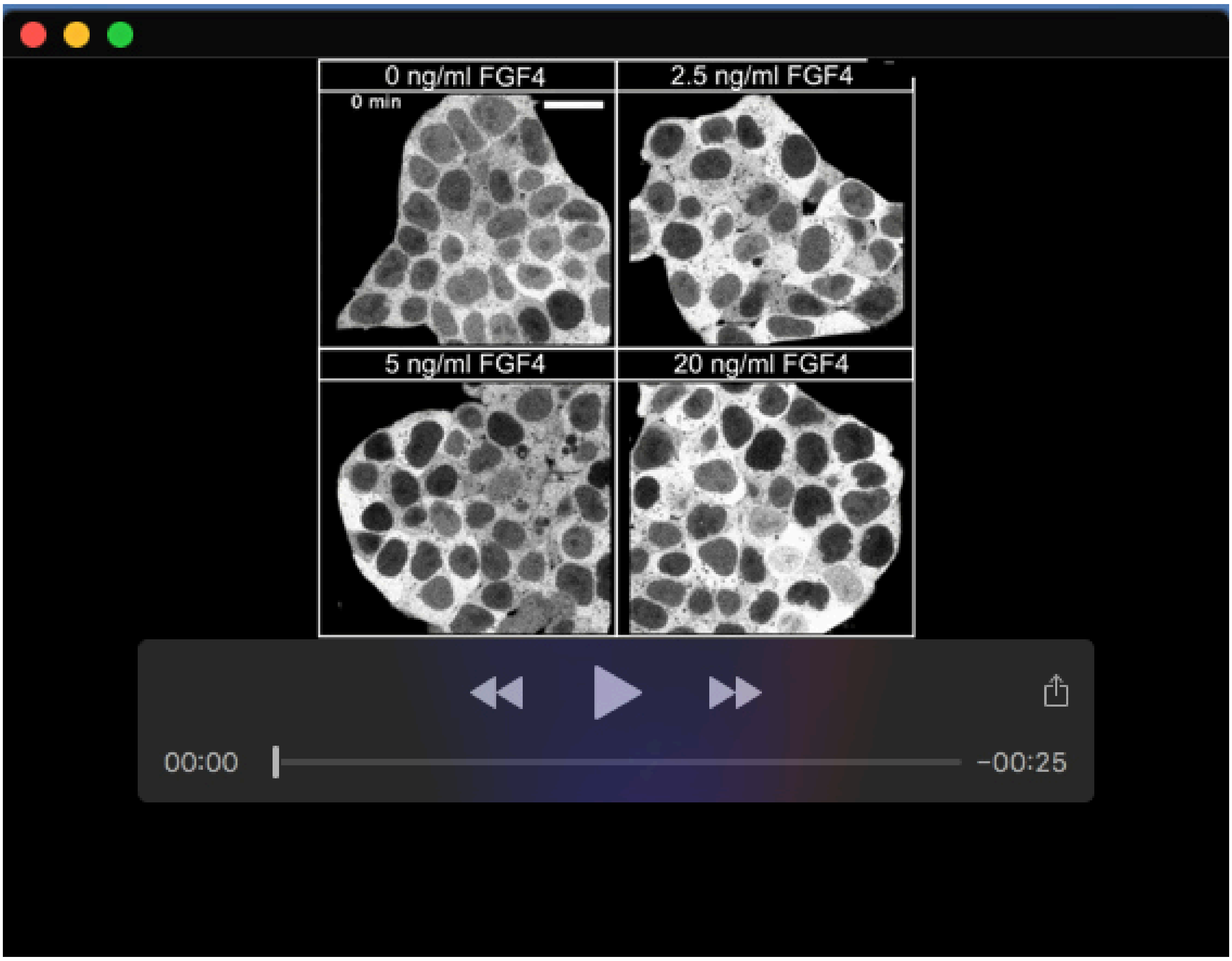

Movie 2. Dynamic ERK activity in Fgf4 mutant cells stimulated with recombinant FGF4. Time-lapse imaging of Fgf4 mutant cells expressing the ERK-KTR sensor cultured without FGF stimulation (top left), or stimulated with 2.5 ng/ml (top right), 5 ng/ml (bottom left), and 20 ng/ml FGF4 (bottom right). Frame rate is 20 s, total duration is 82 min. Scale bar: 20 µm.

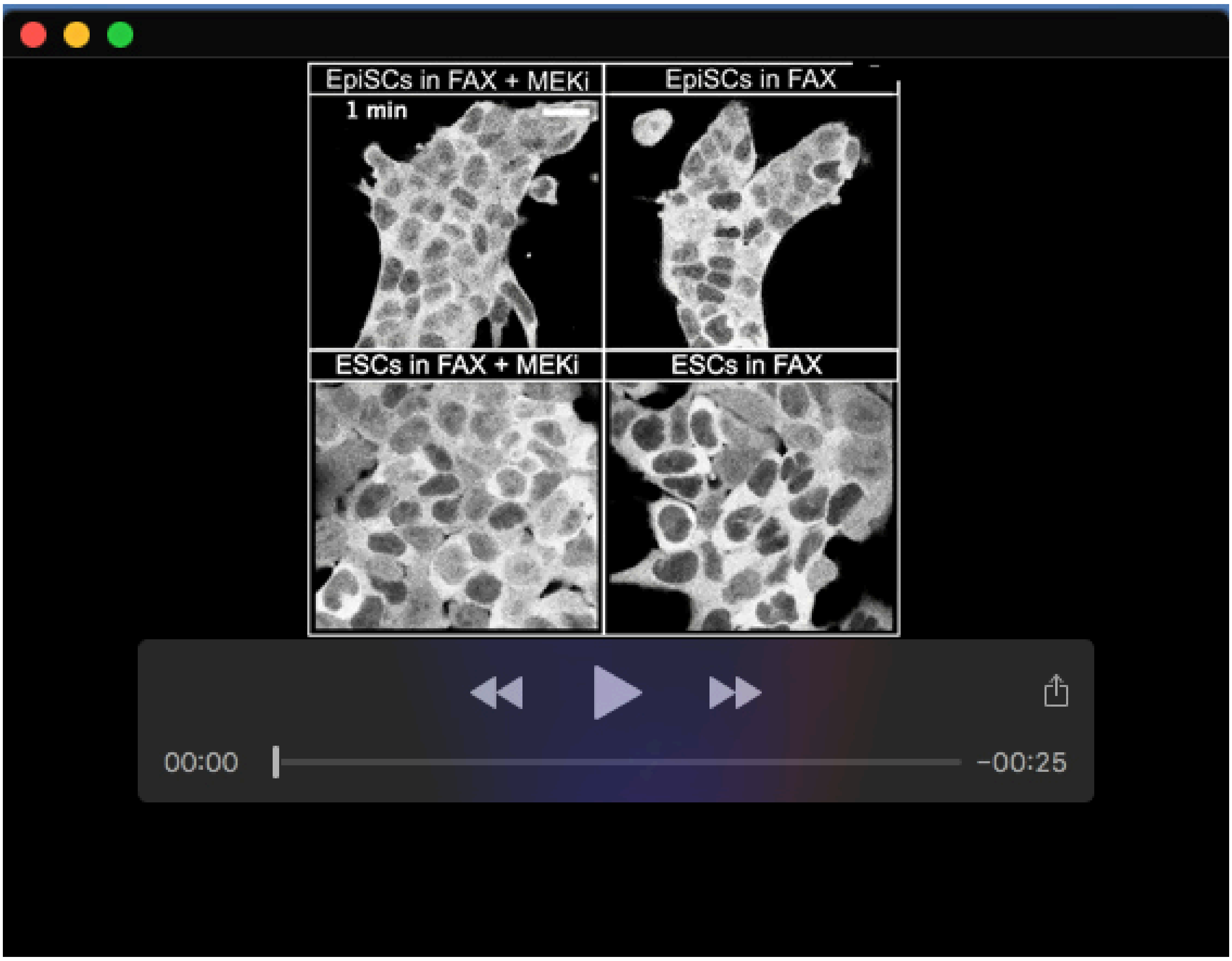

Movie 3. Dynamic ERK activity in EpiSCs. Time-lapse imaging of ERK-KTR expressing EpiSCs (top) and ESCs (bottom) cultured in FAX medium with (left) and without MEKi (right). Frame rate is 20 s, total duration is 205 min. Scale bar: 20 μm.
