# Supplementary material for: Intermittent ERK oscillations downstream of FGF in mouse embryonic stem cells
Source: Development. 2022 Feb 17;149(4):dev199710. doi: 10.1242/dev.199710 (PMC8918804; doi:10.1242/dev.199710)
Supplement: Reviewer comments [file develop-149-199710_review_history.pdf]

Intermittent ERK oscillations downstream of FGF in mouse embryonic stem cells

Dhruv Raina, Fiorella Fabris, Luis G. Morelli and Christian Schröter
DOI: 10.1242/dev.199710

Editor: James Briscoe

Review timeline
Submission to Review Commons: 21 December 2020
Submission to Development: 12 April 2021
Editorial decision: 14 April 2021
First revision received: 1 November 2021
Editorial decision: 13 December 2021
Second revision received: 29 December 2021
Accepted: 31 December 2021

Reviewer 1

Evidence, reproducibility and clarity

In this paper, Raina and colleagues describe the single cell ERK activity dynamics of mouse embryonic stem cells (ESCs). They show that single cell ERK signaling dynamics consist of pulses of identical amplitude and duration. Increasing doses of the growth factor FGF4 lead to increases in the number of ERK pulses. Strikingly, a pattern of high ERK pulse frequency in early versus late cell cycle entry is observed. These results add new information about single cell dynamics of the signaling pathways that control fate decisions in stem cell renewal and differentiation. This is important since this can give new insights about the stochasticity of fate decision signaling that is observed in stem cells.

The data presented in this work is of high quality, and relies on a set of simple but very well controlled experiments. The authors have set up an elegant quantitative framework to analyze the ERK time-series they observe. I think this paper is a good first step (among multiple recent papers) that tackle single cell signaling dynamics in stem cells in a highly quantitative fashion. However, I think that the paper lacks just a little bit of mechanistic insights to be highly relevant. Also, I think the authors also overinterpret some of the data, and surprisingly do not discuss the cell cycle data too much. Not being a stem cell expert, I think it would make sense to explicitly spell out if the experimental conditions the authors use, FGF4 stimulation, are related to self-renewal or differentiation fates.

Major comments

1. I think this manuscript would benefit from additional experiments that would give some mechanistic insights about the MAPK system in ESCs. Some avenues to explore could be:

a. A. What is the MAPK network structure that leads to these responses ? This is extensively discussed in the manuscript but remains highly speculative.
b. The authors could also explore how ERK dynamics are decoded into by example the cell cycle fate decision they observed (co-imaging with cdt or geminin moieties of the Fucci sensor). I was a little bit confused by this data. Has this been measured at a specific FGF4 concentration, or have all the FGF4 data from different conditions been pooled together ? Are different FGF4 concentrations inducing different probabilities to enter the cell cycle ?

© 2022. Published by The Company of Biologists under the terms of the Creative Commons Attribution License (https://creativecommons.org/licenses/by/4.0/).

c. A third low hanging fruit would be to compare self-renewal versus differentiation conditions.

Answering any of these questions would add depth to the manuscript and make it a strong candidate for publication.

2. The discussion about how the ERK dynamics might emerge from MAPK network structures remains highly speculative. I think the authors should tone down their claim that they have a system that shifts between oscillatory and non-oscillatory ERK behaviors. I do not think their current quantitative analysis of ERK time-series can formally prove this claim. Maybe stem cells simply also exhibit an excitable ERK pulsatile system as observed in epithelial cells (the work of Albeck).
3. I think that one factor that the authors completely overlooked is baseline ERK activity. When I look at Movie S2, 0 ng/ml FGF4, I already see pretty high ERK activity in the cells. This especially evident when compared to MEK inhibition in Fig.1D. This means that there might also be very high basal ERK activity (half the amplitude than an ERK pulse ?). How does such as sustained baseline ERK activity occur ? Is it important for ERK-dependent regulation of fate decisions. This is clearly not the case in epithelial cells (Albeck). The authors should clearly state/discuss this!
4. The authors overlook one paper about single cell ERK responses to FGF2 (PMID: 31777174). In this paper the single cell ERK responses to FGF2 are evaluated in PC12 cells. Different FGF2 doses induce different levels of sustained ERK activity. This behavior emerges from the both the intracellular wiring of the MAPK pathway, but also from competitive interactions between FGF2, the primary FGFR receptor and the HSPG co- receptor. Since FGF4 also involves FGFR/HSPG interactions, I could imagine that strong sustained basal ERK activity emerges through a similar mechanism. An attractive hypothesis is that FGF4 has different binding properties to the FGFR/HSPG system, allowing it to produce ERK pulses on top of a sustained basal ERK activity. This can be experimentally tested by chlorate treatment of cells to inhibit sulfation of HSPGs, and thus get rid of HSPG interactions.
5. As already mentioned above, a low hanging fruit might be to understand how ERK dynamics regulate a specific cell fate decision. It seems that commitment to specific cell fate decisions are decided at specific cell cycle stages. Could cell cycle dependent ERK dynamics be important for this ?

I think the data of this paper is solid, but the authors are clearly speculating a lot, and do not provide mechanistic data. Any set of experiments that would give more mechanistic insights in this dynamic signaling system (see point 1), would enhance the paper and make it a strong candidate for publication. Point 2: the authors should tone down the claim about oscillatory ERK dynamics (or show clear data that clearly supports this point - the reviewer understands this is difficult). In addition, I think the authors should explore the significance of the high baseline, sustained ERK activity (points 3-4). This would then make this paper acceptable for publication.

Significance

Significance of the advance:

This paper show single cell signaling dynamics in stem cells. This can provide mechanistic understanding about the heterogeneity of stem cell fate decisions.
Comparison of current existing knowledge: similar ERK dynamics have been shown in other cell systems. This paper remains descriptive compared to other publications documenting similar phenomena.
Audience: important for the stem cell biolog, signaling and cell biology community: BROAD audience.

Expertise: I feel confident in reviewing all the aspects of this paper.

Referees cross-commenting

For me it is still very important that the authors also discuss the very high basal ERK activity that they have in their system ! Where does it come from ? Such a high, sustained basal ERK activity is absent from other systems. I have mentioned which experiment should be done !

© 2022. Published by The Company of Biologists under the terms of the Creative Commons Attribution License (https://creativecommons.org/licenses/by/4.0/).

Reviewer 2

Evidence, reproducibility and clarity

The work seems very carefully carried out and controlled for experimental bias, which is very important when dealing with these time series data. I have no issues with this aspect of the study, although as indicated below- perhaps the data handling could be a little more intuitively explained.

Major issues:

The abstract and discussion emphasis the system being on the brink of oscillatory and non-oscillatory regimes. While this is very interesting, I think the biological emphasis of this needs to be brought out more in the text- and what might this mean for the biological system. Importantly, there is a lot of discussion about oscillations, but no explicit use of standard tools for formally characterising whether or not the system is actually oscillatory- eg. Fourier. This should be achievable with the existing data. It would improve the paper if this can be done, and is well within the capabilities of the group. It is possible I may have missed some explanation of why my suggestion is superfluous, in which case, please bring this out more in the text.

Minor issues:

Line 22 abstract- dynamic activity has been explored, although not at this time-resolution. I would reword this.
Line28/29 abstract- can you say anything about the significance of being on the brink of the transition point, and what it means for the biology, even a suggestion here would give the abstract a lift.

Intro line 81- pulses require FGF4, not necessarily driven by FGF4. The dependency I agree on totally, but the fluctuations might have a different mechanism- eg. Feedback to dampen ERK activity, not fluctuations in FGF4. In serum, I would argue/suggest there are probably few extrinsic fluctuations as the cells are bathed in signals.
For the biosensor cell line, the molecular genetics look very clean, and the cells appear to go germline, so this is better validated for general cell health and potential than most ESC imaging cell lines.

Check the y axis scale of Figure 2E- this looks a little high. If you summed all the bars, you would get more than 1, maybe?

Fig 2,S2- perhaps state the correlation value. Does figure this belong in the main text? Says something about the limits (or otherwise) of the signalling perhaps?

Line 195 "This suggests the presence of consecutive pulses, occurring immediately one after another". Not clear how this follows from the IPI. In general, I find this paragraph difficult. It might help if you can contextualise it biologically, to frame why it is important.

Line 292- I'm not sure "precision" is the correct word here. Unless to you can detect some characteristic underlying frequency? The discussion also talks about oscillations in some depth, but it is not clear we are looking at oscillations. With the exisiting data, it should be possible to get a concrete answer to this- Fourier/Wavelet or alternative, perhaps.

It looks to me (correct me if I am wrong) that it would be suitable to say that the probability of the pulse is regulated but amplitude is not. In other words, the signalling shows all or nothing behaviour- much like transcription in several systems- indeed, your observations provide a mechanism for all or nothing transcriptional behaviour, which to the best of my knowledge, is rather unresolved.

Significance

The dynamics of ERK signalling during development is a very topical issue, with a lot of uncharted territory. In this study, Schroter and colleagues characterise the dynamics of (novel) fast pulses in

© 2022. Published by The Company of Biologists under the terms of the Creative Commons Attribution License (https://creativecommons.org/licenses/by/4.0/).

mouse ES cells, and relate the dynamics of these pulses to the upstream FGF4 signal features. They identify heterogeneity in pulsing in the population and reveal contributing sources of this heterogeneity, providing data that show that pulsing is sensitive to cell cycle stage and that the occurrence of pulses, rather than their strength is how the FGF4 regulating is channelled.

The paper is very interesting and will be of strong interest to researchers interested in signalling mechanisms and their relationship to developmental biology and stem cells.

Reviewer expertise: cell heterogeneity, imaging, ES cells, gene expression, Dictyostelium, development

Referees cross-commenting

Both other reviews seem informed. I think all of us agree that the quantitative side of the "oscillation" behaviour could do with some firming up.
The issue where reviewer 1 is a bit tougher than reviewer 3 and myself is the need for another experiment. Of the three potential experiments suggested by reviewer 1, the self- renewal vs differentiation would require the least tool development, and might be the most interesting of the three.

I think discussing the baseline should be sufficient. I would rather not encourage the authors to carry out a drug experiment. These things rarely stand the test of time.

Reviewer 3

Evidence, reproducibility and clarity

In this study the authors characterise ERK dynamics in mouse ES cells in response to FGF signalling. Using a translocator-based sensor and time-lapse imaging, Erk is found to be activated in short pulses whose interpulse intervals are heterogeneous, with some pulses occurring consecutively, others occurring more sparsely and single cells observed to switch between various dynamic regimes over time. Increasing concentrations of FGF are found to increase pulsing, although the distributions of pulse durations and amplitudes are largely constant. In addition to FGF, the cell cycle and non-stationarity associated to experimental conditions are found to contribute to the heterogeneous dynamics.

The experiments and analyses are clearly presented, the paper is easy to follow and figures are nicely arranged. However, there are a few points that should be revised:

Major comments

1) If I understand correctly, the analyses are based only on the fluorescence at a small nuclear region rather than the C/N ratio (L. 130,L.577-579) used in the original KTR publication by Regot et al. The C/N ratio seems necessary to eliminate fluctuations due to variability in the sensor levels or other factors that might affect its overall fluorescence in a given cell. This could be particularly relevant for the long-term traces. Can the authors explain this choice? Can they show that C signals are constant over time and across cells so that the results would be the same if accounting for the cytoplasmic signal as well?

2) The authors mostly interpret their results in terms of oscillations (Abstract, L.209,L.374...). Although there is some clear oscillatory dynamics that can be seen by eye inspection of the timetraces, the current quantitative analysis is more consistent with stochastic pulsing than truly oscillations with periodicity.
First, pairs of consecutive pulses do not really inform on multiple, periodic pulses.

Second, the distribution of interpulse intervals is clearly asymmetric, a hallmark of stochastic pulsing.

As the authors suggest, the system seems to be close to a bifurcation that leads to oscillations, with FGF moving the system closer to the bifurcation point. The fact that pulse amplitude is quite

© 2022. Published by The Company of Biologists under the terms of the Creative Commons Attribution License (https://creativecommons.org/licenses/by/4.0/).

variable and correlated with duration (Fig. 2 supp 2) suggests that the system is most likely non-excitable, as also acknowledged by the authors in L. 434. PMID 30316816 might provide conceptual help to reason about this scenario.

Although it is really hard to distinguish noisy oscillations from stochastic pulses from finite data as the one here, some further analyses could be done to better characterize the extent to which the pulses are periodic. The following are ideas that might clarify on the presence of oscillations:

i) Determine the autocorrelation function for individual timetraces. The presence of signal at a defined lag can be indicative of periodicity, and the variability in these lag times could be indicative of the variability in the period. In addition, this may enable classifying timetraces according to their oscillating activities, and might also better characterise the longer timescale dynamics.

ii) Count number of consecutive pulses: 2,3,4,5,6... and plot the corresponding distribution for each condition.

Does it switch to the right with FGF? If so this would strengthen the idea that FGF increases the oscillatory dynamics, which is currently supported only by the narrowing of the IPI distribution (which keeps being asymmetric) and the increase in pairs of consecutive pulses (which may or may not be consecutive between themselves).

What happens over time in the long term data?

For each cell, this analysis could also be looked at as a heatmap where rows are cells, columns are numbers of consecutive pulses, and color is the number of instances.

The idea of comparing interpulse intervals across times as in PMID 28417973 might be helpful. This reference could also be added to the discussion as another example of system where dynamics can transit between regimes.

Depending upon the results of these analyses I would suggest revising the use of the word "oscillations" over the paper.

3) I would suggest revising lines 424-430. Although the mode is largely constant, the IPI distributions narrow so the overall frequency of the time traces increases, consistent with FM.

Minor points:

1) For completeness, can the authors show the distribution of interpulse intervals for the long-term data (even if IPI in this case would be defined differently).

Significance

The authors demonstrate ERK pulsing in mouse ES cells. Given the relevance of ERK signalling in this system and the relevance of ES cell research, this is an important finding that strengthens the need for understanding the dynamical properties of signal transduction pathways. The central role of ERK in cellular regulatory processes will make this interesting to a variety of audiences, including the stem cell community as well as the field of dynamics in signal transduction systems.

The authors could add additional references to signal-transduction systems that also show this richness of behaviour, like PMID 28417973 as indicated above.

My expertise is in computational systems biology, including the study of pulsatile/oscillatory dynamics in biological systems, and the analysis of ERK KTR data. As such, I cannot evaluate the experimental details of the work nor details pertaining to mES cells in particular.

© 2022. Published by The Company of Biologists under the terms of the Creative Commons Attribution License (https://creativecommons.org/licenses/by/4.0/).

Author response to reviewers' comments

Reviewer #1 (Evidence, reproducibility and clarity (Required)):

In this paper, Raina and colleagues describe the single cell ERK activity dynamics of mouse embryonic stem cells (ESCs). They show that single cell ERK signaling dynamics consist of pulses of identical amplitude and duration. Increasing doses of the growth factor FGF4 lead to increases in the number of ERK pulses. Strikingly, a pattern of high ERK pulse frequency in early versus late cell cycle entry is observed. These results add new information about single cell dynamics of the signaling pathways that control fate decisions in stem cell renewal and differentiation. This is important since this can give new insights about the stochasticity of fate decision signaling that is observed in stem cells.

The data presented in this work is of high quality, and relies on a set of simple but very well controlled experiments. The authors have set up an elegant quantitative framework to analyze the ERK time- series they observe. I think this paper is a good first step (among multiple recent papers) that tackle single cell signaling dynamics in stem cells in a highly quantitative fashion. However, I think that the paper lacks just a little bit of mechanistic insights to be highly relevant. Also, I think the authors also overinterpret some of the data, and surprisingly do not discuss the cell cycle data too much. Not being a stem cell expert, I think it would make sense to explicitly spell out if the experimental conditions the authors use, FGF4 stimulation, are related to self-renewal or differentiation fates.

We thank the reviewer for commending our analysis framework, and for highlighting the new insights that our data can give.

We also acknowledge the suggestion to spell out how the experimental conditions and FGF4 stimulation relate to self-renewal and differentiation, this will surely make the manuscript more accessible to a broader readership. In brief, the experiments described in figures 1 and 2 have been carried out in pluripotency medium that contains serum and LIF. For the experiments described in figures 3 and 4, cells were maintained in serum-free medium that contains Chiron and LIF to maintain pluripotency. However, LIF can activate ERK (PMID: 25564647). Therefore, to separate ERK dynamics downstream of FGF from other inputs into ERK activity, we removed LIF at the beginning of the recording. Thus, cells analyzed in figures 3 and 4 are pluripotent at the beginning of the recording, but will slowly differentiate under the influence of FGF signaling. The similar ERK dynamics observed in figures 2 and 3 indicate that the differences between pluripotency medium based on serum + LIF, and a defined medium in which cells slowly differentiate, do not have a strong influence on ERK dynamics. We will spell out how the experimental conditions relate to self-renewal and differentiation more clearly in a revised version of the manuscript.

We address the reviewer’s general comments on providing more mechanistic insight and discussing the cell cycle and data interpretation in our responses to the specific points below.

Major comments

1. I think this manuscript would benefit from additional experiments that would give some mechanistic insights about the MAPK system in ESCs. Some avenues to explore could be:

a. A. What is the MAPK network structure that leads to these responses ? This is extensively discussed in the manuscript but remains highly speculative.

b. The authors could also explore how ERK dynamics are decoded into by example the cell cycle fate decision they observed (co-imaging with cdt or geminin moieties of the Fucci sensor). I was a little bit confused by this data. Has this been measured at a specific FGF4 concentration, or have all the FGF4 data from different conditions been pooled together ? Are different FGF4 concentrations inducing different probabilities to enter the cell cycle ?

c. A third low hanging fruit would be to compare self-renewal versus differentiation conditions.

© 2022. Published by The Company of Biologists under the terms of the Creative Commons Attribution License (https://creativecommons.org/licenses/by/4.0/).

Answering any of these questions would add depth to the manuscript and make it a strong candidate for publication.

We thank the reviewer for these suggestions which we agree could all add depth to the work. However, in our experimental system, not all experiments are equally feasible to carry out in a reasonable timespan. This is exacerbated by the current working conditions imposed by the pandemic which continue to impose disruptions on the normal running of our labs, as acknowledged by the Editors of The Company of Biologists' journals (https://dev.biologists.org/content/news#covid-update). In a revision, we therefore propose to follow point (c) and focus on analyzing ERK dynamics in primed pluripotent cells. This is in line with Reviewer #2 comments and cross-commenting, and will address the question how these dynamics change as cells progress towards a more differentiated, epithelial state. This experiment can be carried out with the existing cell lines and analysis approaches, and is therefore feasible under the working conditions of the pandemic. In contrast, addressing the first two issues experimentally would require extensive reagent development, as rightfully acknowledged by Reviewer #2 who is a stem cell expert. We therefore propose to address the remaining issues through rewriting the text.

Concerning point (b), the Reviewer's suggestion to explore the relationship between ERK dynamics and cell cycle seems to stem from a confusion. The Reviewer queries whether the cell cycle data was obtained at any specific FGF4 concentration. The cell cycle data were "obtained in wild type cells growing in N2B27 medium, thereby exclusively focusing on pulsing driven by paracrine FGF4 signaling" -see lines 323-326 of the original manuscript. Furthermore, ESCs continue to cycle even in the absence of FGF stimulation. FGF4 concentration is therefore unlikely to control the probability to enter the cell cycle. In the revised manuscript, we will explicitly point out this feature of ESCs in line 241, and add quantifications of cell cycle lengths at different FGF4 concentrations to clear all doubt. We hope that with this addition we will avoid a potential confusion.

Concerning point (a), the Reviewer's comment on the MAPK network structure, it seems we have conveyed the idea that the work would address network players and structure, while our focus is rather on the dynamics produced by the underlying signaling network. We do think that network dynamics has a signature of this underlying structure. In this sense, our work builds the foundation to interrogate this structure in future work, but this is out of the scope of the manuscript. In a revised manuscript, we will make this line of reasoning clearer, and tone down any expectations that we would deliver new results on a specific network structure. Overall, the dynamical signatures that we report here point to a possibly general mechanism of how FGF controls Erk activity -and this constitutes a valuable mechanistic insight.

2. The discussion about how the ERK dynamics might emerge from MAPK network structures remains highly speculative. I think the authors should tone down their claim that they have a system that shifts between oscillatory and non-oscillatory ERK behaviors. I do not think their current quantitative analysis of ERK time-series can formally prove this claim. Maybe stem cells simply also exhibit an excitable ERK pulsatile system as observed in epithelial cells (the work of Albeck).

Reviewers #2 and #3 were convinced that there is oscillatory behavior in ESCs, and both proposed some additional approaches to further support this claim. We discuss a series of new analyses that we will carry out to probe the data for oscillations and to contrast it with purely noise-driven pulsing at length in our reply to Reviewer #3. Preliminary results from these analyses indeed support the presence of ERK oscillations in ESCs. We will update the text accordingly following these new analyses.

3. I think that one factor that the authors completely overlooked is baseline ERK activity. When I look at Movie S2, 0 ng/ml FGF4, I already see pretty high ERK activity in the cells. This especially evident when compared to MEK inhibition in Fig.1D. This means that there might also be very high basal ERK activity (half the amplitude than an ERK pulse ?). How does such as sustained baseline ERK activity occur ? Is it important for ERK-dependent regulation of fate decisions. This is clearly not the case in epithelial cells (Albeck). The authors should clearly state/discuss this!

The Reviewer interprets the higher C/N ratio of the reporter in Movie S2, 0 ng/ml FGF4 compared

© 2022. Published by The Company of Biologists under the terms of the Creative Commons Attribution License (https://creativecommons.org/licenses/by/4.0/).

to Fig. 1D as an indication of high ERK activity in the absence of ligand. The immunoblotting data and the results from a transcriptional reporter in Fig. 3 Supp. 1 do not support this interpretation. Rather, they indicate that the mapping between reporter C/N ratios and pERK levels is different between experimental conditions, and that chronic deprivation from FGF4 in the mutant line either increases basal nuclear sensor export or decreases its import. Thus, the reporter C/N ratio in the absence of ligand should be interpreted as a *sensor* baseline, but not ERK activity baseline. We will discuss this phenomenon in a revised manuscript when we introduce the Fgf4 mutant line (line 248 in the original manuscript).

4. The authors overlook one paper about single cell ERK responses to FGF2 (PMID: 31777174). In this paper the single cell ERK responses to FGF2 are evaluated in PC12 cells. Different FGF2 doses induce different levels of sustained ERK activity. This behavior emerges from the both the intracellular wiring of the MAPK pathway, but also from competitive interactions between FGF2, the primary FGFR receptor and the HSPG co-receptor. Since FGF4 also involves FGFR/HSPG interactions, I could imagine that strong sustained basal ERK activity emerges through a similar mechanism. An attractive hypothesis is that FGF4 has different binding properties to the FGFR/HSPG system, allowing it to produce ERK pulses on top of a sustained basal ERK activity. This can be experimentally tested by chlorate treatment of cells to inhibit sulfation of HSPGs, and thus get rid of HSPG interactions.

We thank the reviewer for pointing us to PMID:31777174. However, we would like to emphasize that that paper applies a fundamentally different stimulation paradigm compared to our study. While PMID: 31777174 uses pulsed FGF2 stimulation, we observed ERK pulses upon continuous stimulation with FGF4 together with constant high concentrations of heparin. This argues against the hypothesis that these pulses are generated through competitive interactions at the receptor level. Rather, our observations are more in line with models in the field that indicate pulsing emerges intracellularly (Albeck, PMID: 26304118). We therefore doubt that the chlorate experiment proposed by Reviewer #1 is promising in our system, as also pointed out in the referees cross-commenting by Reviewer #2 below.
We will of course discuss PMID: 31777174 to contrast possible origins of ERK dynamics in different systems.

5. As already mentioned above, a low hanging fruit might be to understand how ERK dynamics regulate a specific cell fate decision. It seems that commitment to specific cell fate decisions are decided at specific cell cycle stages. Could cell cycle dependent ERK dynamics be important for this ?

While these are clearly interesting ideas, we disagree with the Reviewer that connecting ERK dynamics with a cell fate decision is an easily doable experiment in ESCs. As we outline in our response to point 1. b above, ESCs continue to divide in the absence of FGF4 stimulation. Thus, the decision to enter the cell cycle is unlikely to be controlled by ERK dynamics. Other cell fate decisions, such as the exit from pluripotency, occur over timescales that are long relative to the ERK dynamics, and there are currently no markers that would allow unambiguously detecting this decision and that are compatible with live cell imaging. Finally, understanding how ERK dynamics regulate specific cell fate decisions would require new analysis frameworks. Such experiments are therefore beyond the scope of this manuscript, especially in the current pandemic situation. We argue that our work opens up these fresh lines for future research.

I think the data of this paper is solid, but the authors are clearly speculating a lot, and do not provide mechanistic data. Any set of experiments that would give more mechanistic insights in this dynamic signaling system (see point 1), would enhance the paper and make it a strong candidate for publication. Point 2: the authors should tone down the claim about oscillatory ERK dynamics (or show clear data that clearly supports this point - the reviewer understands this is difficult). In addition, I think the authors should explore the significance of the high baseline, sustained ERK activity (points 3-4). This would then make this paper acceptable for publication.

In summary, to address these general points, we propose to analyze ERK dynamics in more differentiated cells to investigate how the dynamic mechanism we propose here relates to cell state. We will carry out additional analyses to further test the oscillatory character of ERK activity, and modify the manuscript according to these new analyses. We will also clarify the sensor baseline

© 2022. Published by The Company of Biologists under the terms of the Creative Commons Attribution License (https://creativecommons.org/licenses/by/4.0/).

in the absence of ligand stimulation.

Reviewer #1 (Significance (Required)):

Significance of the advance:

This paper show single cell signaling dynamics in stem cells. This can provide mechanistic understanding about the heterogeneity of stem cell fate decisions.
Comparison of current existing knowledge: similar ERK dynamics have been shown in other cell systems. This paper remains descriptive compared to other publications documenting similar phenomena.
Audience: important for the stem cell biology, signaling and cell biology community: BROAD audience.

Expertise: I feel confident in reviewing all the aspects of this paper.

Referees cross-commenting

For me it is still very important that the authors also discuss the very high basal ERK activity that they have in their system ! Where does it come from ? Such a high, sustained basal ERK activity is absent from other systems. I have mentioned which experiment should be done !

Please see our response to points 3. and 4. above.

Reviewer #2 (Evidence, reproducibility and clarity (Required)):

The work seems very carefully carried out and controlled for experimental bias, which is very important when dealing with these time series data. I have no issues with this aspect of the study, although as indicated below- perhaps the data handling could be a little more intuitively explained.

We thank the reviewer for appreciating the design of experiments and analyses in our study. We realize that our data handling may be non-intuitive because the dynamics that we observe do not conform to a standard class of dynamics. In a revised version we will motivate the current analyses more intuitively on this background. In addition, we will expand our approach with more standard analyses suggested by the Reviewers, as we outline in detail below.

Major issues:

The abstract and discussion emphasis the system being on the brink of oscillatory and non-oscillatory regimes. While this is very interesting, I think the biological emphasis of this needs to be brought out more in the text- and what might this mean for the biological system. Importantly, there is a lot of discussion about oscillations, but no explicit use of standard tools for formally characterising whether or not the system is actually oscillatory- eg. Fourier. This should be achievable with the existing data. It would improve the paper if this can be done, and is well within the capabilities of the group. It is possible I may have missed some explanation of why my suggestion is superfluous, in which case, please bring this out more in the text.

The Reviewer raises two main points, one refers to the biological meaning of being on the brink of an oscillatory regime, the other to the use of standard tools for characterizing oscillations.

Being on the brink of an oscillatory regime could either mean that signaling heterogeneities generate the transcriptionally heterogeneous cell states that have been extensively described in ESCs.
Alternatively, it could be that the transcriptional state of cells positions the signaling system relative to the transition point. To address the Reviewer’s first point, we will briefly discuss these possibilities in the abstract, and expand on them in depth in the discussion section.

In their second point, the Reviewer suggests the use of standard tools to formally characterize whether or not the system is oscillatory. Visual inspection of the raw data suggests that it does not

© 2022. Published by The Company of Biologists under the terms of the Creative Commons Attribution License (https://creativecommons.org/licenses/by/4.0/).

conform to a standard type of dynamic behavior, therefore it is not clear a priori how well standard analyses perform. Prompted by the comments of reviewers #2 and #3, we realize however that more standard approaches could yield meaningful results on a subset of traces, and thereby help to formally identify oscillatory behavior.

Instead of Fourier analysis, we plan to include autocorrelation plots for individual traces in a revised manuscript. The autocorrelation function is related to the power spectrum (and Fourier transform) by the Wiener-Khinchin theorem, so they contain similar information. We favor the autocorrelation because we think that it has a more accessible interpretation in terms of timescales instead of frequencies. Preliminary analysis of autocorrelation functions indicates a noisy oscillatory behavior in a subset of cells. This approach, together with additional analyses to distinguish oscillatory from noise- driven dynamics, is further discussed in connection to Reviewer #3 comments (point 2i) and suggestions.

Minor issues:

Line 22 abstract- dynamic activity has been explored, although not at this time-resolution. I would reword this.

Thanks for pointing this out, we will reword the sentence to acknowledge previous studies that looked at dynamic activity at different time resolutions.

Line28/29 abstract- can you say anything about the significance of being on the brink of the transition point, and what it means for the biology, even a suggestion here would give the abstract a lift.

We will discuss the significance of being on the brink of a transition point towards oscillations as described in our response to the major points above.

Intro line 81- pulses require FGF4, not necessarily driven by FGF4. The dependency I agree on totally, but the fluctuations might have a different mechanism- eg. Feedback to dampen ERK activity, not fluctuations in FGF4. In serum, I would argue/suggest there are probably few extrinsic fluctuations as the cells are bathed in signals.

This is a good point, we realize that as it was written it was confusing. We will rewrite this sentence to clarify our observation that pulses are indeed likely generated by a cell-intrinsic mechanism, as pointed out by the Reviewer.

For the biosensor cell line, the molecular genetics look very clean, and the cells appear to go germline, so this is better validated for general cell health and potential than most ESC imaging cell lines.

We thank the Reviewer for appreciating this aspect of the study.

Check the y axis scale of Figure 2E- this looks a little high. If you summed all the bars, you would get more than 1, maybe?

The histograms have been normalized so that their total area is one. Because bars have a width, so to do the integral one should multiply the height of the bars by their width. It is this discretization of the integral that equals one. We will make this clear in the figure legend.

Fig 2,S2- perhaps state the correlation value. Does figure this belong in the main text? Says something about the limits (or otherwise) of the signalling perhaps?

We have refrained from interpreting the amplitude values throughout the manuscript at face value. The reason is that the quantitative relation between sensor pulse amplitude and ERK pulse amplitude is not known (see for example PMID 22114702). For the same reason we decided not to bring Fig. 2 Supp. 2 as a Main item in Fig. 2. From comments from all three Reviewers we realized that these limitations regarding sensor pulse amplitude should be highlighted more clearly in the manuscript.

© 2022. Published by The Company of Biologists under the terms of the Creative Commons Attribution License (https://creativecommons.org/licenses/by/4.0/).

Concerning a correlation value, fitting a line in this amplitude vs. duration plot would imply an underlying model of how these quantities relate to each other. However, we do not have such a model and we think that a simple correlation analysis might be misleading.

Line 195 "This suggests the presence of consecutive pulses, occurring immediately one after another". Not clear how this follows from the IPI. In general, I find this paragraph difficult. It might help if you can contextualise it biologically, to frame why it is important.

The interpretation of consecutive pulses follows from the relative durations of pulses and IPIs. We argue that the similarity of the modes of these distributions suggests that pulses are following immediately after another. We will spell this out more clearly in a revised version.

We appreciate that this paragraph may be unclear in general because we did not sufficiently motivate the main purpose of this section, which is to narrow down on the type of dynamics that the cells are producing. Classification of these dynamics can inform about the underlying network. In the revised version we will motivate the analysis along this line to provide the biological context.

Line 292- I'm not sure "precision" is the correct word here. Unless to you can detect some characteristic underlying frequency? The discussion also talks about oscillations in some depth, but it is not clear we are looking at oscillations. With the exisiting data, it should be possible to get a concrete answer to this- Fourier/Wavelet or alternative, perhaps.

We will drop the term precision to avoid confusions. It was only used once to reflect the narrowing of the IPI distributions with increasing FGF4 dose.
For the additional analyses that we plan to perform in order to distinguish noise-driven pulsing from oscillations see our response to comments from Reviewer #3.

It looks to me (correct me if I am wrong) that it would be suitable to say that the probability of the pulse is regulated but amplitude is not. In other words, the signalling shows all or nothing behaviour- much like transcription in several systems- indeed, your observations provide a mechanism for all or nothing transcriptional behaviour, which to the best of my knowledge, is rather unresolved.

This is an intriguing idea. However, in Figure 1A, B and Fig. 3 Supp 4 we detect pERK staining above background levels in almost all cells, suggesting that ERK signaling occurs also during silent periods and in between pulses. Thus, this would argue against the idea of all-or-nothing ERK activity.

Reviewer #2 (Significance (Required)):

The dynamics of ERK signalling during development is a very topical issue, with a lot of uncharted territory. In this study, Schroter and colleagues characterise the dynamics of (novel) fast pulses in mouse ES cells, and relate the dynamics of these pulses to the upstream FGF4 signal features. They identify heterogeneity in pulsing in the population and reveal contributing sources of this heterogeneity, providing data that show that pulsing is sensitive to cell cycle stage and that the occurrence of pulses, rather than their strength is how the FGF4 regulating is channelled.

The paper is very interesting and will be of strong interest to researchers interested in signalling mechanisms and their relationship to developmental biology and stem cells.
Reviewer expertise: cell heterogeneity, imaging, ES cells, gene expression, Dictyostelium, development

Referees cross-commenting

Both other reviews seem informed. I think all of us agree that the quantitative side of the "oscillation" behaviour could do with some firming up.

The issue where reviewer 1 is a bit tougher than reviewer 3 and myself is the need for another experiment. Of the three potential experiments suggested by reviewer 1, the self-renewal vs

© 2022. Published by The Company of Biologists under the terms of the Creative Commons Attribution License (https://creativecommons.org/licenses/by/4.0/).

differentiation would require the least tool development, and might be the most interesting of the three.

I think discussing the baseline should be sufficient. I would rather not encourage the authors to carry out a drug experiment. These things rarely stand the test of time.

As recommended by all three Reviewers, we will carry out an additional set of analyses to distinguish more clearly oscillatory behavior from noise-driven pulsing. In our response to comments from Reviewer #3 we describe how we will use autocorrelation analysis and pulse train statistics.
Additionally, we propose to contrast the experimental data with synthetic data generated from a stochastic pulsing model.

As suggested by the Reviewer, we will discuss the origin and meaning of the sensor baseline as detailed in our response to Reviewer #1's point.

We interpret the referees cross-commenting to indicate there is no experiment that would be absolutely necessary to substantiate our claims. However, to add weight the work and make it a stronger candidate for publication, we offer to follow the suggestion of Reviewers #1 and #2 and carry out an analysis of ERK signaling dynamics in primed pluripotent EpiSCs, as detailed in our response to Reviewer #1 above.

Reviewer #3 (Evidence, reproducibility and clarity (Required)):

In this study the authors characterise ERK dynamics in mouse ES cells in response to FGF signalling. Using a translocator-based sensor and time-lapse imaging, Erk is found to be activated in short pulses whose interpulse intervals are heterogeneous, with some pulses occurring consecutively, others occurring more sparsely and single cells observed to switch between various dynamic regimes over time. Increasing concentrations of FGF are found to increase pulsing, although the distributions of pulse durations and amplitudes are largely constant. In addition to FGF, the cell cycle and non- stationarity associated to experimental conditions are found to contribute to the heterogeneous dynamics.

The experiments and analyses are clearly presented, the paper is easy to follow and figures are nicely arranged. However, there are a few points that should be revised:

Major comments

1) If I understand correctly, the analyses are based only on the fluorescence at a small nuclear region rather than the C/N ratio (L. 130,L.577-579) used in the original KTR publication by Regot et al. The C/N ratio seems necessary to eliminate fluctuations due to variability in the sensor levels or other factors that might affect its overall fluorescence in a given cell. This could be particularly relevant for the long-term traces. Can the authors explain this choice? Can they show that C signals are constant over time and across cells so that the results would be the same if accounting for the cytoplasmic signal as well?

We decided to focus on a small nuclear region for analysis, because the cytoplasm in ESCs is small, and individual cells in colonies cannot easily be distinguished from each other in time-lapse movies. As the sensor construct is expressed from a single targeted genomic locus, the variability in overall fluorescence within a cell and between cells is relatively low. To further substantiate this choice, we will follow the Reviewer's suggestion to track both the cytoplasmic as well as the entire nuclear signal for some selected cells where cytoplasm can be unambiguously distinguished, across time. In a revised manuscript, we will show how these measurements of C/N fluorescence compare to 1/C measurements in a region of interest.

In long term traces, to eliminate fluctuations due to variability in sensor levels and other factors we had performed baseline correction using two alternative filtering strategies. We realise that although this was explained in Fig. 4 Supp. 2, we did not highlight this aspect in the Main text. We will point out these considerations in a revised version of the manuscript.

© 2022. Published by The Company of Biologists under the terms of the Creative Commons Attribution License (https://creativecommons.org/licenses/by/4.0/).

2) The authors mostly interpret their results in terms of oscillations (Abstract, L.209,L.374...). Although there is some clear oscillatory dynamics that can be seen by eye inspection of the timetraces, the current quantitative analysis is more consistent with stochastic pulsing than truly oscillations with periodicity.

First, pairs of consecutive pulses do not really inform on multiple, periodic pulses.

Second, the distribution of interpulse intervals is clearly asymmetric, a hallmark of stochastic pulsing.

As the authors suggest, the system seems to be close to a bifurcation that leads to oscillations, with FGF moving the system closer to the bifurcation point. The fact that pulse amplitude is quite variable and correlated with duration (Fig. 2 supp 2) suggests that the system is most likely non-excitable, as also acknowledged by the authors in L. 434. PMID 30316816 might provide conceptual help to reason about this scenario.

Although it is really hard to distinguish noisy oscillations from stochastic pulses from finite data as the one here, some further analyses could be done to better characterize the extent to which the pulses are periodic. The following are ideas that might clarify on the presence of oscillations:

i) Determine the autocorrelation function for individual timetraces. The presence of signal at a defined lag can be indicative of periodicity, and the variability in these lag times could be indicative of the variability in the period. In addition, this may enable classifying timetraces according to their oscillating activities, and might also better characterise the longer timescale dynamics.

Prompted by the reviewers suggestion, we have looked at the autocorrelation function of individual time traces and we observed a range of behaviors that nicely recapitulate the visual impression conveyed by the traces. Seemingly oscillatory traces have autocorrelation functions that show gradually decaying oscillations, signature of periodic oscillations with noise. Non-oscillating traces have a flatter autocorrelation function that decays faster, usually without oscillations.

We will include this analysis in the revised version as suggested by the Reviewer. We will also attempt a classification of traces using this approach. However, here we are skeptical about the outcome given the heterogeneity observed in many single traces.

ii) Count number of consecutive pulses: 2,3,4,5,6... and plot the corresponding distribution for each condition.

Does it switch to the right with FGF? If so this would strengthen the idea that FGF increases the oscillatory dynamics, which is currently supported only by the narrowing of the IPI distribution (which keeps being asymmetric) and the increase in pairs of consecutive pulses (which may or may not be consecutive between themselves).
What happens over time in the long term data?

For each cell, this analysis could also be looked at as a heatmap where rows are cells, columns are numbers of consecutive pulses, and color is the number of instances.

We thank the Reviewer for this good suggestion. We will definitely include a new analysis of longer pulse trains, defined as 2, 3, 4, … consecutive pulses. We will also use this approach to further interrogate the effect of FGF on the oscillatory dynamics. Unfortunately this analysis is not possible for the long term data, as the corresponding pulse detection algorithm does not map pulse minima, which are required to determine consecutiveness.

The idea of comparing interpulse intervals across times as in PMID 28417973 might be helpful. This reference could also be added to the discussion as another example of system where dynamics can transit between regimes.

© 2022. Published by The Company of Biologists under the terms of the Creative Commons Attribution License (https://creativecommons.org/licenses/by/4.0/).

We will further discuss reference PMID 28417973. We will also explore a comparison of interpulse intervals across times. However, we are concerned that the non-stationary experimental conditions together with cell-to-cell variability will confound this analysis.

Depending upon the results of these analyses I would suggest revising the use of the word "oscillations" over the paper.

We will revise the text in light of the new analyses, and discuss suggested references PMID 30316816 and PMID 28417973 to provide further context.

3) I would suggest revising lines 424-430. Although the mode is largely constant, the IPI distributions narrow so the overall frequency of the time traces increases, consistent with FM.

We realize that the signatures that we detect in our data such as the increase in pulse rate could be interpreted as an FM-component in ligand encoding. We will therefore revise our claims regarding FM encoding in this paragraph. Instead, we will focus more on contrasting the signatures of ERK pulsing in ESCs with those described in stochastically pulsing cell types. The new results of the different approaches described above will inform this interpretation of the data and the discussion of FM.

Minor points:

1) For completeness, can the authors show the distribution of interpulse intervals for the long-term data (even if IPI in this case would be defined differently).

We will be happy to include the distribution of IPIs for the long-term data.

Reviewer #3 (Significance (Required)):

The authors demonstrate ERK pulsing in mouse ES cells. Given the relevance of ERK signalling in this system and the relevance of ES cell research, this is an important finding that strengthens the need for understanding the dynamical properties of signal transduction pathways. The central role of ERK in cellular regulatory processes will make this interesting to a variety of audiences, including the stem cell community as well as the field of dynamics in signal transduction systems.

The authors could add additional references to signal-transduction systems that also show this richness of behaviour, like PMID 28417973 as indicated above.

My expertise is in computational systems biology, including the study of pulsatile/oscillatory dynamics in biological systems, and the analysis of ERK KTR data. As such, I cannot evaluate the experimental details of the work nor details pertaining to mES cells in particular.

Original submission

First decision letter

MS ID#: DEVELOP/2021/199710

MS TITLE: Intermittent ERK oscillations downstream of FGF in mouse embryonic stem cells

AUTHORS: Dhruv Raina, Fiorella Fabris, Luis G Morelli, and Christian Schröeter

Dear Dr. Schroeter,

Thank you for transferring the above manuscript from Review Commons. The manuscript and associated files can be accessed online: please go to BenchPress and click on the 'Manuscripts with Decisions' queue in the Author Area.

© 2022. Published by The Company of Biologists under the terms of the Creative Commons Attribution License (https://creativecommons.org/licenses/by/4.0/).

I have considered the manuscript and consulted with another Editor and we think that your study is potentially suitable for Development. If you are able to revise the manuscript along the lines you have outlined, I will be happy receive a revised version of the manuscript. Experimentally, including an analysis of ERK dynamics in differentiated cells, as you propose, would substantially strengthen the study. In addition, please include analyses to test whether the pulsatile behaviour is genuinely oscillatory and not stochastic fluctuations. I also agree that clarifying the origin of the baseline level of the ERK sensor will be important. Finally, for the benefit of Development readers, placing your findings in the context of the salt and pepper segregation of the ICM or in the definition of the epiblast identity in vivo would be very helpful. This could be achieved either by discussion or by reanalysing data from embryos.

Your revised paper will be re-reviewed by one or more of the original referees, and acceptance of your manuscript will depend on your addressing satisfactorily the reviewers' major concerns. Please also note that Development will normally permit only one round of major revision.

We are aware that you may be experiencing disruption to the normal running of your lab that make experimental revisions challenging. If it would be helpful, we encourage you to contact us to discuss your revision in greater detail. Please send us a point-by-point response indicating where you are able to address concerns raised (either experimentally or by changes to the text) and where you will not be able to do so within the normal timeframe of a revision. We will then provide further guidance. Please also note that we are happy to extend revision timeframes as necessary.

Please attend to all of the reviewers' comments and ensure that you clearly highlight all changes made in the revised manuscript. Please avoid using 'Tracked changes' in Word files as these are lost in PDF conversion. I should be grateful if you would also provide a point-by-point response detailing how you have dealt with the points raised by the reviewers in the 'Response to Reviewers' box. If you do not agree with any of their criticisms or suggestions please explain clearly why this is so.

Second decision letter

MS ID#: DEVELOP/2021/199710

MS TITLE: Intermittent ERK oscillations downstream of FGF in mouse embryonic stem cells

AUTHORS: Dhruv Raina, Fiorella Fabris, Luis G Morelli, and Christian Schröeter
ARTICLE TYPE: Research Article

I have now received the referees reports on the above manuscript, and have reached a decision. The referees' comments are appended below, or you can access them online: please go to BenchPress and click on the 'Manuscripts with Decisions' queue in the Author Area.

The overall evaluation is positive and we would like to publish a revised manuscript in Development, provided that the referees' comments can be satisfactorily addressed. Referee 2 raises an interesting idea about fitting a 2-state model to the pulsing data and the similarity to bursting models of transcription. I think this is worth considering and commenting on. Please attend to all of the reviewers' comments in your revised manuscript and detail them in your point-by-point response. If you do not agree with any of their criticisms or suggestions explain clearly why this is so.

Reviewer 1

Advance summary and potential significance to field

The authors performed a series of additional experiments to improve the quality of the article. To answer to my point one, they analyzed ERK signaling dynamics in differentiation conditions.

© 2022. Published by The Company of Biologists under the terms of the Creative Commons Attribution License (https://creativecommons.org/licenses/by/4.0/).

Moreover, they significantly improved their analysis of the nature of pulsing dynamics, describing its intermittent oscillatory behavior.
The paper is in its nature descriptive, not providing information regarding how oscillation/pulses are generated and if cells read and interpret signaling dynamics to produce cellular responses. However, the high quality technical ad methodological approach make this article a precious resource in the signaling dynamics field. The same group or other groups can start from this point to understand how these fast intermittent oscillations are produced and if cells translate the dynamic profiles into cellular responses, such as cell cycle or differentiation decisions.

Comments for the author

For the above reasons, I consider that paper as an excellent candidate for Development. No further experiments are needed from my side.

Reviewer 2

Advance summary and potential significance to field

I will not comment extensively on the manuscript as I already reviewed it positively at Review Commons. Since then there is a lot more data and the narrative is a lot more coherent. It is very interesting data, with the time resolution on ERK dynamics revealing many aspects of ERK biology in ESCs that more cumbersome sensors (eg. FRET) and systems (eg. the embryo) can not reveal. In particular, the data reveal oscillations that our FRET data could not pick up (although we looked very hard) and characterises the nature and regulation of these pulses in depth. Specifically, the near all-or-nothing (all or occasional is probably more accurate) behaviour is very interesting, and may relate in a simple way to downstream transcriptional dynamics. The work is ready for publication. I make a few suggestions that can be included at the discretion of the authors.

Jonathan Chubb
Comments for the author

Line 207- should the autocorrelation data not be in the main figures? You mention oscillations a lot- perhaps this should be up front?
Modelling line c226: The modelling is very interesting. I'm wondering if you might get a better fit with a 2 state model- much like people use to model transcriptional bursting. So cells pulse or do not pulse, with 2 different pulsing probabilities, then flip between the two states with a certain frequency.
I'm not a fan of modelling just to get a fit, so maybe this is something to comment on rather than do.
Related to this. In Figure 3: this near all-or-nothing (ie. "on" when your "on" to the same degree, regardless of the stimulus intensity) behaviour is so reminiscent of transcription of many developmental genes- and maybe provides a mechanism for such transcriptional behaviour. It would be a shame not to comment on this.

Second revision

Author response to reviewers' comments

Thank you very much for sending the reviews on our revised manuscript. Both reviewers recommend publication of the manuscript in its current form in Development. Reviewer #2 has made additional suggestions, to be included at the authors' discretion.

The reviewer's first suggestion is to show the autocorrelation analysis in the main figures instead of the supplementary material. We have deliberately decided against this option. The autocorrelation analysis is consistent with our interpretation of intermittent oscillations, but it cannot formally rule out other dynamical behaviors. In contrast, the simulations of stochastic models clearly argue against simple stochastic behavior. The placement of the autocorrelation analysis in the

© 2022. Published by The Company of Biologists under the terms of the Creative Commons Attribution License (https://creativecommons.org/licenses/by/4.0/).

supplementary material and the simulations in the main figure reflects this different discriminatory power of the two approaches.

The reviewer's second suggestion is to fit a two-state model to the data. However, the reviewer acknowledges that adding such an analysis just for the sake of providing a model that fits the data will add little additional insight to the paper. Instead, as also pointed out by reviewer #1, the high quality data in our manuscript constitutes a precious resource to pursue more systematic theoretical approaches in the future.

Finally, reviewer #2 suggests discussing possible links between intermittent ERK oscillations and transcriptional dynamics of developmental genes. Following the reviewers suggestion, we have added a sentence and a reference in the discussion (lines 475 - 478, see red highlighting).

We hope that we have appropriately attended to the reviewer's suggestions.

Third decision letter

MS ID#: DEVELOP/2021/199710

MS TITLE: Intermittent ERK oscillations downstream of FGF in mouse embryonic stem cells

AUTHORS: Dhruv Raina, Fiorella Fabris, Luis G Morelli, and Christian Schröeter
ARTICLE TYPE: Research Article

Thank you for sending your manuscript to Development through Review Commons.

I am happy to tell you that your manuscript has been accepted for publication in Development, pending our standard ethics checks.

© 2022. Published by The Company of Biologists under the terms of the Creative Commons Attribution License (https://creativecommons.org/licenses/by/4.0/).
